# Development and Validation of Methodologies for the Identification of Specialized Pro-Resolving Lipid Mediators and Classic Eicosanoids in Biological Matrices

**DOI:** 10.1021/jasms.4c00211

**Published:** 2024-09-10

**Authors:** Matthew Dooley, Amitis Saliani, Jesmond Dalli

**Affiliations:** † Biochemical Pharmacology, William Harvey Research Institute, Barts and The London Faculty of Medicine and Dentistry, Queen Mary University of London, Charterhouse Square, London EC1M 6BQ, United Kingdom; § Centre for Inflammation and Therapeutic Innovation, Queen Mary University of London, London E1 4NS, United Kingdom

**Keywords:** Eicosanoids, lipid mediators, specialized pro-resolving mediators, liquid chromatography-tandem mass spectrometry

## Abstract

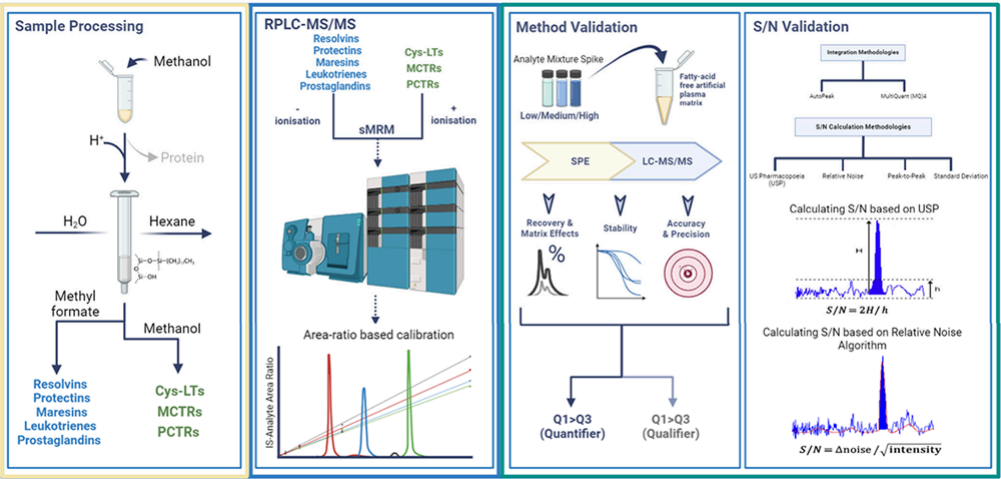

Lipid mediators, which include specialized pro-resolving mediators and classic eicosanoids, are pivotal in both initiating and resolving inflammation. The regulation of these molecules determines whether inflammation resolves naturally or persists. However, our understanding of how these mediators are regulated over time in various inflammatory contexts is limited. This gap hinders our grasp of the mechanisms underlying the disease onset and progression. Due to their localized action and low endogenous levels in many tissues, developing robust and highly sensitive methodologies is imperative for assessing their endogenous regulation in diverse inflammatory settings. These methodologies will help us gain insight into their physiological roles. Here, we establish methodologies for extracting, identifying, and quantifying these mediators. Using our methods, we identified a total of 37 lipid mediators. Additionally, by employing a reverse-phase HPLC method, we successfully separated both double-bond and chiral isomers of select lipid mediators, including Lipoxin (LX) A_4_, 15-epi-LXA_4_, Protectin (PD) D1, PDX, and 17R-PD1. Validation of the method was performed in both solvent and surrogate matrix for linearity of the standard curves, lower limits of quantitation (LLOQ), accuracy, and precision. Results from these studies demonstrated that linearity was good with *r*^2^ values > 0.98, and LLOQ for the mediators ranged from 0.01 to 0.9 pg in phase and from 0.1 to 8.5 pg in surrogate matrix. The relative standard deviation (RSD) for inter- and intraday precision in solvent ranged from 5% to 12% at low, intermediate, and high concentrations, whereas the RSD for the inter- and intraday variability in the accuracy ranged from 95% to 87% at low to high concentrations. The recovery in biological matrices (plasma and serum) for the internal standards used ranged from 60% to 118%. We observed a marked ion suppression for molecules evaluated in negative ionization mode, while there was an ion enhancement effect by the matrix for molecules evaluated in positive ionization mode. Comparison of the integration algorithms, namely, AutoPeak and MQ4, and approaches for calculating signal-to-noise ratios (i.e., US Pharmacopeia, relative noise, peak to peak, and standard deviation) demonstrated that different integration algorithms tested had little influence on signal-to-noise ratio calculations. In contrast, the method used to calculate the signal-to-noise ratio had a more significant effect on the results, with the relative noise approach proving to be the most robust. The methods described herein provide a platform to study the SPM and classic eicosanoids in biological tissues that will help further our understanding of disease mechanisms.

## Introduction

Lipid mediators play a central role in the regulation of a large array of biological processes from sleep to barrier function and immune responses.^1−7^ Despite intense research over many decades, the biological functions of many of these molecules and the mechanisms that control their activities remain to be fully elucidated. Among the large diversity of lipid mediators of interest are the classic eicosanoids that include the prostaglandins, leukotrienes, and thromboxane. These molecules regulate fundamental biological processes such as sleep and barrier function and are contributors to disease by catalyzing the propagation of inflammation.^1,6,7^ Another family of mediators that is relevant is the specialized pro-resolving lipid mediators (SPMs). These mediators are implicated in the regulation of both immune responses and stromal cell biology to limit the onset and propagation of inflammation.^2−5^ All of these molecules are produced via the stereoselective oxygenation of C20 and C22 polyunsaturated fatty acids by dedicated enzymes that primarily from part of the cyclooxygenase (COX), lipoxygenase (LOX), and cytochrome P450s (CYP) families.^8,9^

A key characteristic of these molecules is that their biological activities are primarily mediated via the activation of cognate receptors that are usually expressed on the surface of target cells.^10−12^ These molecules bind to their receptors with very high affinity, an observation which is likely linked to their relatively low abundance in biological systems, with concentrations in the picomolar to nanomolar ranges.^5,7,13−15^ Such low concentrations together with the large diversity of molecular species and physical characteristics make the identification and quantitation of these molecules challenging. This issue is further compounded by the diversity of methodologies used to identify and quantify lipid mediators, which have led to contrasting results.

Methodologies for the calculation of signal-to-noise ratios vary between vendors and applications, with, for example, the recently released Sciex OS software offering three integration algorithms and three methods for calculating signal-to-noise ratios. Relevantly, the influence of such a range of methodologies on lipid mediator identification has, to the best of our knowledge, not been evaluated. Given (i) the large heterogeneity in tools being used to measure these critical parameters, (ii) the relevant paucity by which the application of such tools is reported in publications, and (iii) the low concentrations of lipid mediators in biological systems, it is essential that methodologies used in their identification are rigorously characterized to identify robust approaches that can facilitate data reproducibility.

Thus, we sought to develop a robust method for the identification and quantification of SPM and classic eicosanoids in biological systems. For this purpose, we evaluated different extraction methodologies, developed and validated a RP-HPLC tandem mass spectrometry method, and assessed the robustness of different integration algorithms as well as approaches utilized in determining signal-to-noise (S/N) values. The results from these studies demonstrated that C18 SPE methodologies gave the most robust results for lipid mediator extraction, and C18-based RP-HPLC enabled the separation of both chiral and double-bond isomers of the SPM and their related arachidonic acid-derived eicosanoids. We observed that post extraction, these mediators displayed surprising stability when stored at 4 °C. Furthermore, we observed that selection of approaches for the determination of signal-to-noise ratios has a remarkable impact on the identification of these molecules.

## Methods

### Identification of Multiple Reaction Monitoring Transitions for Each of the Lipid Mediators

To identify multiple reaction monitoring transitions, we infused synthetic standards and selected up to six potential daughter ions from the MS/MS spectrum for each of the mediators. We selected ions that displayed the greatest intensity in the MS/MS spectrum and were either not shared with other closely eluting mediators or more abundant in the MS/MS spectrum for the mediator of interest when compared with those observed for closely eluting mediators. We then used the manual tuning mode in Analyst 1.6.3 to identify the optimal mass spectrometer parameters (i.e., declustering potential, entrance potential, collision energy, collision cell exit potential) for each ion pair. From these, we selected the ions that gave the strongest signal for further evaluation. Table S1 reports the origin of the standards used, and Table S2 reports the ion pairs that were used for further evaluation together with the respective instrument parameters. Table S3 reports the source parameters used. In these studies, positive mode ionization was used for peptide–lipid conjugated mediators, whereas negative mode ionization was used for the nonpeptide conjugated mediators.

### Chromatography

The chromatographic method employed was based on published methodologies with select modifications.^16^ For the analysis of peptide–lipid conjugated mediators, namely, the cysteinyl leukotrienes (cysLTs), maresin conjugates in tissue regeneration (MCTRs), and protectin conjugates in tissue regeneration (PCTRs), we used a Shimadzu LC-20AD HPLC equipped with an Agilent Poroshell 120 EC-C18 column (100 mm × 4.6 mm × 2.7 μm) paired with a Shimadzu SIL-20AC autoinjector and a QTrap 6500+ (Sciex). The initial mobile phase was methanol/water/acetic acid of 20:80:0.5 (v/v/v) which was ramped to 55:45:0.5 (v/v/v) over 0.2 min and maintained for 0.9 min, ramped to 70:30:0.5 (v/v/v) over 4.9 min and maintained for 2 min, and ramped to 80:20:0.5 (v/v/v) for the next 2 min. The mobile phase was then maintained for 3 min, ramped over 0.1 min to 98:2:0.5 (v/v/v), and maintained for 2.9 min. Following this, it was ramped in 0.1 min to 20:80:0.05 (v/v/v) and maintained for the subsequent 1.9 min.

In the analysis of the DHA-derived resolvins (RvD), protectins (PD), and maresins (MaR), *n* – 3 DPA-derived d-series resolvins (RvD_*n*–3 DPA_), 13-series resolvins (RvT), protectins (PD_*n*–3 DPA_), and maresins (MaR_*n*–3 DPA_), the EPA-derived resolvins (RvE), and the AA-derived lipoxins (LX), Leukotriene B_4_ (LTB_4_), prostaglandins (PG), and thromboxane (TX) B_2_, we used the instrumentation and column described above. The initial mobile phase consisted of methanol/water/acetic acid of 20:80:0.01 (v/v/v) that was ramped to 50:50:0.01 (v/v/v) over 0.2 min and maintained for 1.8 min, ramped to 80:20:0.01 (v/v/v) over 9 min and maintained for 3.5 min, and then ramped to 98:2:0.01 (v/v/v) for the next 0.1 min. This was subsequently maintained at 98:2:0.01 (v/v/v) for 5.4 min, after which it was rapidly decreased to 20:80:0.01 over 0.1 min and maintained for 3.0 min.

### Standard Curve Preparation

For mediators that carry UV chromophores, concentrations were determined using UV–vis spectroscopy (Agilent Technologies Cary 8454 UV–vis) and the extinction coefficients described in Table S2. The extinction coefficients are in accordance with those used in the literature to estimate the concentrations of lipid mediators with diene, triene, and tetraene conjugated double-bond systems.^17−19^ In instances where the mediator did not carry a UV chromophore (i.e., PG and TXB_2_), we used the concentration reported by the manufacturer. We prepared a stock mixture in methanol of these at a concentration of 100 pg/μL. This was then employed to construct 10-point calibration curves ranging between 0.05 and 125 pg. To each point in the standard curve we added 500 pg of the internal standard mix (with the exception of *d*_5_-17R-RvD1 which was used at 100 pg). This was added to both facilitate the identification of the mediators as well as their quantitation. For quantitation, we used the ratio of the area under the curve of the relevant internal standard (which was a constant through the standard curve) to that of the mediator of interest.

For standard curves prepared in artificial matrix, we extracted 500 μL of artificial matrix^20^ using methodologies described in the solid-phase extraction section below. We evaporated the solvent under a gentle stream of nitrogen using a TurboVap LV system (Biotage) and added each of the curve points in methanol. The solution was then vortexed for 10 s. In these experiments, the spiked analyte mixture was not itself subjected to SPE. The influence of the extraction procedure was evaluated in subsequent validation experiments that assessed the accuracy and precision of the methodology by spiking the artificial plasma prior to extraction (see Determination of Precision, Accuracy, and Carry Over).

Limits of detection (LOD) and lower limits of quantitation (LLOQ) were determined using formulas previously described^21,22^ with a minor modification in the factor employed for the determination of LLOQ, where we used a factor of 5 instead of 10 to reflect the LLOQ cutoff of a s/n = 5 as proposed by others.^23^ The LOD was determined as LOD = 3(*s/b*), while LLOQ = 5(*s/b*), where *s* is the standard deviation of the blank signal and *b* is the calibration graph slope.

### Evaluation of Lipid Mediator Stability

#### Stability at 4 °C

Stability was evaluated following published protocols.^24^ Pooled human plasma was extracted according to the C18 Solid-Phase Extraction section. Solvent was then evaporated; lipid mediators were resuspended in phase (1:1 MeOH/H_2_O) and spiked with a 8 pg of standard mix. These samples were then placed at 4 °C in the LC autosampler rack for 1, 3, 14, or 21 days. Prior to injection, internal standards were added and lipid mediators were identified based on (1) matching the retention time to the relevant standard and (2) a peak in the primary transition with a s/n value ≥ 5, and (3) a peak secondary transition with a s/n value ≥ 3. Values obtained represent the mean of five replicate injections. An entire replicate was excluded due to technical issues with sample injection.

#### Stability Following Freeze–Thawing

Human serum (Sigma H4522) was thawed gently on ice, and three 500 μL aliquots were placed in 2 mL of ice-cold methanol containing deuterium-labeled IS. This was then placed at −20 °C for 45 min to allow for protein precipitation, and lipid mediators were extracted as detailed in the C18 Solid-Phase Extraction section. The remaining serum volume was transferred to −80 °C. After 24 h, the serum was rethawed on ice, three 500 μL aliquots were placed in 2 mL of ice-cold methanol, proteins were precipitated, and lipid mediators were extracted as detailed above.

### Lipid Mediator Extraction

To evaluate whether the volume of methanol used has an influence on either lipid mediator ionization in the matrix or sample recovery, we placed human serum (0.5 mL) in either 1 or 2 mL of ice-cold methanol for 1 h containing deuterium-labeled IS. These samples were then incubated for 45 min at −20 °C and then subjected to C18 solid-phase extraction as detailed below. Extraction recoveries for each of the deuterium-labeled IS were calculated as a percentage of pre-extraction IS-spiked signal to postextraction IS-spiked signal. Ion suppression (matrix effect) was calculated as a percentage of post-extraction IS-spiked signal to IS containing methanol.

### C18 Solid-Phase Extraction

Following the addition of methanol (2 or 4 volumes), samples were held at −20 °C for at least 45 min to allow for protein precipitation. Samples were then centrifuged at 2000*g* for 5 min at 4 °C. Prior to initiating solid-phase extraction procedures, the volume of methanol was brought to <1 mL by evaporating under a gentle stream of nitrogen. Samples were then placed into round-bottom borosilicate tubes and loaded onto a Biotage Extrahera. The ISOLUTE C18 (500 mg/6 mL) columns were then conditioned using 6 mL of methyl formate followed by 6 mL of methanol. These were washed with 6 mL of deionized water. Samples were acidified using acidified water (pH 3.5) and bringing the volume up to 10 mL. These were rapidly loaded onto the conditioned C18 columns. Samples were allowed to run through the column; these were washed with 2 mL of neutral pH water followed by 6 mL of hexane. Lipid mediators, namely, RvD, PD, MaR, RvD_*n*–3 DPA_, RvT, PD_*n*–3 DPA_, MaR_*n*–3 DPA_, RvE, LX, LTB_4_, PG, and TXB_2_, were eluted using 6 mL of methyl formate, whereas the peptide–lipid conjugated mediators (i.e., cysLTs, PCTR, and MCTRs) were eluted using 6 mL of methanol. Solvents were then evaporated under a gentle stream of nitrogen using a TurboVap LV system and resuspended in 50 μL of water/methanol (50:50).

### Comparison of Extraction Methodologies

In a comparison of different extraction methodologies, we employed human plasma as the matrix of choice. Proteins were precipitated using the solvents detailed in Table S7, and samples were placed at −20 °C for a minimum of 45 min. Extractions were performed by using the conditions illustrated in Table S7 and a manual manifold. The different matrices were tested on separate occasions using condition A as the control condition in every experiment.

### Data Analysis

#### Comparison of Integration and Signal-to-Noise Algorithms

To evaluate integration and signal-to-noise algorithms on lipid mediator identification, we focused on the algorithms present in Sciex OS 3.1. In these studies, we used Autopeak and MultiQuant (MQ)4 integration algorithms and the relative noise, peak to peak, and standard deviation approach for calculating the signal-to-noise ratios. The latter were compared to the approach recommended by the US Pharmacopeia.^25^ As the primary aim of the study was to evaluate the influence of the different methodologies on lipid mediator identification, we evaluated the performance of these algorithms on the identification of lipid mediators within an artificial matrix that was spiked with 1.5 pg of the standard mix (per sample). We then generated qmethod files to compare signal-to-noise ratio values with different integration algorithms and signal-to-noise methodologies: Autopeak-relative noise, Autopeak-peak to peak, Autopeak-standard deviation, MQ4-relative noise, MQ4-peak to peak, and MQ4-standard deviation. Unless otherwise stated, we used the “low” smoothing setting when using the Autopeak algorithm and a Gaussian smooth width of 1.0 points and a noise percentage at 40% when using the MQ4 algorithm as recommended in the Sciex OS user guide.

To calculate the signal-to-noise ratios using the approach recommended by US Pharmacopoeia,^25^ we used the following formula , where *H* = height of the selected peak and *h* = range of the noise in the chromatogram within the blank injection or within the chromatogram where the peak of interest is present. When calculating the signal-to-noise ratios using either the peak-to-peak or standard deviation algorithm, the noise region was selected as the region immediately adjacent and of equivalent width to the peak of interest. The algorithm employed to calculate the noise in the relative noise method is (. The algorithm compares the signal intensity to the noise level within a specific time window or mass range. Unlike traditional methods that use a fixed noise level, the relative noise algorithm dynamically adjusts the noise estimation based on local variations. This provides a more accurate and context-sensitive measure of the signal-to-noise ratio, thereby improving the differentiation of true signals from background noise, particularly in complex or low-abundance samples. The unit of noise is counts per second (cps).

We observed that the area under the curve (AUC) for the peak corresponding to the mediator of interest varied between different integration algorithms when automatically integrated with the software. To improve the reproducibility of the AUC across different methodologies, we performed peak picking manually. This manual integration ensured more consistent results.

#### Evaluation of Smoothing on Signal-to-Noise Calculations

To explore the influence of smoothing on the calculation of the signal-to-noise ratios, we used the same samples as employed in the evaluations described above. These were integrated using either Autopeak or MQ4, and we used the relative noise algorithm to determine the signal-to-noise ratios. To evaluate the influence of smoothing on the signal-to-noise ratios, for the samples integrated using the Autopeak algorithm data was integrated with or without the “low” smoothing setting being selected, whereas for data integrated using the MQ4 algorithm, data was integrated using a Gaussian Smooth Width of either 1 (smoothed) or 0 (non-smoothed).

#### Determination of Precision, Accuracy, and Carry Over

Validation experiments were performed in both solvent (methanol) and the matrix. For experiments performed in solvent, standards were prepared at low (1.5 pg), intermediate (13.5 pg), or high (41.65 pg) concentrations. These were injected on to the LC-MS/MS system, and the precision and accuracy of the resultant signals was determined.

Due to the challenge of obtaining a blank matrix, experiments were performed using a surrogate matrix, prepared in accordance with European Standard EN ISO 10993-15:2009. This comprised NaCl (6.8 g/L), CaCl_2_ (0.20 g/L), KCl (0.40 g/L), MgSO_4_ (0.10 g/L), NaHCO_3_ (2.20 g/L), Na_2_HPO_4_ (0.126 g/L), NaH_2_PO_4_ (0.026 g/L), and 60 g/L of fatty acid-free bovine serum albumin (BSA, Sigma-Aldrich, St. Louis, MO, USA). This matrix was spiked with either a low (1.5 pg), intermediate (13.5 pg), or high (41.7 pg) concentration of the internal standard mix. To validate the methodology, these samples were subjected to C18 solid-phase extraction as detailed above, and then the precision and accuracy of the resultant signals in LC-MS/MS was evaluated.

To determine carry over, we injected a mediator mixture at 125 pg followed by a blank sample. This was performed in triplicate, and the signal in the blank samples was evaluated to determine whether there was a peak that eluted at the same retention time as the mediator of interest in each of the transitions evaluated.

#### Identification of Lipid Mediators in Standard Reference Material and Healthy Volunteer Plasma

Frozen standard reference human serum (NIST SRM-909c) was obtained from NIST and stored at −80 °C prior to experiments. An aliquot was then thawed on ice, vortexed to ensure homogeneity, and aliquotted into four aliquots of 500 or 100 μL, whereas plasma was collected from healthy volunteers in acidified sodium citrate as previously described.^26^ To these, 4 volumes of methanol containing deuterium-labeled internal standards were added, proteins were precipitated, and lipid mediators were extracted as detailed above. Each lipid mediator was identified using the following criteria: (1) matching retention time to synthetic or authentic standards (±0.05 min), (2) a signal-to-noise ratio ≥ 5 for a primary transition, and (3) a signal-to-noise ratio of ≥3 for a secondary transition. Data was analyzed using Sciex OS v3.0 or v3.1, chromatograms were reviewed using the AutoPeak algorithm using the “low” smoothing setting, and signal-to-noise ratios were calculated using the relative noise algorithm. External calibration curves were used to quantify the identified mediators.

## Results

### Identification and Characterization of MRM Transitions

We first identified MRM transitions that gave the strongest signal when the standard reference material was directly infused using methanol, water, and acetic acid (80/20/0.01%) as the mobile phase. Table S2 reports the list of MRMs that were selected for further evaluation for each of the mediators.

**Figure 1:**
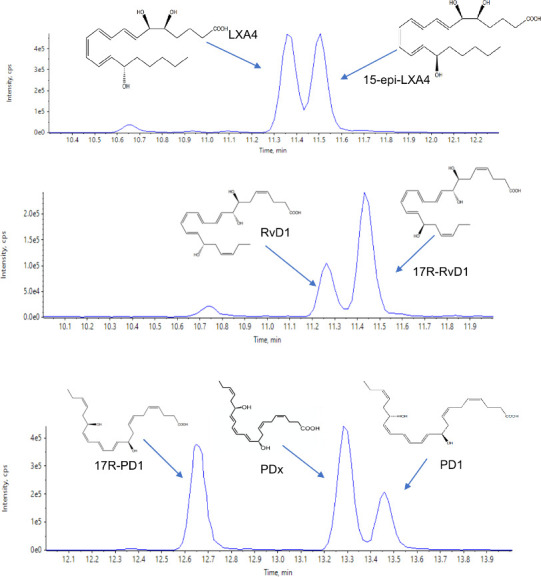
Chromatographic separation of double-bond and chiral isomers of SPMs. Multiple reaction monitoring chromatograms illustrating the separation of chiral and double-bond isomers of each of the SPMs.

The activity of lipid mediators, including SPM, is related to their structures. In biological systems, many of these molecules are present with several stereoisomers.^9^ Therefore, it is essential that methodologies developed to identify and quantify these mediators discern between the target mediator and its related isomers. C18 columns are widely used for the chromatographic separation of lipid mediators.^15,20,27^ Consequently, we explored whether using C18-based columns we could separate lipid mediator stereoisomers. Using a mobile phase composed of water, methanol, and acetic acid and enantiomerically pure standards obtained using total organic synthesis (see Table S1), we observed a separation in the peaks obtained with the 17-*R* isomers of RvD1, RvD3, and PD1 and their respective 17-*S* isomers (Figure 1). Similarly, this chromatographic separation was obtained for the 15-*R* isomers of LXA_4_ and LXB_4_ from their 15-*S* isomers. The separation of stereoisomers was also observed when evaluating the double-bond isomers of these molecules, whereby we observed a separation in the peaks obtained for PD1 and PDx (Figure 1). Together, these findings suggest no interference between the target analytes of this method.

### Standard Curves, LOD, and LLOQ

To facilitate lipid mediator quantitation and determine both the LLOQ and the LOD of each of the transitions, we prepared standard curves. Depending on sample complexity (e.g., cell incubations in PBS vs plasma), the matrix may have a significant influence on compound ionization and therefore both LOD and LLOQ. Therefore, we prepared the standard curves in both phase (50:50 methanol:water), representing samples with limited to no discernible matrix, and in a surrogate matrix that is commonly used to represent a plasma matrix (European Standard EN ISO 10993-15:2009 and ref (20)). We also used an ion ratio approach where the ratio for the area under the curve (AUC) of the mediator of interest to that of a relevant deuterium-labeled internal started was used to construct the standard curves. Table S4 reports on IS-LM pairings. When constructing the standard curves for mediators where synthetic standards were available, we prepared a 10-point calibration curve. When preparing the concentration range for these standard curves, we took into consideration the biological levels of these mediators, potential difference in the sensitivity and noise of different transitions, and ESI efficiency for the various analytes. Therefore, the number of calibration points was not consistent between different MRM transitions; nonetheless, we ensured that for each MRM transition evaluated there was a minimum of 6 calibration points, with each calibration point analyzed in 5 replicates on three independent occasions. Table S5 reports the range together with the calibration points used for constructing standard curves for each transition, and Table S6 reports the mean values obtained for the *r*^2^ coefficients, slope, and intercept obtained for each transition. We also employed these parameters to calculate the LLOQ and LOD values for each transition as previously described.^21^ Here, a standard curve in methanol:water (50:50) and the influence of the matrix were evaluated by extracting surrogate plasma matrix, which was then spiked post-SPE to prepare a surrogate–matrix-containing standard curve. The results for these parameters in the matrix and solvent are reported in Table S6. In these experiments, we observed that the majority of transitions evaluated in both sets of standard curves gave *r*^2^ values of ≥0.98.

Assessment of carry over of the signal in blank samples injected after the highest point in the standard curve demonstrated no discernible peak corresponding with the retention time of the mediators and internal standards of interest for all of the transitions evaluated.

### Matrix Effects

**Figure 2:**
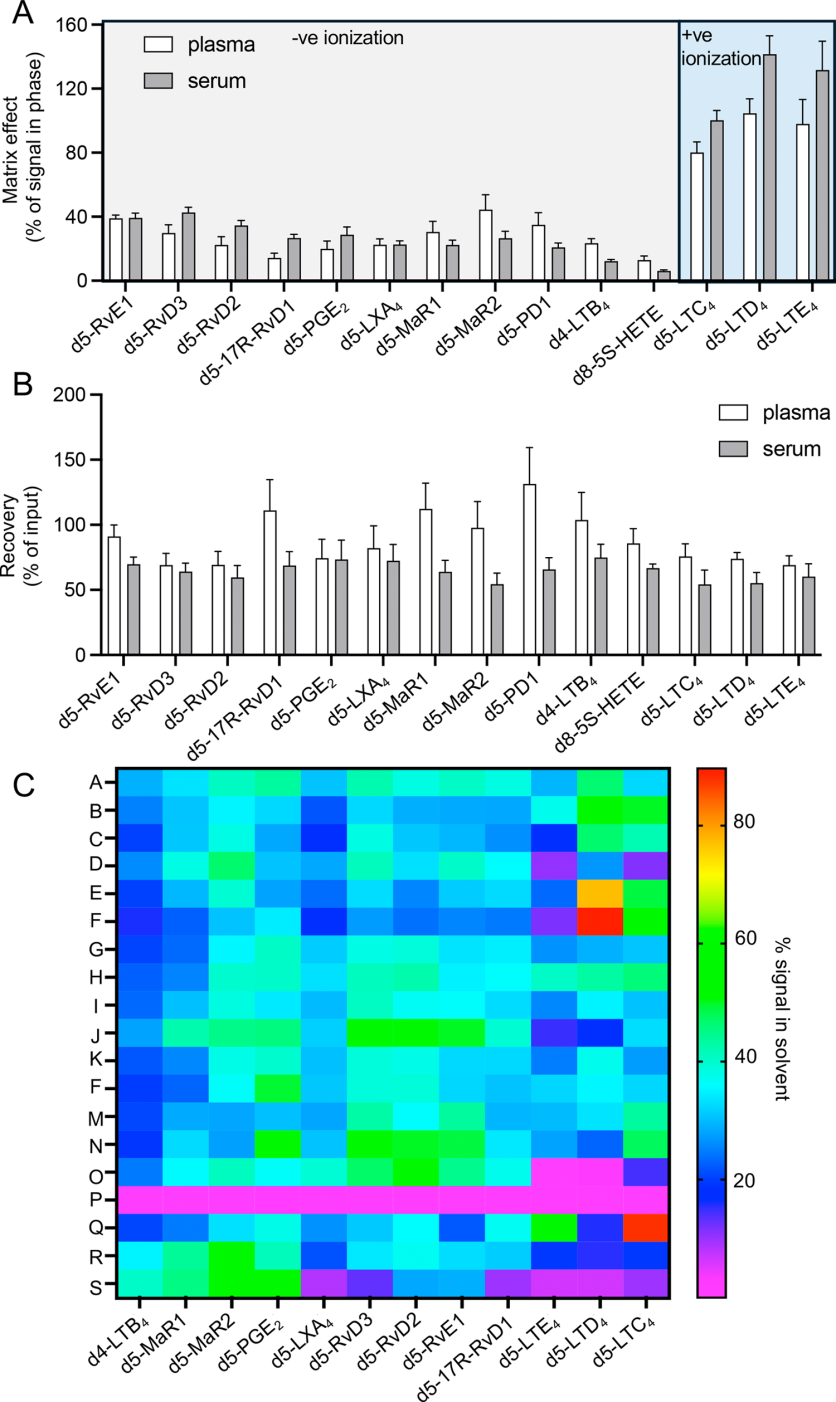
Evaluation of the matrix effects, extraction recovery, use of different extraction recovery matrices, and methodologies. (A) Plasma or serum was placed in 4 volumes of methanol, and matrix was extracted using C18 SPE. Matrix effects were evaluated as a peak area ratio of matrix-spiked post-SPE vs matrix-free standard in solvent. (B) Recovery was instead evaluated as a peak area ratio of matrix-spiked pre-SPE vs post-SPE. (C) Plasma was used to evaluate the extraction recovery matrices and methodologies. Analysis was performed in a minimum of a triplicate for each methodology. Details for the methedologies employed can be found in Table S7.

We next evaluated the matrix effects in plasma and serum for these mediators using the deuterium-labeled internal standards. Here, we observed a significant matrix effect for all of the standards evaluated. The results from this analysis demonstrated an increased ion suppression in negative mode for dihydroxylated mediators that elute in the latter part of the chromatographic region when compared to the more polar trihydroxylated mediators. Intriguingly, we observed an ion enhancement effect of the matrix, especially in serum, for those mediators that were evaluated in positive ionization mode (Figure 2A).

### Evaluation of Lipid Mediator Extraction Methodologies

C18 SPE is widely used to extract lipid mediators from biological matrices. Thus, we next sought to determine the matrix effects and recoveries for deuterium-labeled lipid mediators using human plasma and serum as the biological matrices of interest. Evaluation of deuterium-labeled lipid mediator recoveries demonstrated that we obtained ∼78% recovery for the 14 internal standards evaluated (Figure 2B).

Having observed a marked ion suppression for a subset of mediators, we next sought to explore whether other methodologies and extraction matrices may provide better removal of the matrix and improve the signal of the mediators of interest. For this purpose, we explored a range of methodologies and systems as reported in Table S7 and Figure 2C. Notably, we did not observe marked improvements in the signal obtained for the methods evaluated when compared to C18 SPE (Condition A; Figure 2C).

**Figure 3:**
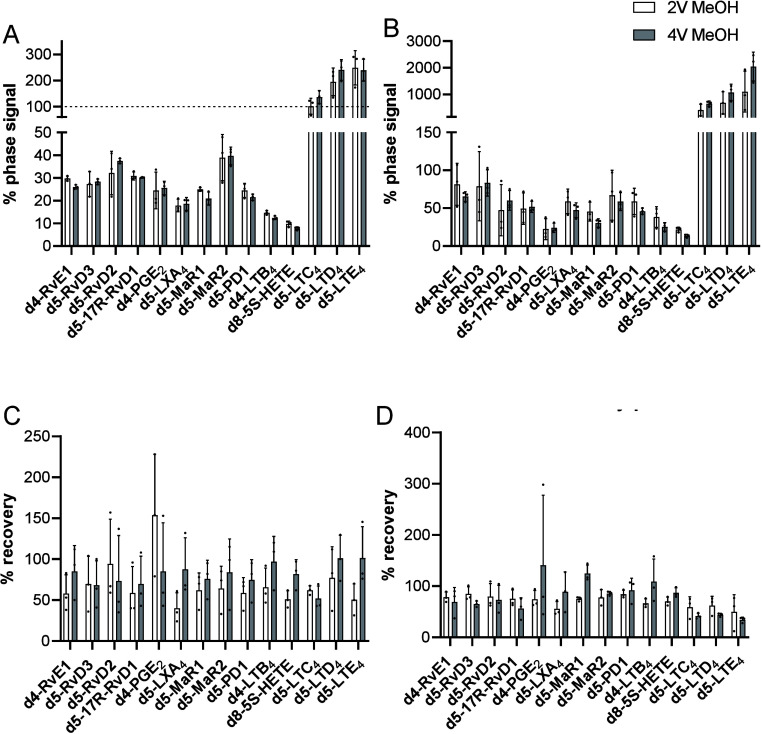
Evaluation of the influence of the solvent volume used for protein precipitation on ion suppression and lipid mediator recovery. Serum (A, C) or plasma (B, D) was placed in 2 or 4 volumes of methanol containing deuterium-labeled internal standards. These were kept at −20 °C for 45 min and then subjected to solid-phase extraction. (A, B) Matrix effect was calculated as a function of the signal obtained for each of the internal standards when injected in methanol. (C, D) Recovery was calculated as a function of the internal standard signal obtained for these molecules when spiked into the matrix after extraction. Results are from *n* = 3 samples.

We next evaluated whether altering the volumes of methanol used to precipitate proteins may have an influence on both the matrix effect and the extraction efficiency. For this purpose, we compared recoveries obtained when using 2 volumes of methanol to those obtained when using 4 volumes of methanol for protein precipitation in serum. The results from this analysis demonstrated that the two methodologies gave comparable results (Figure 3).

**Figure 4:**
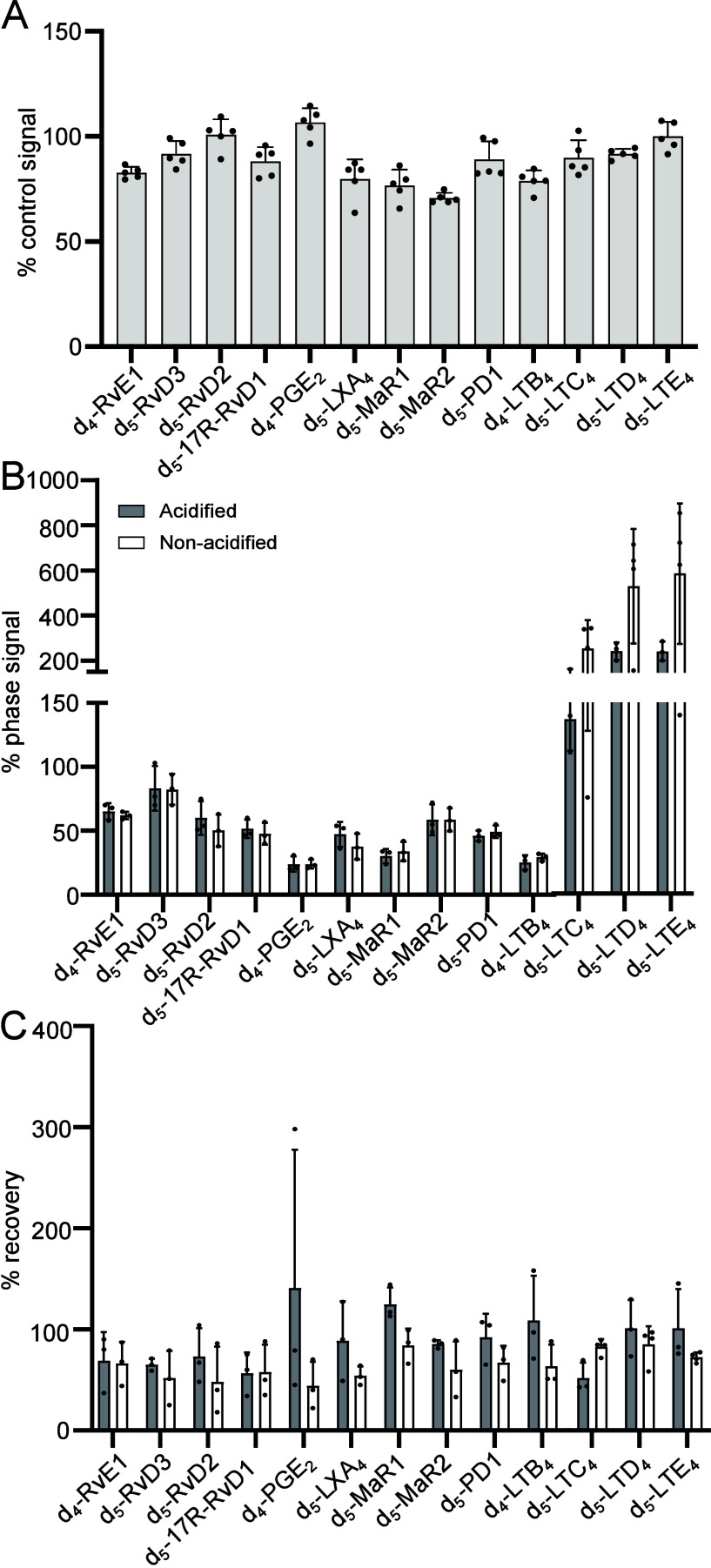
Evaluation of the influence of sample acidification on ion suppression and lipid mediator recovery. (A) To estimate loss during the evaporation stage, deuterium standards were placed in methyl formate or methanol and placed under a gentle stream of nitrogen. Recovered amounts for each standard were compared with amounts found in the reference sample. Results are from *n* = 5 samples. (B, C) Serum was placed in 4 volumes of methanol containing deuterium-labeled internal standards. These were kept at −20 °C for 45 min and then subjected to solid-phase extraction. Prior to lipid mediator extractions, 9 mL of pH 7 (nonacidified) or pH 3.5 water was added. (B) The matrix effect was calculated as a function of the signal obtained for each of the internal standards when injected in methanol. (C) Recovery was calculated as a function of the internal standard signal obtained for these molecules when spiked into the matrix after extraction. Results are from *n* = 3 samples.

To address the potential sample loss due to solvent evaporation after the C18 SPE, we evaluated the extent of signal loss during sample preparation. We compared the signals of deuterium-labeled internal standards in methyl formate and methanol, both subjected to the evaporation step, with those of internal standards that did not undergo this step. Our findings indicate that while some signal loss occurs during this step, it is generally less than 20% for most mediators (Figure 4A).

Recent studies suggest that sample acidification may reduce lipid mediator recovery, possibly due to greater hydrophobic interaction with the plastic of the SPE cartridge and out of bulk solution matrix carry over enhancing ion suppression.^28^ Therefore, we evaluated whether this was also observable when quantifying SPM. In these experiments, we compared ion suppression and recovery by monitoring the signal obtained for deuterium-labeled internal standards in plasma following C18 extraction. Here, we observed that both ion suppression and recovery were comparable in samples that were acidified to those that were not acidified prior to loading on the C18 columns (Figure 4B and 4C).

## Method Validation

We next assessed the accuracy and precision of the analytical method. For this purpose, we first evaluated the inter- and intraday precision and accuracy at three concentrations of the molecules (low, intermediate, and high) in phase, an approach used to determine these parameters when analyte-free matrices are not available.^20^ Assessment of intraday precision for the full method gave RSDs of 12%, 6%, and 5% at the low, intermediate, and high concentrations tested (Table S8). Interday precision gave RSD values of 11%, 6%, and 5% in phase at low, intermediate, and high concentrations (Table S8).

The accuracy of the method was evaluated next. Using the standard curve prepared in phase, we observed that the overall accuracy for the transitions was 94% at low concentration and 89% and 88% at medium and high concentrations (Table S9). The interday accuracy for all of the transitions evaluated was of 95%, 85%, and 87% at the low, medium, and high concentrations evaluated. When we determined the accuracy values in a subset of the transitions, specifically evaluating one transition for each of the compounds evaluated, we found the intraday accuracy values were 120%, 107%, and 111% while the interday accuracy values were 150%, 107% and 108% at the low, intermediate, and high concentrations, respectively (Table S9).

We next assessed the inter- and intraday precision and accuracy of the entire method by spiking an artificial matrix with the three concentrations of standards employed above and subjecting these to SPE extraction prior to LC-MS/MS analysis. In these studies, we observed that the intraday RSD precision values were within the acceptable ranges, namely, 19% at low concentration and 14% and 13% at intermediate and high concentrations, and the interday RSD values were 16%, 12%, and 8% at each of these three concentrations, respectively (Table S10).

To evaluate the accuracy, we used the two sets of standard curves that we previously prepared (i.e., the standard curve prepared in matrix and that prepared in phase). Using the standard curve prepared in phase, we observed that the overall intraday accuracy values for the transitions evaluated were 123%, 87%, and 84% at the low, intermediate, and high concentrations evaluated (Table S11), while the interday accuracy values were 140%, 87%, and 86% for the three concentrations, respectively (Table S11). When we used the standard curves prepared in matrix, we observed that the overall intraday accuracy values were 121%, 105%, and 105% at the low, intermediate, and high concentrations evaluated, while the interday accuracy values were 148%, 100%, and 100% at these concentrations, respectively (Table S12). Evaluation of a subset of transitions for each of the mediators demonstrated that the standard curve in phase gave better inter- and intraday accuracies at the lowest concentrations tested with the overall intra- and interday accuracy for the standard curve in phase being 121% and 138%, respectively, whereas intra- and interday accuracies for the standard curve in matrix were 125% and 157%, respectively (Table S13).

Together, these results demonstrate that the overall method performs within acceptable accuracy and precision parameters. However, we observed that the accuracy for some transitions was low, likely because only a limited range of deuterium-labeled internal standards are available, leading to the use of surrogate internal standards for many molecules. Despite this, the high level of instrument and method precision observed indicates that relative changes in the levels of these molecules can be reliably studied. This is particularly relevant for applications in preclinical studies and nondiagnostic clinical evaluations, where relative changes are more significant than absolute accuracy.

To further evaluate the robustness of the methodologies, we obtained human serum. And after extraction, samples were resuspended in phase and then diluted with phase to either 1:2 or 1:3. Lipid mediators were quantified in the undiluted and diluted samples. Here, we observed that while the RSD for the quantified concentrations of some of the molecules, e.g., TXB_2_, PGE_2_, 17R-PD1, and PDX, was above 15%, the overall RSD values for the precision for the identified mediators were within the acceptable range (i.e., less than 15%; Table S14).

### Lipid Mediator Stability

We next sought to evaluate both the short-term and the long-term stability of the lipid mediators in the samples. For this purpose, we explored the stability of these molecules in extracted plasma matrix, as this is one of the more tractable biosamples for the development of diagnostics for patient stratification due to ease of access. To enable the evaluation of the full range of lipid mediators, we spiked the plasma with a mixture of synthetic standards and evaluated their stability at 4 °C, a temperature that is typically used for storage of samples in autosamplers. Evaluation of the differences in the concentrations of 28 lipid mediators demonstrated that overall these mediators displayed good short-term (up to 3 days) and long-term (up to 21 days) stability. In short-term experiments, only the concentration of RvD1_*n*–3 DPA_ was found to be more than 25% different from the baseline calculation. By day 3, the number of mediators with calculated changes in concentrations > 25% to those calculated on day 0 increased to 4 of the 29 evaluated and included PGD_2_, PGE_2_, RvD4, and PDX. At day 14, the number of mediators that gave concentrations that were >25% different from those calculated at the baseline increased to 7 and included RvT1, PD1_*n*–3 DPA_, RvE4, LXA_4_, PGF_2a_, and TXB_2_, with values remaining relatively similar at day 21 (Table S15). One point of note is that we observed lower variability in calculated mediator concentrations in the longer term samples. This observation likely arises from technical variability as these experiments were performed at distinct intervals. These results suggest that under the evaluated conditions, most lipid mediators of interest display reasonable stability.

**Figure 5:**
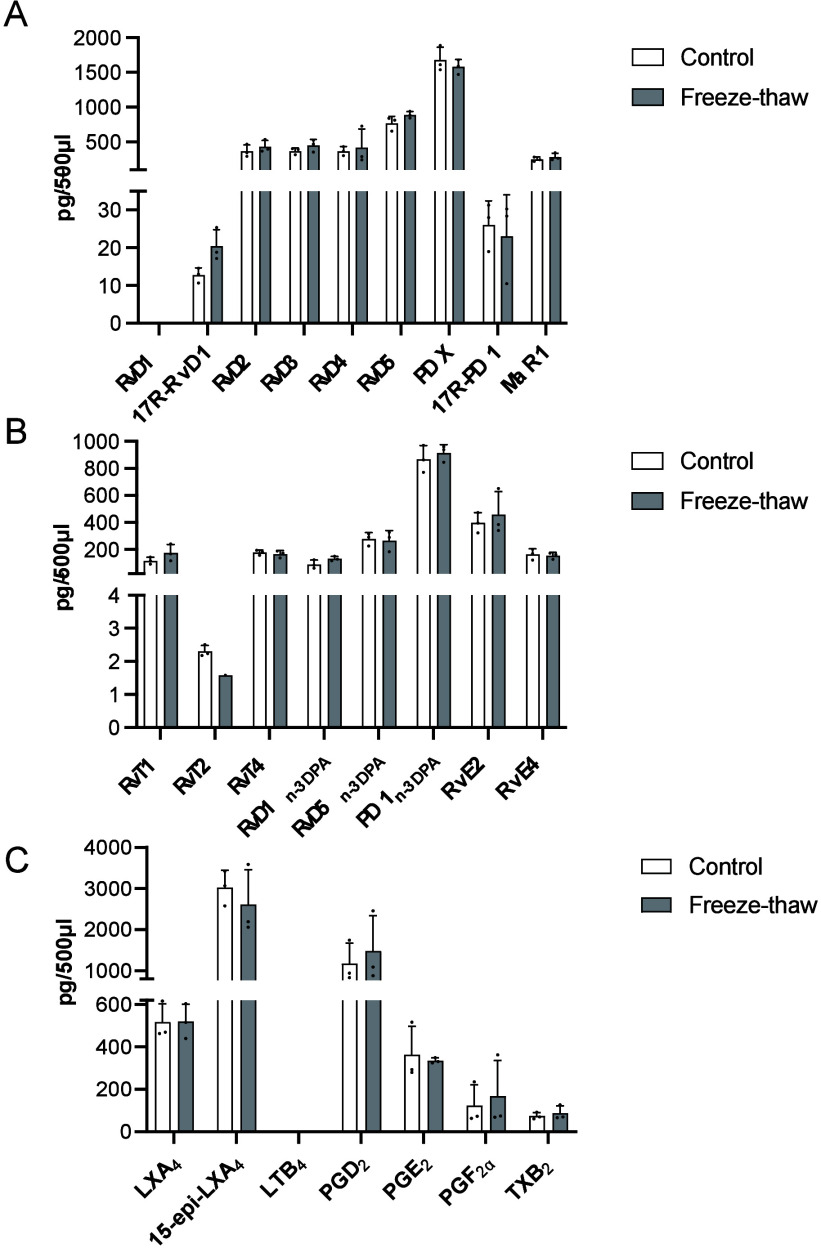
Lipid mediator concentrations remain essentially unchanged after one freeze–thaw cycle. Commercial human serum was thawed on ice; aliquots were taken (Control) and placed in four volumes of ice-cold methanol containing deuterium-labeled internal standards. The serum was refrozen and stored at −80 °C for 24 h. This was then thawed on ice, and aliquots were placed in four volumes of ice-cold methanol containing deuterium-labeled internal standards. Lipid mediators were extracted and quantified using LC-MS/MS. (A) DHA-, (B) *n* – 3 DPA- and EPA-, and (C) AA-derived lipid mediators. Results are from *n* = 3 replicates from two experiments.

We next sought to evaluate the influence of freeze–thawing on the integrity of these molecules. Using commercial serum, we compared the concentrations of the identified mediators in samples that underwent one freeze–thaw cycle with those that did not. The results from this analysis demonstrated that the concentrations in the two conditions were similar, suggesting that one freeze–thaw cycle did not influence the mediator integrity in these samples (Figure 5). These results further underscore the overall stability of these mediators in biological matrices.

### Evaluation of Integration and Signal-to-Noise Methodologies

We next sought to compare different approaches used for determining the signal-to-noise ratios. Here, we focused on the algorithms found on Sciex OS, namely, the peak to peak, standard deviation, and relative noise algorithms (see Methods for further details). In our initial analysis, we calculated the signal-to-noise ratios using the approach proposed by the US Pharmacopeia and adopted by the European Medicines Agency,^25,29^ whereby the noise value is obtained by integrating the region in the chromatogram corresponding to the peak of interest in a matrix blank sample. To replicate the sample conditions as much as possible for these experiments, we used artificial plasma and spiked the mediators of interest. Furthermore, since fluctuations in the calculations of the background signal will have a greater influence in the signal-to-noise calculation of low-abundance molecules, for these determinations, we spiked the matrix with the low concentration of mediators used for determining the precision and accuracy of quantitation in the experiments described earlier. For these experiments, we used the AutoPeak algorithm to integrate the data.

Here, we observed that while the US Pharmacopeia, standard deviation, and relative noise approaches provided comparable results to those obtained using the separate matrix blank, the signal-to-noise ratios obtained using the peak-to-peak algorithm were markedly lower across most of the transitions evaluated (Table S16 and Figure S1).

We next tested whether the integration algorithms yield distinct results when calculating the signal-to-noise ratios. For this purpose, we compared the results obtained using the AutoPeak algorithm with those using the MQ4 algorithm available in Sciex OS. The results from this analysis suggest that overall differences between the two integration algorithms are small, especially when evaluating peaks with signal-to-noise ratios ≤ 8 for all three signal-to-noise methodologies tested (Table S17).

Complex matrices tend to influence ionization, leading to amplified fluctuations in the background and peak signals. One approach to obviate for these matrix-induced fluctuations and facilitate both the identification and the integration of the peak of interest is to employ smoothing algorithms. While this solution is a default feature in some software, it is not universally applied across all of the software used in the analysis of lipid mediator data sets and has recently been questioned. Therefore, we sought to empirically determine the influence of smoothing on the calculation of the signal-to-noise ratios.

**Figure 6:**
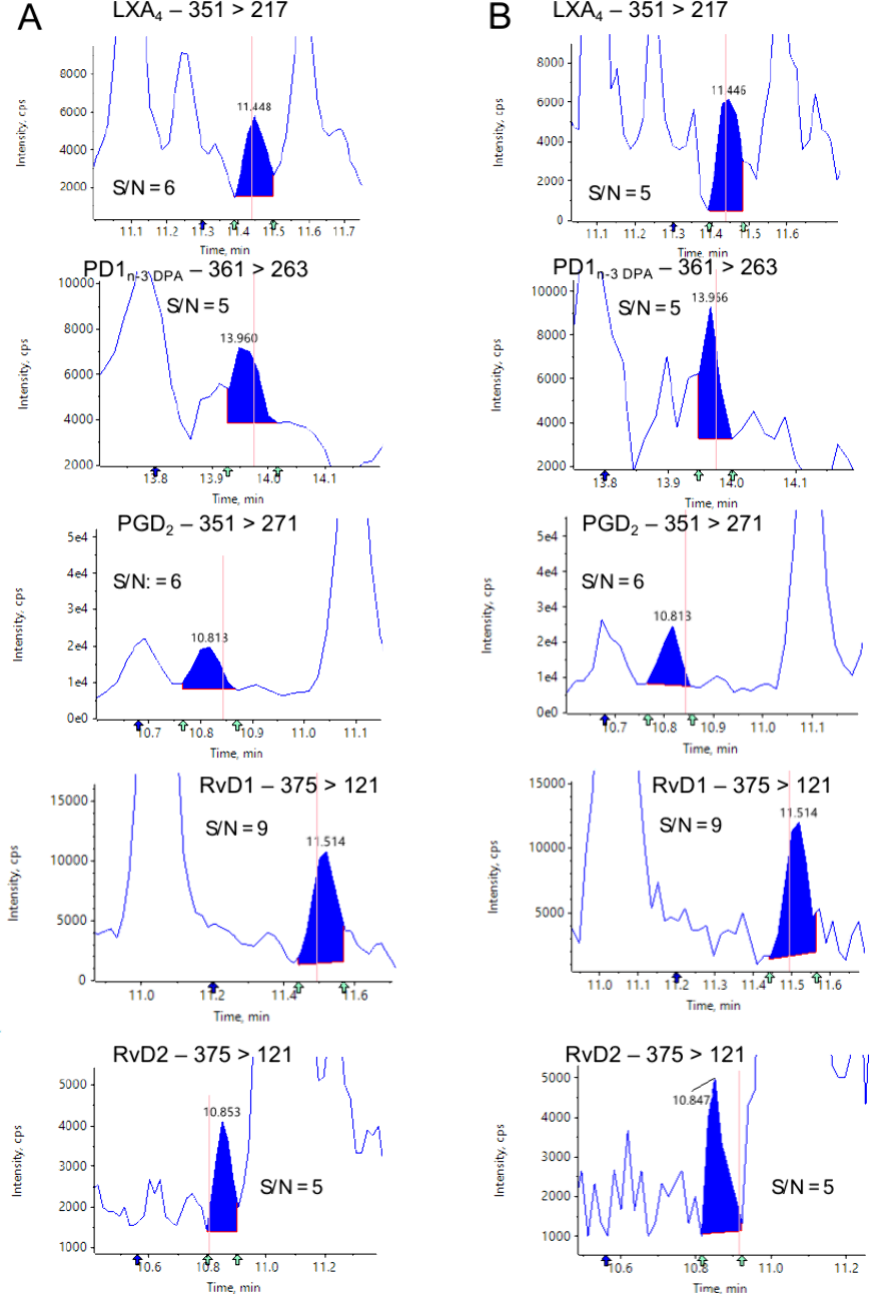
Comparison of peak shape and identification for low-abundance signals between smoothed and unsmoothed MRM chromatograms. Representative MRM traces for (A) smoothed and (B) unsmoothed chromatograms for low-abundance signals (i.e., *s*/*n* < 8).

For this comparison, we evaluated the results obtained using the relative noise algorithm as this was the least subjective approach to calculating the signal-to-noise ratios since the algorithm determines the noise threshold across the entire chromatogram rather than in a user-defined region. We also used the lowest smoothing setting available in Sciex OS for this analysis, whereby for data integrated using the AutoPeak algorithm we use the “low” smoothing function and for data integrated using the MQ4 algorithm we used a Gaussian smooth value of 1. We then explored whether there were differences between the results obtained using either of these algorithms and those obtained using unsmoothed data. Side by side comparisons of the signal-to-noise ratios gave comparable signal-to-noise ratios when evaluating low-abundance peaks (s/n < 8) with differences becoming more pronounced in peaks with s/n > 20 when using either the AutoPeak or the MQ4 algorithm (Table S18 and Figure 6). Indeed, when we averaged the differences observed in the results obtained for the signal-to-noise values, we observed that for all of the transitions evaluated, the average difference was 4 for results obtained with the AutoPeak algorithm and of 5 for results obtained with the MQ4 algorithm. This difference was markedly lower when we evaluated only signals with signal-to-noise values ≤ 8, where we observed that the averaged difference was 2 for results obtained with the AutoPeak algorithm and 3 for results obtained with the MQ4 algorithm. Importantly, out of the 49 transitions evaluated with s/n ≤ 8 only, 1 gave s/n values below the LLOQ cutoff (i.e., s/n = 5) when evaluated without smoothing and >5 when quantified after smoothing. Moreover, we did not observe any indication that the low smoothing parameter could generate peaks from noise signals. Specifically, no peaks were found in the smoothed data set that were absent in the unsmoothed data set (Table S18). These results suggest that while smoothing may influence the calculated signal-to-noise values, this effect is primarily relevant at higher signal-to-noise ratios. Therefore, it has a limited influence on whether a specific peak meets the threshold for identification or quantitation criteria.

### Identification of Lipid Mediators in Standard Reference Material

Having established the methodologies, we next sought to determine the SPM and eicosanoid levels in a commercially available standard reference material that can be used to benchmark methodologies across different laboratories and platforms. As serum concentrations of SPM and eicosanoids are higher than those found in plasma and in order to increase the utility of these measurements across different platforms that tend to vary in sensitivities, we opted to validate our method using NIST SRM-909c, a serum standard reference material. In these experiments, we also explored whether changes in volumes influence the coverage and accuracy of the method using either 500 or 100 μL volumes. The results from these studies demonstrate that the number of lipid mediators identified between the two groups was identical and that the within group precision values for the replicates were within an acceptable range (i.e., RSD < 15%) with results obtained from the 100 μL volume appearing to yield a lower degree of variability in the replicates. Comparing the quantities calculated for each of the lipid mediators between the two groups indicated that the overall precision of the quantitation for the method was within an acceptable range with an RSD of 10.8% (Table 1).

**Table 1:** NIST SRM-909c Lipid Mediator Profiles^a^

SRM serum		Q3		mean concentration (pg)			precision	
analyte	Q1	primary	secondary	500 μL	100 μL	% change/mL	500 μL	100 μL
DHA-derived mediators								
RvD1	375	121	–	*	*	*	*	*
17R-RvD1	375	233	141	176	27	–0.20%	4.20%	10.50%
RvD2	375	175	215	661	143	7.80%	11.20%	7.70%
RvD3	375	147	137	590	114	3.45%	9.05%	10.97%
RvD4	375	101	131	1156	293	26.80%	26.70%	15.30%
RvD5	359	199	141	17 900	3575	–0.10%	4.70%	2.00%
PD1	359	123	–	*	*	*	*	*
PDX	359	153	137	31 175	6438	3.20%	5.80%	4.70%
17R-PD1	359	153	–	*	*	*	*	*
PCTR1	650	231	–	*	*	*	*	*
PCTR2	521	231	–	*	*	*	*	*
PCTR3	464	231	–	*	*	*	*	*
MaR1	359	177	–	*	*	*	*	*
MaR2	359	221	–	*	*	*	*	*
MCTR1	650	191	–	*	*	*	*	*
MCTR2	521	191	–	*	*	*	*	*
MCTR3	464	191	–	*	*	*	*	*
*n*– 3 DPA-derived mediators								
RvT1	377	211	143	951	186	–2.00%	23.60%	10.50%
RvT2	377	227	–	*	*	*	*	*
RvT4	361	193	211	4573	832	–9.00%	5.20%	10.40%
RvD1_*n*–3DPA_	377	143	215	684	133	2.86%	11.60%	9.08%
RvD5_*n*–3DPA_	361	199	143	6955	1358	–2.40%	8.60%	5.70%
PD1_*n*–3DPA_	361	263	183	13 075	2725	4.20%	7.50%	7.50%
EPA-derived mediators								
RvE1	349	161	–	*	*	*	*	*
RvE2	333	115	253	5548	1395	25.70%	23.50%	10.20%
RvE4	333	253	115	515	258	60.10%	29.20%	15.70%
AA-derived mediators								
LXA_4_	351	235	115	1550	370	19.40%	15.30%	8.00%
LXB_4_	351	221	163	1917	480	20.20%	24.30%	12.60%
15-epi-LXA_4_	351	115	217	8458	1953	15.40%	9.50%	19.60%
LTB_4_	335	195	–	*	*	*	*	*
LTC_4_	626	189	–	*	*	*	*	*
LTD_4_	497	189	–	*	*	*	*	*
LTE_4_	440	189	301	32	7	13.40%	14.60%	17.00%
PGD_2_	351	189	233	3787	791	4.40%	28.20%	7.10%
PGE_2_	351	189	175	422	72	–15.00%	9.60%	11.40%
PGF_2*α*_	353	171	247	1930	408	5.80%	27.50%	16.20%
TXB_2_	369	169	195	1186	271	14.10%	28.90%	29.80%

a Lipid mediators were extracted and profiled using LC-MS/MS-based methodologies. They were identified by matching the retention time of the quantifier and qualifier ion pairs denoted above with those from reference standards (see Methods for further details). Results are from *n* = 4 determinations per volume of serum used. Asterisk (*) designates below the lower limits of quantitation.

Using this data set, we also sought to validate the utility of the relative noise algorithm in calculating the signal-to-noise ratios by comparing the results obtained with this algorithm to those obtained with the approach recommended by the US Pharmacopeia. Here, we observed that in many of the instances it was not possible to identify a true baseline immediately adjacent to the peak of interest, as there were closely eluting isomeric peaks. This led to signal-to-noise values that were much lower than those calculated with the relative noise approach (Figure S2). To try to overcome this challenge, we sought to identify a region within the chromatogram that was as close as possible to the peak of interest and that was devoid of isomeric peaks and thus represented a true baseline (Figure S2). In chromatograms where we were able to identify such a baseline, e.g., for PDX and RvT4, the signal-to-noise values obtained were similar to those obtained using the relative noise approach. We also observed that even in instances where small isomeric peaks were included in the noise calculation, e.g., RvE2, PGD_2_, and PGE_2_, the signal-to-noise ratios obtained were comparable to those obtained using the relative noise algorithm. These findings confirm the relative noise approach in calculating the signal-to-noise values.

To further evaluate the utility of our methodology, we also measured lipid mediator levels in plasma from healthy volunteers. Here, we observed that while a number of SPMs, including PDX and RvE1, were present, in most samples analyzed others, including RvT4, were only identified in a subset of samples (Table S19). In these plasma samples, we also confirmed the utility of the relative noise approach as we observed that this gave results similar to those obtained with the US Pharmacopeia approach when using a baseline signal devoid of isomeric peaks within the chromatogram (Figure S3).

## Conclusion

In the present study, we established and validated methodologies for the extraction, identification, and quantification of specialized pro-resolving mediators in biological systems. We also evaluated the levels of these molecules in NIST SRM-909c, a serum standard reference material, which can be used to validate methodologies for the identification and quantitation of these molecules on other platforms. As many of the lipid mediators have both double-bond and chiral isomers that differ in both their biosynthetic pathways and their biological activators, it is essential that methodologies developed to identify and quantify these molecules can distinguish between these isomers. We demonstrate that the chromatographic method employed robustly separates double-bond isomers and chiral isomers of the different mediators. This coupled with the use of two transitions in the identification of each of the mediators enables the unambiguous identification of the mediators of interest. We also noted that the concentrations of the mediators identified in this SRM and in commercial serum were within the bioactive concentrations of these molecules. As coagulation is a key step in tissue repair and in line with observations made by others,^30,31^ this supports a potential role for these mediators in regulating clot remodeling and tissue repair.

One limitation of our methodology is in the accuracy of the quantitation; this limitation is one that is widely acknowledged in the field of lipidomics and likely derives from the limited availability of internal standards to accurately account for losses and matrix effects for each of these molecules. Instead, the current methodology makes use of surrogate internal standards to estimate the recovery of structurally related mediators. As the precision of the methodology was within acceptable parameters, the methodology developed herein will still be useful in understanding the relative regulation of lipid mediators between different experimental conditions, as any differences in quantitation will be reproduced across all experimental groups.

Signal-to-noise ratios are crucial for identifying and quantifying lipid mediators. Various commercial programs utilize different algorithms to integrate raw data from mass spectrometers and calculate SNRs. Furthermore, there are different approaches that can be used for the calculation of the noise signal:noise ratios that rely on different algorithms and approaches to estimate the noise signal. The impact of these different approaches on the identification of SPMs and classic eicosanoids has not been previously explored.

In our experiments, using a surrogate plasma matrix spiked with small amounts of lipid mediators, we found that using an external blank devoid of isomeric peaks for the mediators of interest yielded results comparable to methodologies recommended by the US Pharmacopeia. Specifically, the relative noise algorithm and the standard deviation approaches provided similar results to those obtained using the approach recommended by the US Pharmacopeia, while the peak-to-peak approach consistently produced lower signal-to-noise ratios.

When repeating this analysis in either plasma or serum, strict adherence to US Pharmacopeia methodologies resulted in consistently lower signal-to-noise ratios due to the presence of closely eluting isomeric peaks interfering with the calculation. However, when applying the same equation and noise area recommended by the US Pharmacopeia to a region within the chromatogram that was devoid of isomeric peaks, the values obtained were comparable to those obtained with the relative noise approach.

These findings strongly support the utility of the relative noise approach for calculating the signal-to-noise ratios. This method not only provides results comparable to those obtained using the US Pharmacopeia approach but also eliminates analyst subjectivity, thereby increasing the reproducibility of results across laboratories.

We also observed that the AutoPeak and MQ4 algorithms gave comparable results for each of the signal-to-noise approaches tested. Finally, we found that the use of a “low” degree of smoothing did not result in false positive identification, with signal-to-noise values for low-abundance peaks (i.e., signal-to-noise values < 8) being comparable to those obtained with unsmoothed data.

Taken together, the results presented herein establish a robust approach for the identification of SPM and eicosanoids to help further our understanding of their role in both health and disease.

## Supporting Information Available

Heat map denoting the signal-to-noise ratios calculated using four distinct methodologies; chromatograms together with signal-to-noise ratios for lipid mediators identified in NIST-SRM909c; chromatograms together with signal-to-noise ratios for lipid mediators identified in human plasma; table listing the origin of the lipid mediators employed in our studies and the respective extinction coefficients used to calculate their concentrations; list of MRM transitions and mass spectrometer parameters for each of the mediators evaluated; source parameters; lipid mediator IS pairings used for quantitation; range of standards used for the construction of standard curves for each of the transitions; calibration curve parameters and LLOQ and LOD values; comparison of signal intensity for each of the deuterium-labeled mediators following plasma extraction using different extraction methodologies and matrices; intraday and interday precision for standards in solvent; intraday and interday accuracy for standards in solvent; intra- and interday precision of the method using standards spiked in matrix; intra- and interday accuracy of the method using standard curve prepared in solvent; intra- and interday accuracy of the method using a standard curve prepared in surrogate matrix; summary table displaying the two transitions that gave the overall best precision and accuracy values for each mediator; evaluation of quantitation accuracy following sample dilution; lipid mediator stability in extracted matrix at 4 °C; comparison of signal-to-noise ratios obtained using different algorithms and an external blank; different integration algorithms have little influence on the calculation of signal-to-noise ratios; low smoothing has limited effect on the signal-to-noise calculations especially for low-intensity peaks; lipid mediator levels in human plasma blood (PDF)
