# Supplementary material for: Development and Validation of Methodologies for the Identification of Specialized Pro-Resolving Lipid Mediators and Classic Eicosanoids in Biological Matrices
Source: J Am Soc Mass Spectrom. 2024 Sep 10;35(10):2331–43. doi: 10.1021/jasms.4c00211 (PMC11450820; doi:10.1021/jasms.4c00211)
Supplement: Supplementary file 1 — js4c00211_si_001.pdf [file js4c00211_si_001.pdf]

Supporting Information

Development and Validation of Methodologies for the Identification of Specialized Proresolving Lipid Mediators and Classic Eicosanoids in Biological Matrices.

Matthew Dooley[§,‡], Amitis Saliani[§,‡], and Jesmond Dalli[§,$,*]

AUTHOR ADDRESS

[§] William Harvey Research Institute, Barts and The London Faculty of Medicine and Dentistry, Queen Mary University of London, Charterhouse Square, London, EC1M 6BQ, UK.

[$] Centre for Inflammation and Therapeutic Innovation, Queen Mary University of London, London, E1 4NS, UK.

Corresponding author email: j.dalli@qmul.ac.uk

Supporting Figures

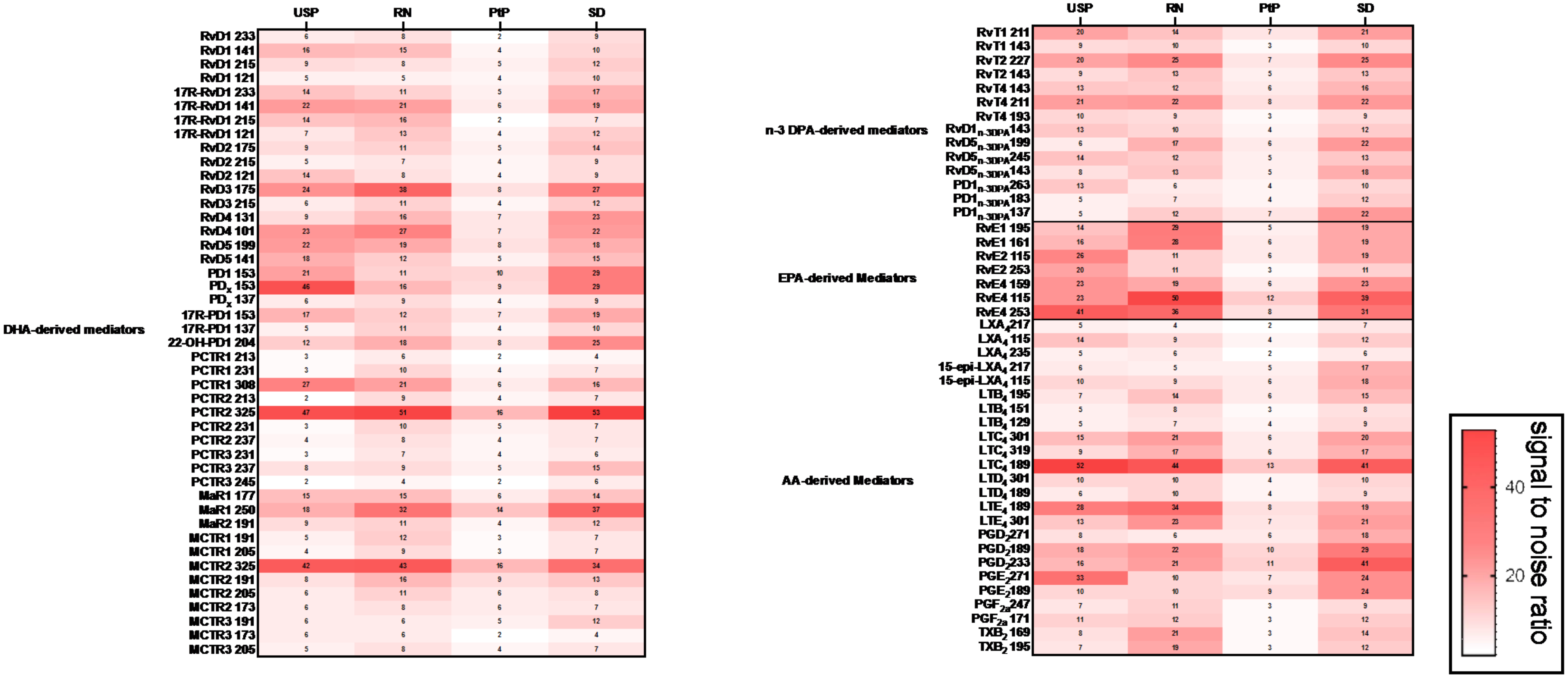

Figure S1: Heat map denoting the signal to noise ratios calculated using four distinct methodologies.

USP = the noise region was identified as the region corresponding to the retention time of the molecule of interest within an external matrix blank.

RN, PtP and SD = the noise region was identified as follows – for the relative noise algorithm this is automatically computed by the algorithm from the MRM trace; for the PtP and SD algorithms this was selected as a region immediately adjacent and of equal width to the peak of interest.

- = No peak detected; RN = Relative noise algorithm; PtP = peak to peak algorithm; SD = standard deviation algorithm.

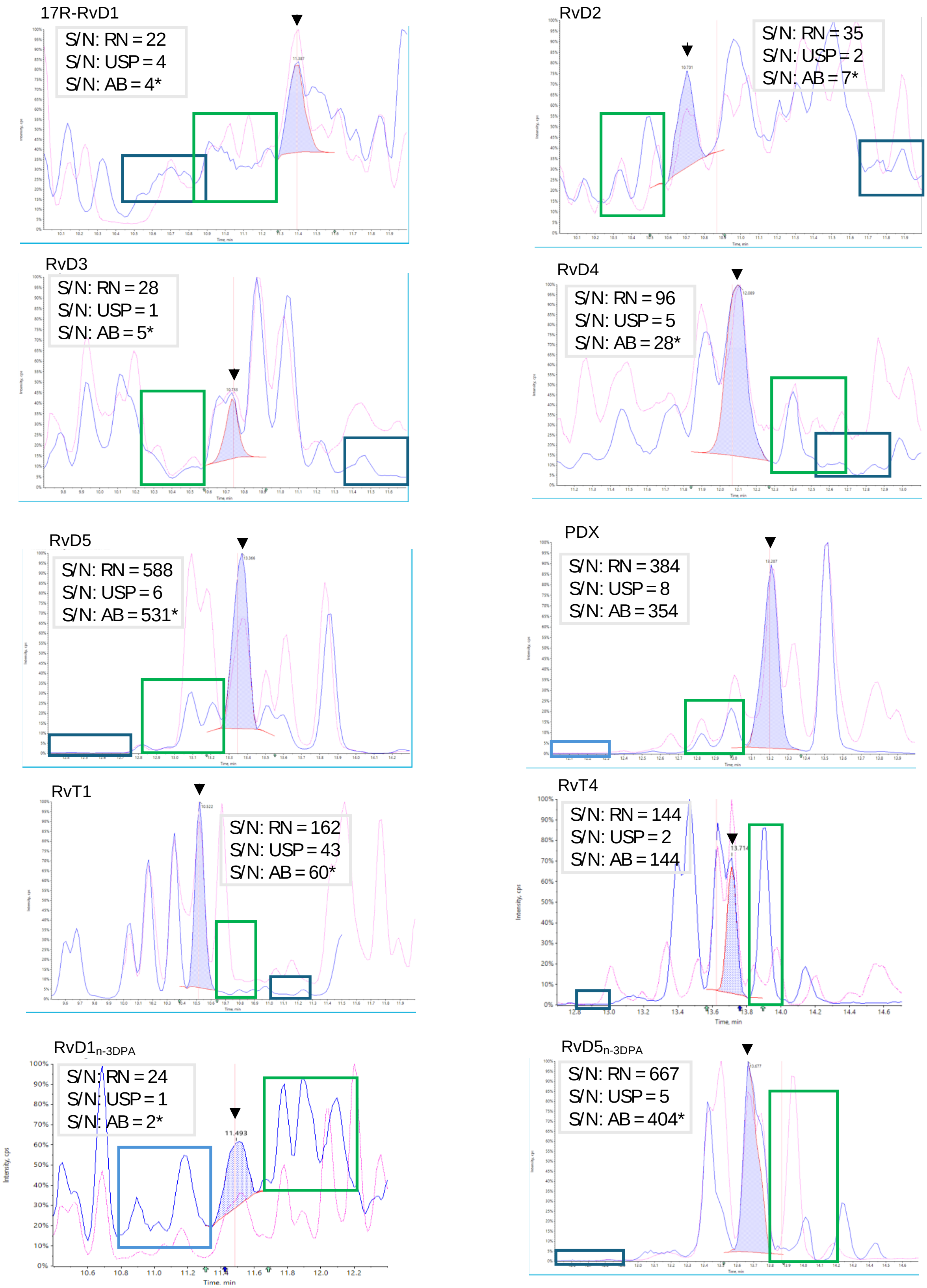
17R-RvD1
S/N: RN = 22
S/N: USP = 4
S/N: AB = 4*
RvD2
S/N: RN = 35
S/N: USP = 2
S/N: AB = 7*
RvD3
S/N: RN = 28
S/N: USP = 1
S/N: AB = 5*
RvD4
S/N: RN = 96
S/N: USP = 5
S/N: AB = 28*
RvD5
S/N: RN = 588
S/N: USP = 6
S/N: AB = 531*
PDX
S/N: RN = 384
S/N: USP = 8
S/N: AB = 354
RvT1
S/N: RN = 162
S/N: USP = 43
S/N: AB = 60*
RvT4
S/N: RN = 144
S/N: USP = 2
S/N: AB = 144
RvD1n-3DPA
S/N: RN = 24
S/N: USP = 1
S/N: AB = 2*
RvD5n-3DPA
S/N: RN = 667
S/N: USP = 5
S/N: AB = 404*

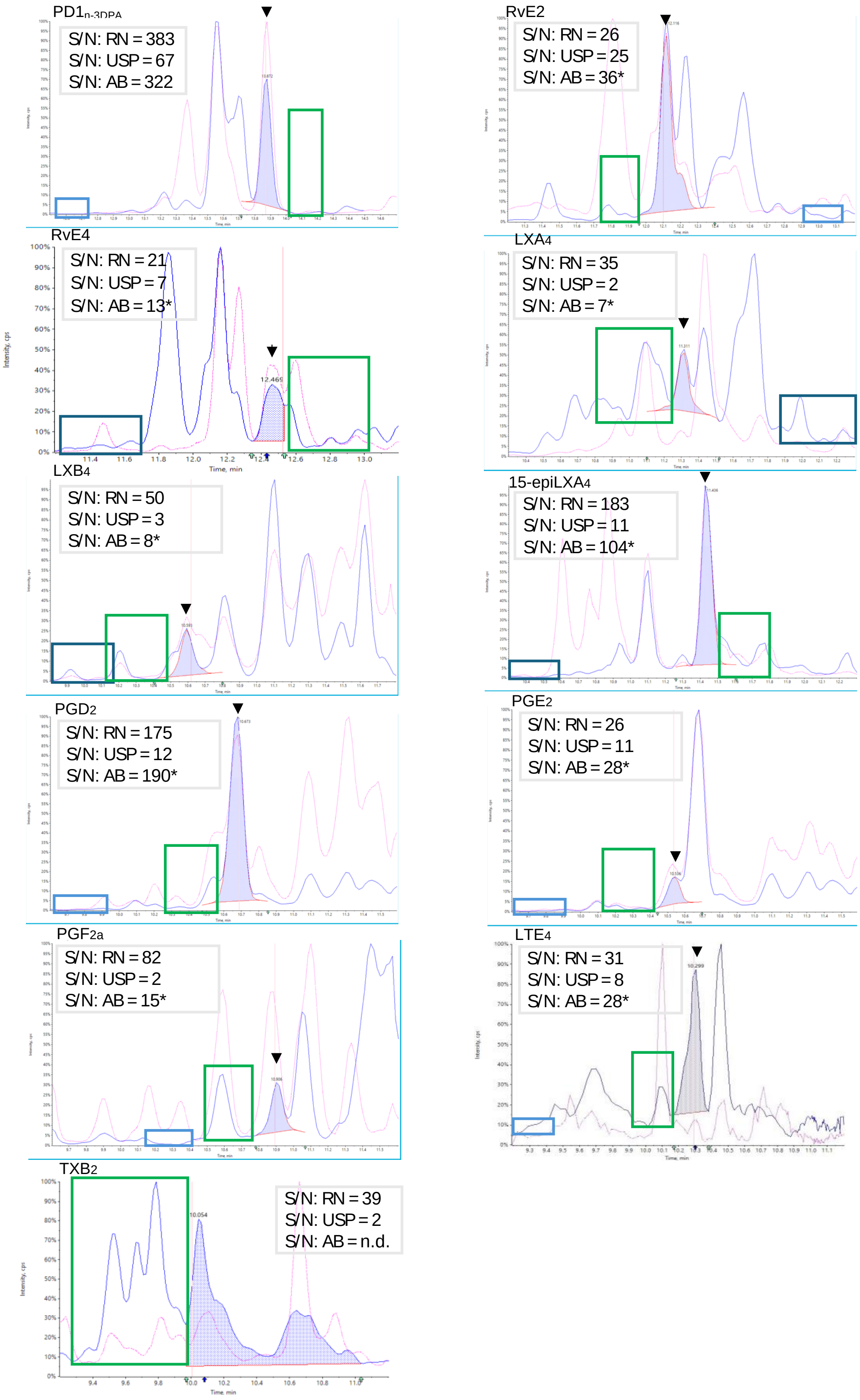
PD1n-3DPA
S/N: RN = 383
S/N: USP = 67
S/N: AB = 322
RvE2
S/N: RN = 26
S/N: USP = 25
S/N: AB = 36*
RvE4
S/N: RN = 21
S/N: USP = 7
S/N: AB = 13*
12.469
Intensity, cps
Time, min
LXA4
S/N: RN = 35
S/N: USP = 2
S/N: AB = 7*
LXB4
S/N: RN = 50
S/N: USP = 3
S/N: AB = 8*
15-epiLXA4
S/N: RN = 183
S/N: USP = 11
S/N: AB = 104*
PGD2
S/N: RN = 175
S/N: USP = 12
S/N: AB = 190*
PGE2
S/N: RN = 26
S/N: USP = 11
S/N: AB = 28*
PGF2a
S/N: RN = 82
S/N: USP = 2
S/N: AB = 15*
LTE4
S/N: RN = 31
S/N: USP = 8
S/N: AB = 28*
10.299
TXB2
S/N: RN = 39
S/N: USP = 2
S/N: AB = n.d.
10.054

Figure S2: Chromatograms together with signal to noise ratios for lipid mediators identified in NIST-SRM909c. Chromatographic traces for primary and secondary transitions employed for the identification of each of the lipid mediators. Signal-to-noise values were calculated using the relative noise algorithm (RN), as recommended by the US Pharmacopeia (USP; green region denotes the 'noise' signal). Alternatively, a region of the same width as the USP recommendation, devoid of or with minimal isomeric peaks, was used, referred to as the adjusted baseline (AB; blue region denotes the 'noise' signal).* = denotes the presence of an isomeric peak within the region used to calculate the noise signal. n.d. = not determined.

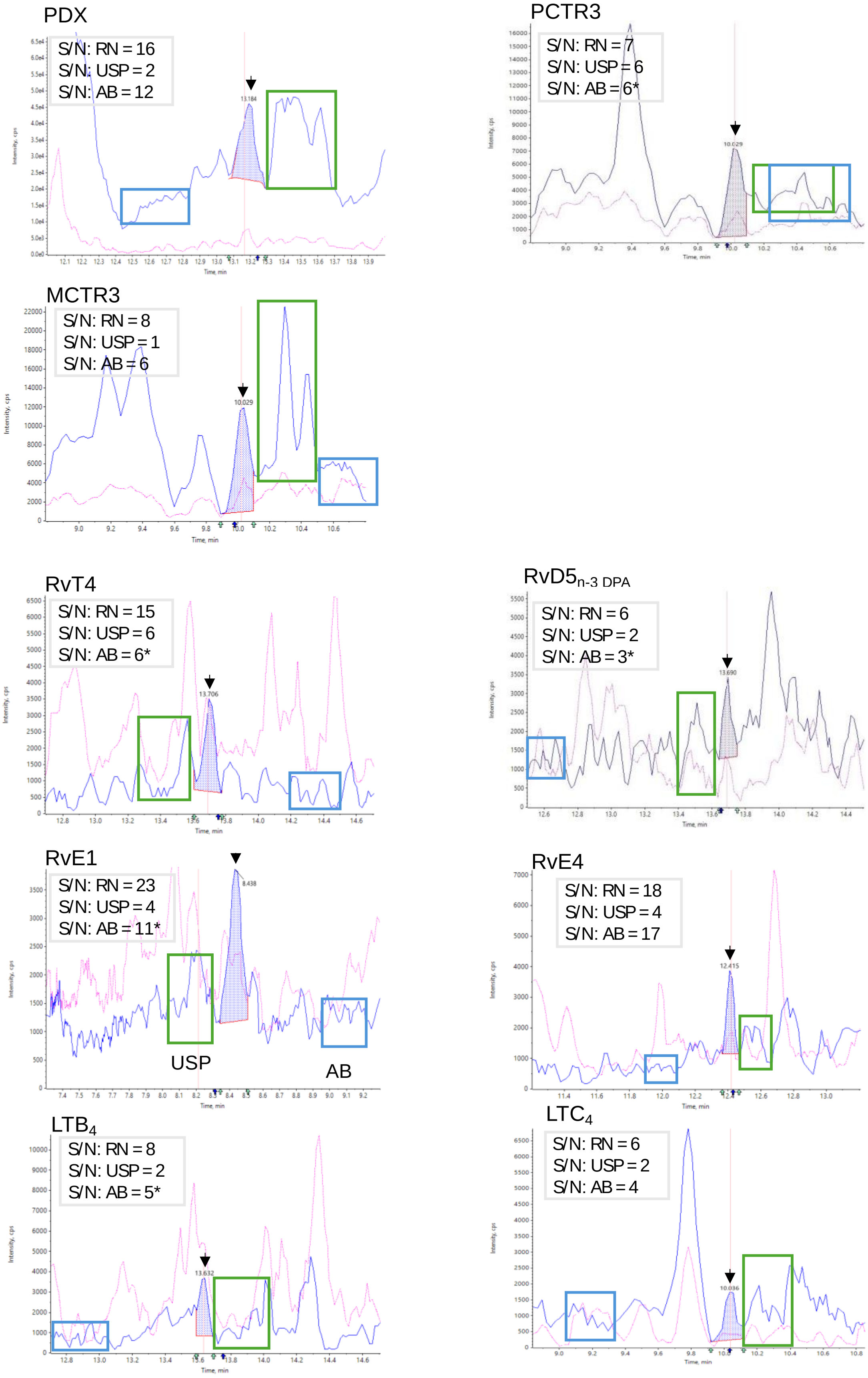
PDX
S/N: RN = 16
S/N: USP = 2
S/N: AB = 12
PCTR3
S/N: RN = 7
S/N: USP = 6
S/N: AB = 6*
MCTR3
S/N: RN = 8
S/N: USP = 1
S/N: AB = 6
RvT4
S/N: RN = 15
S/N: USP = 6
S/N: AB = 6*
RvD5n-3 DPA
S/N: RN = 6
S/N: USP = 2
S/N: AB = 3*
RvE1
S/N: RN = 23
S/N: USP = 4
S/N: AB = 11*
USP
AB
RvE4
S/N: RN = 18
S/N: USP = 4
S/N: AB = 17
LTB4
S/N: RN = 8
S/N: USP = 2
S/N: AB = 5*
LTC4
S/N: RN = 6
S/N: USP = 2
S/N: AB = 4
Time, min
Intensity, cps

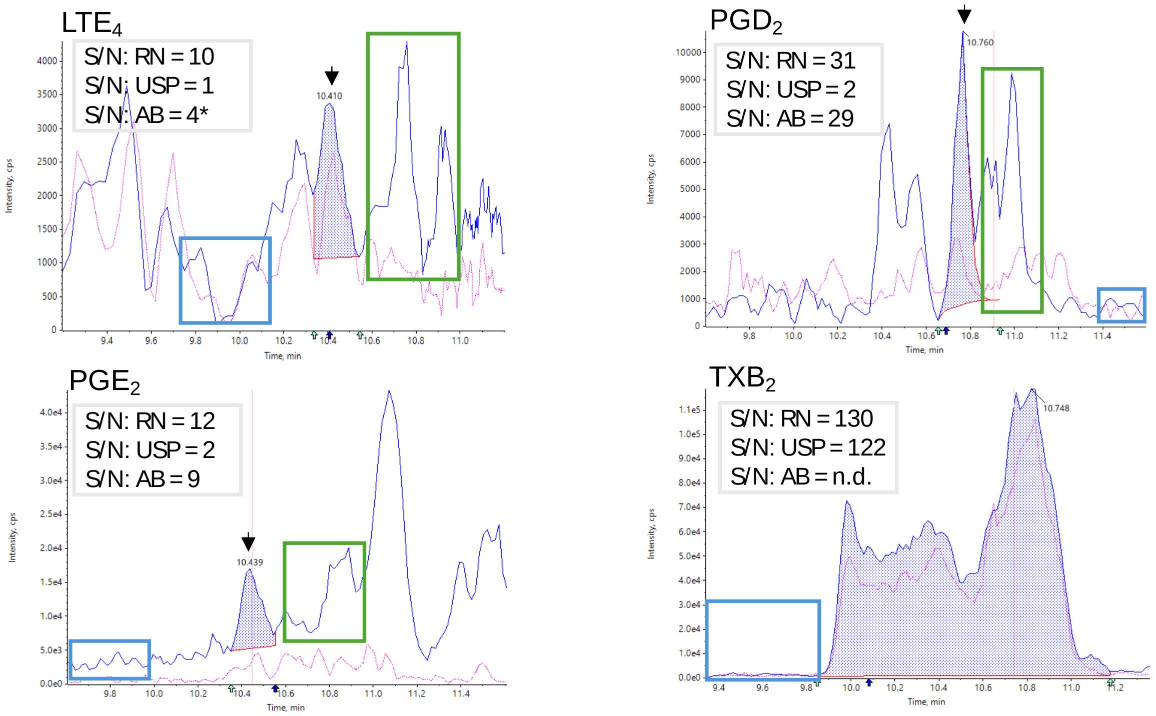

Figure S3: Chromatograms together with signal to noise ratios for lipid mediators identified in human plasma. Chromatographic traces for primary and secondary transitions employed for the identification of each of the lipid mediators. Signal-to-noise values were calculated using the relative noise algorithm (RN), as recommended by the US Pharmacopeia (USP; green region denotes the 'noise' signal). Alternatively, a region of the same width as the USP recommendation, devoid of or with minimal isomeric peaks, was used, referred to as the adjusted baseline (AB; blue region denotes the 'noise' signal). * = denotes the presence of an isomeric peak within the region used to calculate the noise signal. n.d. = not determined.

Supporting Tables

Table S1: Table listing the origin of the lipid mediators employed in our studies and the respective extinction coefficients used to calculate their concentrations.

Mediator	Extinction coefficient		Source	Catalogue No/Reference
**DHA-derived mediators**				
RvD1	50000		Cayman Chemicals	Cat #: 10012554
17R-RvD1	50000		Cayman Chemicals	Cat #: 13060
RvD2	50000		Cayman Chemicals	Cat #: 10007279
RvD3	40000		Cayman Chemicals	Cat #: 13834
RvD4	40000		Cayman Chemicals	Cat #:13835
RvD5	30000		Cayman Chemicals	Cat #: 10007280
PD1	40000		Vinresol	
PDX	40000		Cayman Chemicals	Cat #: 10008128
17R-PD1	40000		Cayman Chemicals	Cat #: CAY34198
22-OH-PD1	40000		Prof Trond V. Hansen, University of Oslo	doi: 10.3390/ molecules24183228
PCTR1	40000		Cayman Chemicals	Cat #: 19064
PCTR2	40000		Cayman Chemicals	Cat #: 19065
PCTR3	40000		Cayman Chemicals	Cat #:19066
MaR1	40000		Cayman Chemicals	Cat #: 10878
MaR2	40000		Cayman Chemicals	Cat #: 16369
MCTR1	40000		Cayman Chemicals	Cat #: 17007
MCTR2	40000		Cayman Chemicals	Cat #: 17008
MCTR3	40000		Cayman Chemicals	Cat #: 17009
**n-3 DPA-derived mediators**				
RvT1	40000		Prof Charles N Serhan Brigham and Women's hospital and Prof Bernd Spurr Rowan University	doi.org/10.1016/ j.tetlet.2019.151473
RvT2	40000		Prof Charles N Serhan Brigham and Women's hospital and Prof Bernd Spurr Rowan University	doi.org/10.1016/ j.tetlet.2020.151857
RvT4	30000		Prof Charles N Serhan Brigham and Women's hospital and Prof Bernd Spurr Rowan University	doi.org/10.1016/ j.tetlet.2019.151473
$RvD1_{n-3\ DPA}$	50000		Prof Trond V. Hansen, University of Oslo	
$RvD5_{n-3\ DPA}$	30000		Cayman Chemicals	Cat #: 10546
$PD1_{n-3\ DPA}$	40000		Prof Trond V. Hansen, University of Oslo	doi: 10.3390/ molecules24183228.
**EPA-derived mediators**				

RvE1	40000		Cayman Chemicals	Cat #: 10007848
RvE2	30000		Vinresol	
RvE4	30000		Cayman Chemicals	Cat #: 29590
**AA-derived mediators**				
LXA_4	50000		Cayman Chemicals	Cat #: 10007271
15-epi-LXA_4	50000		Cayman Chemicals	Cat #: 90415
LXB_4	50000		Cayman Chemicals	Cat #: 90420
LTB_4	40000		Cayman Chemicals	Cat #: 10007240
20-OH-LTB_4	40000		Cayman Chemicals	Cat # 20190
LTC_4	40000		Cayman Chemicals	Cat #: 20210
LTD_4	40000		Cayman Chemicals	Cat #: 20310
LTE_4	40000		Cayman Chemicals	Cat #: 20410
PGD_2	#		Cayman Chemicals	Cat #: 10007202
PGE2	#		Cayman Chemicals	Cat #: 10007211
$PGF_{2\alpha}$	#		Cayman Chemicals	Cat # 16010
TXB_2	#		Cayman Chemicals	Cat #: 10007237
**Internal standards**				
d_4-RvE1	50000		Cayman Chemicals	Cat #: 10009854
d_5-MaR1	40000		Cayman Chemicals	Cat #: 21823
d_5-MaR2	40000		Cayman Chemicals	Cat #: 20757
d_5-PD1	40000		Cayman Chemicals	Cat #: 21625
d_4-LTB_4	40000		Cayman Chemicals	Cat #: 320110
d_4-LXA_4	50000		Cayman Chemicals	Cat #: 10007737
d_5-RvD3	40000		Cayman Chemicals	Cat #: 10009854
d_5-RvD2	50000		Cayman Chemicals	Cat #: 11184
d_5-17R-RvD1	50000		Cayman Chemicals	Cat #: 11183
d_5-PGE_2	#		Cayman Chemicals	Cat #: 314010
d_5-LTC_4	40000		Cayman Chemicals	Cat #: 10006198
d_5-LTD_4	40000		Cayman Chemicals	Cat #: 10006199
d5-LTE_4	40000		Cayman Chemicals	Cat #: 10007858

# denotes those mediators without a UV chromophore.

Table S2: List of MRM transitions and mass spectrometer parameters for each of the mediators evaluated.

A

Internal Standards	Q1	Q3	RT	DP (V)	EP (V)	CE (V)	CXP (V)
**d_4-RvE1**	353.3	197.2	8.5	-80	-10	-22.2	-12
	353.3	162.2	8.5	-80	-10	-27	-12
**d_5-RvD3**	380.2	147.1	11	-70	-4.8	-21.5	-10
	380.2	152.1	11	-70	-4.8	-21.5	-10
**d_5-RvD2**	380.2	175.2	10.8	-70	-10	-29.2	-12
	380.2	141.1	11	-70	-10	-21.5	-12
	380.2	135.1	11	-70	-4.8	-21.5	-10
**d_5-17R-RvD1**	380.2	141.1	11	-70	-10	-21.5	-12
	380.2	135.1	11	-70	-4.8	-21.5	-10
**d_4-PGE_2**	355.2	193.3	10.7	-33	-4.8	-24.3	-10
	355.2	275.3	10.7	-33	-4.8	-24.3	-10
**d_5-LXA_4**	356.2	222.4	11.4	-33	-4.8	-18	-10
	356.2	115.1	11.4	-33	-4.8	-18	-10
**d_5-MaR1**	364.3	123.2	13.6	-70	-10	-23.47	-10
	364.3	177.1	13.6	-70	-10	-20.5	-10
**d_5-MaR2**	364.3	177.1	14.3	-70	-4.8	-20.5	-10
	364.3	147.0	14.3	-70	-10	-17.2	-10
**d_5-PD1**	364.3	153.0	13.5	-70	-10	-17.2	-10
	364.3	123.0	13.5	-70	-10	-17.2	-10
**d_4-LTB_4**	339.2	197.2	13.9	-75.86	-12	-21.51	-12
	339.2	153.2	13.9	-75.86	-12	-26.32	-12
**d_5-LTE_4**	445.2	194.3	10.2	26	8.7	21.9	13
	445.2	306.3	10.2	26	8.7	21.9	13
**d_5-LTD4**	502.2	194.3	8.95	32	7	22.6	13
	502.2	306.3	8.95	32	7	22.6	13
**d_5-LTC_4**	631.3	194.3	9.85	34	6	28.5	13
	631.3	306.3	9.85	34	6	28.5	13

B

DHA-derived mediators	Q1	Q3	RT	DP (V)	EP (V)	CE (V)	CXP (V)
**RvD1**	375.2	233.2	11	-65	-10	-20.86	-10
	375.2	215.2	11	-65	-4.8	-25.8	-10
	375.2	141.1	11	-65	-10	-19.76	-10
	375.2	121.2	11	-65	-10	-37.3	-10
**17R-RvD1**	375.2	233.2	11	-65	-10	-20.86	-10
	375.2	215.2	11	-65	-4.8	-25.8	-10
	375.2	141.1	11	-65	-10	-19.76	-10
	375.2	121.2	11	-65	-10	-37.3	-10
**RvD2**	375.2	175.3	11	-65	-10	-30	-10
	375.2	215.2	11	-65	-4.8	-25.8	-10
	375.2	141.1	11	-65	-10	-19.76	-10
	375.2	121.2	11	-65	-10	-37.3	-10
**RvD3**	375.2	147.1	10.7	-75	-4.8	-26	-10
	375.2	137.0	10.7	-75	-4.8	-26	-10
	375.2	199.3	10.7	-75	-10	-30	-15
	375.2	115.1	10.7	-75	-10	-26	-10
**RvD4**	375.2	131.0	12.1	-75	-10	-19.9	-15
	375.2	101.0	12.1	-75	-4.8	-23	-10
	375.2	225.3	12.1	-75	-4.8	-27.1	-10
**RvD5**	359.3	199.2	13.3	-60	-4.8	-22.5	-10
	359.3	141.1	13.3	-60	-4.8	-18.3	-10
	359.3	225.4	13.3	-60	-10	-25.8	-15
**PD1**	359.3	153.1	13	-70	-4.8	-21.5	-10

	359.3	123.0	13	-70	-10	-19.63	-15
	359.3	137.1	13	-70	-4.8	-21	-10
**PDX**	359.3	153.1	13	-70	-4.8	-21.5	-10
	359.3	123.0	13	-70	-10	-19.63	-15
	359.3	137.1	13	-70	-4.8	-21	-10
**17R-PD1**	359.3	153.1	13	-70	-4.8	-21.5	-10
	359.3	123.0	13	-70	-10	-19.63	-15
	359.3	137.1	13	-70	-4.8	-21	-10
**22-OH-PD1**	375.3	204.1	9.9	-60	-10	-25	-10
	375.3	153.1	9.9	-60	-4.8	-24.5	-10
**PCTR1**	650.1	325.0	9.24	85	4	21	20
	650.1	231.0	9.24	100	6	27	20
	650.1	245.0	9.24	100	6	23	20
	650.1	308.0	9.24	100	6	20.2	20
	650.1	237.0	9.24	100	6	28.3	20
**PCTR2**	521.2	213.2	8.5	40	5	23.5	13
	521.2	325.0	8.5	75	8	14	20
	521.1	343.0	8.5	70	11	13.3	16
	521.2	231.0	8.5	75	8	19.6	20
	521.2	237.0	8.5	75	8	21.9	20
	521.2	245.0	8.5	75	8	18.8	20
**PCTR3**	464.2	231.0	9.8	70	8	22.1	19
	464.2	237.0	9.8	70	8	19	19
	464.1	213.1	9.8	40	5	23.5	13
	464.2	325.0	9.8	65	12	13.7	15
**Maresin 1**	359.3	221.1	13.8	-60	-4.8	-19	-10
	359.3	177.1	13.8	-60	-4.8	-20	-10
	359.3	250.3	13.8	-60	-10	-20.85	-10
**Maresin 2**	359.3	221.1	13.8	-60	-4.8	-19	-10
	359.3	177.1	13.8	-60	-4.8	-20	-10
	359.3	191.1	13.8	-60	-4.8	-19	-10
**MCTR1**	650.1	308.0	9.24	100	6	20.2	20
	650.1	325.0	9.24	85	4	21	20
	650.1	205.0	9.24	85	4	24.13	20
	650.1	215.0	9.24	85	4	24.46	20
	650.1	191.0	9.24	85	4	26.45	20
**MCTR2**	521.1	325.0	8.5	70	11	15.3	16
	521.1	343.0	8.5	70	11	13.3	16
	521.1	191.0	8.5	40	5	23.5	13
	521.1	205.0	8.5	70	11	16.26	16
	521.1	173.0	8.5	70	11	34	16
**MCTR3**	464.2	325.0	9.8	65	12	13.7	15
	464.2	191.0	9.8	40	5	23.5	13
	464.2	173.0	9.8	65	12	30.35	15
	464.2	205.0	9.8	65	12	14.9	15

C)

n-3 DPA-derived mediators	Q1	Q3	RT	DP (V)	EP (V)	CE (V)	CXP (V)
**RvT1**	377.3	193.1	10.5	-70	-4.8	-26.8	-10
	377.3	211.2	10.5	-70	-12	-22.21	-15
	377.3	143.2	11	-70	-12	-26.79	-15
**RvT2**	377.3	143.2	11	-70	-12	-26.79	-15
	377.3	227.2	11.3	-70	-12	-20.12	-15
	377.3	209.2	11.3	-70	-12	-24.73	-15
**RvT4**	361.3	143.2	13.7	-85	-12	-26	-15
	361.3	211.2	13.7	-85	-12	-20.85	-15
	361.3	193.1	13.7	-85	-4.8	-23	-10
**$RvD1_{n\text{-}3\ DPA}$**	377.3	143.1	11.4	-60	-4.8	-20.2	-10
	377.3	215.0	11.4	-60	-4.8	-26.8	-10

	377.3	121.0	11.4	-60	-4.8	-26.8	-10
	377.3	233.2	11.4	-60	-10	-18.69	-10
**RvD5$_{n\text{-}3\ DPA}$**	361.2	199.1	13.5	-80	-4.8	-22.5	-10
	361.2	245.2	13.5	-80	-10	-21.45	-15
	361.3	143.2	13.7	-85	-12	-26	-15
	361.2	263.1	13.5	-80	-4.8	-22	-10
**PD1$_{n\text{-}3\ DPA}$**	361.2	263.1	13.5	-80	-4.8	-22	-10
	361.2	183.1	13.7	-80	-4.8	-24	-10
	361.2	137.0	13.7	-80	-12	-28.8	-15
	361.2	155.2	13.7	-80	-12	-28.5	-15

D)

EPA-derived mediators	Q1	Q3	RT	DP (V)	EP (V)	CE (V)	CXP (V)
**RvE1**	349.3	195.1	8.3	-70	-4.8	-23.5	-10
	349.3	161.2	8.3	-70	-4.8	-23.5	-10
	349.3	205.2	8.3	-70	-10	-20.24	-15
**RvE2**	333.4	115.1	12.2	-70	-10	-17.76	-15
	333.4	253.3	12.2	-70	-10	-17.2	-15
	333.4	159.2	12.2	-70	-4.8	-25	-10
**RvE4**	333.4	115.1	12.2	-70	-10	-17.76	-15
	333.4	253.3	12.2	-70	-10	-17.2	-15
	333.4	235.3	12.2	-70	-4.8	-22	-10

E)

AA-derived mediators	Q1	Q3	RT	DP (V)	EP (V)	CE (V)	CXP (V)
**LXA_4**	351.1	217.2	11.3	-60	-4.8	-27.2	-10
	351.1	115.1	11.3	-60	-4.8	-19.5	-10
	351.1	235.3	11.3	-60	-10	-18.5	-10
**LXB_4**	351.2	221.2	10.8	-80	-10	-21.89	-10
	351.2	163.1	10.8	-80	-10	-22.92	-10
**15-epi-LXA4**	351.1	217.2	11.3	-60	-4.8	-27.2	-10
	351.1	115.1	11.3	-60	-4.8	-19.5	-10
**LTB_4**	335.3	195.3	13.7	-100	-10	-21.5	-10
	335.3	151.2	13.7	-100	-10	-24.6	-10
	335.3	129.4	13.7	-100	-10	-24	-10
**20-OH-LTB_4**	351.2	195.2	8.8	-88	-10	-24.9	-10
	351.2	129.1	8.8	-88	-10	-28.62	-10
	351.2	151.3	8.8	-88	-10	-28.18	-10
**LTC_4**	626.1	301.0	9.85	95	4	29.78	33
	626.1	308.0	9.85	95	4	27.81	33
	626.1	319.0	9.85	95	4	19	33
	626.1	189.0	9.85	95	9	28	33
	626.1	241.0	9.85	95	4	35	33
**LTD_4**	497.2	301.0	8.95	95	10	15	25
	497.2	189.0	8.95	95	10	22.9	25
**LTE_4**	440.1	189.0	10.2	70	10	20.47	25
	440.1	301.0	10.2	70	10	17.9	25
**PGE_2**	351.2	271.1	10.6	-60	-10	-24	-15
	351.2	189.1	10.6	-60	-4.8	-27.5	-10
	351.2	175.2	10.6	-60	-10	-30	-15
**PGD_2**	351.2	271.1	10.6	-60	-10	-24	-15
	351.2	189.1	10.6	-60	-4.8	-27.5	-10
	351.2	233.3	10.6	-60	-10	-17.9	-15
**$PGF_{2\alpha}$**	353.4	247.3	10.6	-90	-10	-30.91	-15
	353.4	171.3	10.6	-90	-10	-30.97	-15
	353.4	193.1	10.6	-90	-4.8	-34.5	-10
**TXB_2**	369.4	169.2	10.2	-55	-10	-22.5	-10
	369.4	195.0	10.2	-55	-10	-19.08	-10

	369.4	177.2	10.2	-55	-10	-29.66	-10

List of MRM pairs together with, retention time and respective mass spectrometer parameters for (A) deuterium labelled internal standards (B) DHA-derived mediators (C) n-3 DPA derived mediators, (D) EPA-derived mediators and (E) AA-derived mediators. RT = retention time, DP = declustering potential, EP = entrance potential, CE = collision energy, CXP = collision cell exit potential.

Table S3: Source parameters

**Negative mode**	
CUR	30
CAD	Med
Ionspray voltage (V)	-4500
TEM (ºC)	440
GS1	45
GS2	70

**Positive mode**	
CUR	30
CAD	Med
Ionspray voltage (V)	5500
TEM (ºC)	440
GS1	45
GS2	60

Table S4: Lipid mediator IS pairings used for quantitation

DHA-derived mediators	Q1	Q3	IS	Q1	Q3
RvD1	375.2	233.2	d_5-17R-RvD1	380.2	141.1
	375.2	215.2		380.2	141.1
	375.2	141.1		380.2	141.1
	375.2	121.2		380.2	141.1
17R-RvD1	375.2	233.2		380.2	141.1
	375.2	215.2		380.2	141.1
	375.2	141.1		380.2	141.1
	375.2	121.2		380.2	141.1
RvD2	375.2	175.3	d_5-RvD2	380.2	141.1
	375.2	215.2		380.2	141.1
	375.2	141.1		380.2	141.1
	375.2	121.2		380.2	141.1
RvD3	375.2	147.1	d_5-RvD3	380.2	152.1
	375.2	137.0		380.2	152.1
	375.2	199.3		380.2	152.1
	375.2	115.1		380.2	152.1
RvD4	375.2	131.0	d_5-RvD2	380.2	141.1
	375.2	101.0		380.2	141.1
	375.2	225.3		380.2	141.1
RvD5	359.3	199.2	d_5-MaR1	364.3	177.1
	359.3	141.1		364.3	177.1
	359.3	225.4		364.3	177.1
PD1	359.3	153.1	d_5-PD1	364.3	153.0
	359.3	123.0		364.3	153.0
	359.3	137.1		364.3	153.0
PDX	359.3	153.1		364.3	153.0
	359.3	123.0		364.3	153.0
	359.3	137.1		364.3	153.0
17R-PD1	359.3	153.1		364.3	153.0
	359.3	123.0		364.3	153.0
	359.3	137.1		364.3	153.0
22-OH-PD1	375.3	204.1	d_5-RvD2	380.2	141.1
	375.3	153.1		380.2	141.1
PCTR1	650.1	325.0	d_5-LTC_4	631.3	194.3
	650.1	231.0		631.3	194.3
	650.1	245.0		631.3	194.3
	650.1	308.0		631.3	194.3
	650.1	237.0		631.3	194.3
PCTR2	521.2	213.2	d_5-LTD_4	502.2	194.3
	521.2	325.0		502.2	194.3
	521.1	343.0		502.2	194.3
	521.2	231.0		502.2	194.3
	521.2	237.0		502.2	194.3
	521.2	245.0		502.2	194.3
PCTR3	464.2	231.0	d_5-LTE_4	445.2	194.3
	464.2	237.0		445.2	194.3
	464.1	213.1		445.2	194.3
	464.2	325.0		445.2	194.3
MaR1	359.3	221.1	d_5-MaR1	364.3	177.1
	359.3	177.1		364.3	177.1
	359.3	250.3		364.3	177.1
MaR2	359.3	221.1	d_5-MaR2	364.3	177.0
	359.3	177.1		364.3	177.0
	359.3	191.1		364.3	177.0

**MCTR1**	650.1	308.0	**d_5-LTC_4**	631.3	194.3
	650.1	325.0		631.3	194.3
	650.1	205.0		631.3	194.3
	650.1	215.0		631.3	194.3
	650.1	191.0		631.3	194.3
**MCTR2**	521.1	325.0	**d_5-LTD_4**	502.2	194.3
	521.1	343.0		502.2	194.3
	521.1	191.0		502.2	194.3
	521.1	205.0		502.2	194.3
	521.1	173.0		502.2	194.3
**MCTR3**	464.2	325.0	**d_5-LTE_4**	445.2	194.3
	464.2	191.0		445.2	194.3
	464.2	173.0		445.2	194.3
	464.2	205.0		445.2	194.3

(B)

**n-3 DPA-derived mediators**	**Q1**	**Q3**	**IS**	**Q1**	**Q3**
**RvT1**	377.3	193.1	**d_5-17R-RvD1**	380.2	141.1
	377.3	211.2		380.2	141.1
	377.3	143.2		380.2	141.1
**RvT2**	377.3	143.2		380.2	141.1
	377.3	227.2		380.2	141.1
	377.3	209.2		380.2	141.1
**RvT4**	361.3	143.2	**d_5-MaR1**	364.3	177.1
	361.3	211.2		364.3	177.1
	361.3	193.1		364.3	177.1
**$RvD1_{n\text{-}3\ DPA}$**	377.3	143.1	**d_5-17R-RvD1**	380.2	141.1
	377.3	215.0		380.2	141.1
	377.3	121.0		380.2	141.1
	377.3	233.2		380.2	141.1
**$RvD5_{n\text{-}3\ DPA}$**	361.2	199.1	**d_5-MaR1**	364.3	177.1
	361.2	245.2		364.3	177.1
	361.3	143.2		364.3	177.1
	361.2	263.1		364.3	177.1
**$PD1_{n\text{-}3\ DPA}$**	361.2	263.1	**d_5-MaR1**	364.3	177.1
	361.2	183.1		364.3	177.1
	361.2	137.0		364.3	177.1
	361.2	155.2		364.3	177.1

(C)

**EPA-derived mediators**	**Q1**	**Q3**	**IS**	**Q1**	**Q3**
**RvE1**	349.3	195.1	**d_4-RvE1**	353.3	197.2
	349.3	161.2		353.3	197.2
	349.3	205.2		353.3	197.2
**RvE2**	333.4	115.1	**d_4-LTB_4**	339.2	197.2
	333.4	253.3		339.2	197.2
	333.4	159.2		339.2	197.2
**RvE4**	333.4	115.1	**d_4-LTB_4**	339.2	197.2
	333.4	253.3		339.2	197.2
	333.4	235.3		339.2	197.2

(D)

AA-derived mediators	Q1	Q3	IS	Q1	Q3
LXA_4	351.1	217.2	d_5-LXA_4	356.2	222.4
	351.1	115.1		356.2	222.4
	351.1	235.3		356.2	222.4
LXB_4	351.2	221.2		356.2	222.4
	351.2	163.1		356.2	222.4
15-epi-LXA_4	351.1	217.2		356.2	222.4
	351.1	115.1		356.2	222.4
LTB_4	335.3	195.3	d_4-LTB_4	339.2	197.2
	335.3	151.2		339.2	197.2
	335.3	129.4		339.2	197.2
20-OH-LTB_4	351.2	195.2		339.2	197.2
	351.2	129.1		339.2	197.2
	351.2	151.3		339.2	197.2
LTC_4	626.1	301.0	d_5-LTC_4	631.3	194.3
	626.1	308.0		631.3	194.3
	626.1	319.0		631.3	194.3
	626.1	189.0		631.3	194.3
	626.1	241.0		631.3	194.3
LTD_4	497.2	301.0	d_5-LTD_4	502.2	194.3
	497.2	189.0		502.2	194.3
LTE_4	440.1	189.0	d_5-LTE_4	445.2	194.3
	440.1	301.0		445.2	194.3
PGE_2	351.2	271.1	d_4-PGE_2	355.2	275.3
	351.2	189.1		355.2	275.3
	351.2	175.2		355.2	275.3
PGD_2	351.2	271.1		355.2	275.3
	351.2	189.1		355.2	275.3
	351.2	233.3		355.2	275.3
$PGF_{2\alpha}$	353.4	247.3		355.2	275.3
	353.4	171.3		355.2	275.3
	353.4	193.1		355.2	275.3
TXB_2	369.4	169.2		355.2	275.3
	369.4	195.0		355.2	275.3
	369.4	177.2		355.2	275.3

Table S5: Range of standards used for the construction of standard curves for each of the transitions

	MRM transitions		Phase		Matrix	
**DHA-derived mediators**	**Q1**	**Q3**	Lowest Data Point (pg/inj)	Highest Data Point (pg/inj)	Lowest Data Point (pg/inj)	Highest Data Point (pg/inj)
**RvD1**	375	233	0.05	125	0.05	125
	375	141	0.05	125	0.05	125
	375	215	0.05	125	0.05	125
	375	121	0.05	125	0.05	125
**17R-RvD1**	375	233	0.05	125	0.05	125
	375	141	0.05	125	0.05	125
	375	215	0.05	125	0.05	125
	375	121	0.05	125	0.05	125
**RvD2**	375	175	0.1	125	0.1	125
	375	215	0.1	125	0.5	125
	375	121	0.15	125	0.05	125
**RvD3**	375	147	0.05	125	0.05	125
	375	137	0.05	125	1.5	125
	375	115	0.1	125	1.5	125
**RvD4**	375	131	0.05	125	1.5	125
	375	101	0.05	125	0.15	125
	375	225	0.1	125	0.1	125
**RvD5**	359	199	0.05	125	0.5	125
	359	225	0.1	125	0.05	125
	359	141	0.05	125	0.05	125
**PD1**	359	153	0.05	125	0.05	125
	359	123	0.05	125	0.05	125
	359	137	0.1	125	0.05	125
**PDx**	359	153	0.1	125	0.05	125
	359	137	0.05	125	0.3	125
**17R-PD1**	359	153	0.05	125	0.05	125
	359	137	0.1	125	0.5	125
**22-OH-PD1**	359	204	0.1	125	0.15	125
	359	153	0.05	125	0.15	125
**PCTR1**	650	213	0.05	125	1.5	125
	650	231	0.05	125	0.3	125
	650	308	0.05	125	0.05	125
**PCTR2**	521	213	0.5	125	0.3	125
	521	325	0.05	125	0.05	125
	521	231	0.05	125	0.3	125
	521	237	0.5	125	0.3	125
**PCTR3**	464	231	0.3	125	0.05	125
	464	237	0.3	125	0.5	125
	464	245	1.5	125	0.05	125
	464	213	0.3	125	0.05	125
**MaR1**	359	221	0.05	125	0.5	125
	359	177	0.05	125	0.1	125
	359	250	0.05	125	0.05	125
**MaR2**	359	221	0.05	125	0.1	125
	359	177	0.05	125	0.05	125
	359	191	0.05	125	0.15	125
**MCTR1**	650	191	0.05	125	0.5	125

	650	205	0.05	125	0.5	125
	650	173	0.1	125	0.05	125
**MCTR2**	521	325	0.05	125	0.05	125
	521	191	0.05	125	0.1	125
	521	205	0.1	125	0.3	125
	521	173	0.05	125	0.15	125
**MCTR3**	464	191	0.05	125	0.05	125
	464	173	0.1	125	0.05	125
	464	205	0.15	125	0.3	125
**n-3 DPA-derived mediators**						
**RvT1**	377	211	0.05	125	0.15	125
	377	143	0.1	125	0.3	125
**RvT2**	377	227	0.05	125	0.15	125
	377	143	0.05	125	0.05	125
**RvT3**	361	215	0.05	125	1.5	125
	361	173	0.05	125	1.5	125
	361	155	0.05	125	1.5	125
	361	143	0.05	125	1.5	125
**RvT4**	361	143	0.05	125	0.05	125
	361	211	0.05	125	0.15	125
	361	193	0.05	125	0.15	125
**$RvD1_{n-3 DPA}$**	377	143	0.05	125	0.1	125
	377	215	0.1	125	0.5	125
	377	233	0.1	125	1.5	125
**$RvD5_{n-3 DPA}$**	361	199	0.05	125	0.05	125
	361	245	0.05	125	0.05	125
	361	143	0.05	125	0.05	125
**$PD1_{n-3 DPA}$**	361	263	0.05	125	0.1	125
	361	183	0.1	125	0.5	125
	361	137	0.05	125	0.3	125
	361	155	0.05	125	1.5	125
**EPA-derived mediators**						
**RvE1**	349	195	0.05	125	0.05	125
	349	161	0.05	125	0.05	125
**RvE2**	333	115	0.1	125	0.05	125
	333	253	0.15	125	0.05	125
	333	159	0.05	125	0.05	125
**RvE4**	333	115	0.05	125	0.05	125
	333	253	0.05	125	0.05	125
**AA-derived mediators**						
**LXA_4**	333	217	0.1	125	0.5	125
	333	115	0.05	125	0.3	125
	351	235	0.05	125	0.3	125
**15-epi-LXA_4**	351	217	0.15	125	0.05	125
	351	115	0.05	125	0.05	125
**LXB_4**	351	221	1.55	125	1.55	125
	351	163	1.55	125	1.55	125
**LTB_4**	335	195	0.05	125	0.1	125
	335	151	0.1	125	0.3	125
	335	129	0.1	125	0.1	125
**LTC_4**	626	301	0.05	125	0.05	125
	626	319	0.05	125	0.15	125

	626	189	0.05	125	0.05	125
**LTD_4**	497	301	0.1	125	0.1	125
	497	189	0.05	125	0.15	125
	497	241	1.5	125	0.05	125
**LTE_4**	440	189	0.05	125	0.05	125
	440	301	0.05	125	0.05	125
	440	209	0.3	125	0.15	125
	440	199	0.15	125	0.05	125
**PGD_2**	351	271	0.05	125	0.05	125
	351	189	0.05	125	0.05	125
	351	233	0.05	125	0.05	125
**PGE_2**	351	271	0.05	125	0.05	125
	351	189	0.05	125	0.05	125
	351	175	0.05	125	1.5	125
**PGF_{2a}**	353	247	0.1	125	0.05	125
	353	171	0.05	125	0.1	125
**TXB_2**	369	169	0.1	125	0.1	125
	369	195	0.05	125	0.1	125

Standard curves were constructed for mediators derived form (A) DHA, (B) n-3 DPA, (C) EPA and AA. Table reports the lowest and highest data points used for each of the mediators. The 10 concentrations that were used for constructing calibration curves were: 0.05, 0.10, 0.15, 0.3, 0.5, 1.55, 4.65, 13.9, 41.7 and 125 pg per injection (pg/inj)

Table S6: Calibration curve parameters and LLOQ, LOD values.

**DHA-derived mediators**			Solvent					Matrix				
	**Q1**	**Q3**	**slope**	**intercept**	**r2**	**LLOQ (pg/inj)**	**LOD (pg/inj)**	**slope**	**intercept**	**r2**	**LLOQ (pg/inj)**	**LOD (pg/inj)**
**RvD1**	375.2	233.2	4.29E-03	5.34E-04	0.99	0.22	0.13	4.24E-03	1.63E-03	0.98	0.95	0.57
	375.2	215.2	5.71E-03	4.05E-04	0.99	0.05	0.03	5.10E-03	3.72E-03	0.98	2.37	1.42
	375.2	141.1	1.27E-02	1.04E-03	0.99	0.05	0.03	7.70E-03	2.20E-03	0.98	1.12	0.67
	375.2	121.2	4.27E-03	2.25E-04	0.99	0.13	0.08	3.25E-03	1.70E-03	0.98	0.71	0.42
**17R-RvD1**	375.2	233.2	9.91E-03	-4.27E-04	0.99	0.17	0.1	9.63E-03	2.47E-04	0.98	0.88	0.53
	375.2	215.2	1.28E-02	-5.03E-04	0.99	0.03	0.02	1.06E-02	6.22E-04	0.99	0.1	0.06
	375.2	141.1	2.85E-02	-1.39E-03	0.99	0.01	0.01	1.87E-02	3.60E-04	0.97	0.11	0.07
	375.2	121.2	1.02E-02	-6.41E-04	0.99	0.13	0.08	6.85E-03	7.54E-04	0.97	0.37	0.22
**RvD2**	375.2	175.3	2.07E-03	-1.34E-04	0.99	0.13	0.08	1.92E-03	1.52E-04	0.98	1.22	0.73
	375.2	215.2	1.05E-03	3.47E-06	0.98	0.11	0.07	1.06E-03	-6.88E-05	0.98	2.49	1.49
	375.2	141.1	2.29E-03	2.34E-04	0.98	0.41	0.24	2.01E-03	-8.70E-05	0.98	0.6	0.36
	375.2	121.2	2.52E-04	-5.91E-05	0.97	3.34	2	1.86E-04	6.64E-04	0.99	31.78	19.07
**RvD3**	375.2	147.1	2.56E-03	-3.59E-05	0.99	0.16	0.09	1.20E-02	6.99E-04	0.98	0.3	0.18
	375.2	137	3.38E-04	-1.84E-05	0.99	0.68	0.41	2.00E-03	-2.08E-03	0.97	13.95	10.43
	375.2	199.3	5.58E-05	4.95E-05	0.96	1.59	0.95	1.87E-04	-1.20E-03	0.97	38.58	23.15
	375.2	115.1	2.78E-04	-5.97E-06	0.99	0.39	0.24	9.48E-04	-2.85E-03	0.98	23.46	14.08
**RvD4**	375.2	131	1.90E-03	-1.80E-04	0.99	0.62	0.37	2.57E-03	-9.06E-04	0.98	1.93	1.16
	375.2	101	5.60E-03	-4.06E-04	0.99	0.1	0.06	8.91E-03	-6.52E-04	0.98	0.23	0.14
	375.2	225.3	8.84E-04	-1.02E-04	0.99	0.05	0.03	1.77E-03	-3.63E-04	0.99	5.62	3.37
**RvD5**	359.3	199.2	6.62E-03	-6.68E-04	0.98	0.17	0.1	6.03E-03	-2.08E-03	0.98	1.02	0.61
	359.3	141.1	4.11E-03	6.32E-04	0.99	0.68	0.41	3.54E-03	-1.45E-03	0.98	2.42	1.45
	359.3	225.4	6.50E-04	1.50E-05	0.98	0.33	0.2	7.28E-04	-8.06E-04	0.98	8.46	5.08
**PD1**	359.3	153.1	9.40E-03	-2.64E-05	0.99	0.03	0.02	1.89E-03	3.51E-04	0.98	1.81	1.09

	359.3	123	1.52E-04	7.19E-06	0.98	0.82	0.49	2.27E-03	-5.37E-04	0.98	1.88	1.13
	359.3	137.1	4.51E-04	2.86E-06	0.99	0.24	0.15	2.17E-03	1.02E-04	0.98	0.79	0.47
**PDX**	359.3	153.1	5.19E-03	-4.69E-04	0.99	0.17	0.1	4.22E-03	1.53E-05	0.98	0.68	0.41
	359.3	123	1.43E-04	1.23E-05	0.98	0.44	0.27	1.40E-04	-5.98E-04	0.99	32.18	19.31
	359.3	137.1	5.92E-04	3.26E-01	0.98	0.24	0.14	6.18E-04	-2.61E-04	0.99	3.44	2.07
**17R-PD1**	359.3	153.1	4.45E-03	-2.90E-04	0.98	0.04	0.02	3.27E-03	-4.73E-04	0.98	0.38	0.23
	359.3	123	3.66E-04	-5.69E-05	0.98	0.83	0.5	2.68E-04	-6.12E-04	0.99	12.97	7.78
	359.3	137.1	9.75E-04	-7.32E-05	0.98	0.13	0.08	8.94E-04	-4.67E-04	0.98	1.09	0.65
**22-OH-PD1**	375.3	204.1	8.31E-04	-8.46E-05	0.99	0.21	0.12	6.33E-04	-2.80E-04	0.98	3.41	2.05
	375.3	153.1	3.13E-04	-5.41E-05	0.99	0.26	0.15	6.24E-04	-4.85E-04	0.99	1.14	0.69
**PCTR1**	650.1	213	5.46E-04	3.48E-05	0.97	0.93	0.55	6.53E-04	-1.52E-03	0.98	9.53	5.71
	650.1	231	8.22E-04	-1.48E-04	0.98	0.94	0.57	9.78E-04	-3.96E-04	0.98	0.79	0.48
	650.1	308	5.19E-03	1.71E-03	0.97	1.32	0.79	6.27e-03	3.89e-03	0.98	1.39	0.83
**PCTR2**	521.2	213.2	5.24E-04	-1.94E-04	0.98	1.63	0.98	6.36E-04	-3.22E-04	0.98	3.37	2.02
	521.2	325	4.46E-03	-2.32E-04	0.97	0.05	0.03	3.99E-03	-1.69E-04	0.98	0.55	0.33
	521.2	231	7.78E-04	-1.73E-04	0.98	0.46	0.28	9.31E-04	-3.68E-04	0.98	1.76	1.06
	521.2	237	5.06E-04	-1.19E-04	0.98	0.37	0.22	6.08E-04	-4.26E-04	0.98	4.78	2.87
**PCTR3**	464.2	231	8.74E-04	-1.67E-04	0.97	1.04	0.63	1.11E-03	-3.85E-04	0.98	4.51	2.7
	464.2	237	5.60E-04	-1.18E-04	0.97	0.52	0.31	6.90E-04	-5.31E-04	0.98	3.72	2.23
	464.1	245	2.09E-04	-2.45E-04	0.96	2.7	1.62	2.47E-04	6.85E-04	0.93	19.2	11.51
	464.1	213.1	5.46E-04	-3.52E-04	0.96	2.12	1.27	6.33E-04	7.16E-04	0.97	0.63	0.38
**Maresin 1**	359.3	221.1	1.58E-03	1.14E-06	0.98	0.4	0.24	1.41E-03	-4.88E-04	0.98	2.89	1.73
	359.3	177.1	4.38E-03	-8.80E-05	0.98	0.05	0.03	4.49E-03	5.47E-04	0.98	0.95	0.57
	359.3	250.3	2.23E-03	-1.07E-04	0.98	0.08	0.05	2.23E-03	-2.99E-04	0.98	0.84	0.5
**Maresin 2**	359.3	221.1	8.53E-03	7.68E-05	1	0	0	6.79E-03	1.27E-03	0.99	0.72	0.43
	359.3	177.1	3.83E-03	2.20E-04	1	0.1	0.06	3.52E-03	1.26E-03	0.99	0.77	0.46
	359.3	191.1	2.39E-03	1.50E-05	1	0.13	0.08	1.91E-03	-5.37E-05	0.99	2.48	1.49

**MCTR1**	650.1	205	2.95E-04	-3.02E-04	0.97	1.36	0.81	3.11E-04	-6.21E-04	0.97	13.83	8.3
	650.1	215	5.39E-04	-4.25E-04	0.96	1.67	1	5.57E-04	-8.29E-04	0.98	10.17	6.1
	650.1	191	1.00E-03	-6.31E-05	0.96	0.61	0.37	1.12E-03	-5.14E-04	0.98	3.13	1.88
**MCTR2**	521.1	325	3.17E-03	3.60E-05	0.98	1.36	0.81	3.96E-03	-1.09E-04	0.98	0.30	0.18
	521.1	191	1.17E-03	-1.96E-04	0.98	0.36	0.21	1.29E-03	-3.47E-04	0.98	1.48	0.89
	521.1	205	2.67E-04	-8.48E-05	0.98	1.34	0.8	2.98E-04	-1.68E-04	0.98	3.36	2.02
	521.1	173	5.97E-04	-9.58E-05	0.98	0.43	0.26	6.77E-04	-4.84E-04	0.98	4.76	2.86
**MCTR3**	464.2	191	1.82E-03	-8.62E-05	0.96	0.77	0.46	2.09E-03	1.61E-03	0.97	5.7	3.42
	464.2	173	8.78E-04	-1.70E-04	0.96	0.83	0.5	9.60E-04	6.89E-04	0.98	3.62	2.17
	464.2	205	3.74E-04	-2.55E-04	0.95	2.29	1.37	4.16E-04	3.02E-04	0.97	5.32	3.19

(B)

**n-3 DPA-derived mediators**			**Solvent**					**Matrix**				
	**Q1**	**Q3**	**slope**	**intercept**	**r2**	**LLOQ**	**LOD**	**slope**	**intercept**	**r2**	**LLOQ**	**LOD**
**RvT1**	377.3	193.1	5.40E-03	-4.38E-04	0.98	0.15	0.09	3.41E-03	-3.40E-03	0.98	3.72	2.23
	377.3	211.2	1.20E-02	-1.45E-03	0.98	0.23	0.14	6.75E-03	-1.80E-03	0.98	0.89	0.53
	377.3	143.2	4.81E-03	-1.25E-03	0.98	1.08	0.65	2.91E-03	2.80E-04	0.98	0.58	0.35
**RvT2**	377.3	143.2	7.63E-03	-2.03E+00	0.99	0.08	0.05	4.57E-03	4.94E-04	0.99	0.57	0.34
	377.3	227.2	8.61E-03	-5.68E-04	0.99	0.10	0.06	5.32E-03	-4.15E-04	0.99	0.38	0.23
	377.3	209.2	3.70E-03	-1.84E-04	0.99	0.13	0.08	2.16E-03	7.93E-03	0.95	18.89	11.34
**RvT4**	361.3	143.2	5.08E-03	-6.21E-04	0.99	0.22	0.13	3.16E-03	-8.99E-04	0.98	2.63	1.58
	361.3	211.2	7.34E-03	-6.78E-04	0.98	0.09	0.06	6.00E-03	-1.43E-03	0.98	0.65	0.39
	361.3	193.1	4.11E-03	-3.97E-04	0.98	0.16	0.10	3.57E-03	2.66E-04	0.98	0.69	0.41
**$RvD1_{n\text{-}3\ DPA}$**	377.3	143.1	7.10E-03	-4.46E-04	0.99	0.11	0.06	4.60E-03	-1.86E-04	0.99	1.41	0.85
	377.3	215	1.94E-03	-1.39E-04	0.99	0.36	0.22	1.16E-03	1.11E-04	0.98	1.75	1.05
	377.3	121	5.87E-04	-9.60E-06	0.98	1.23	0.74	5.40E-04	8.26E-05	0.98	4.12	2.47
	377.3	233.2	7.15E-04	-1.78E-04	0.98	2.55	1.53	6.49E-04	1.00E-03	0.96	18.73	11.23
**$RvD5_{n\text{-}3\ DPA}$**	361.2	199.1	5.04E-03	-4.34E-04	0.98	0.17	0.10	4.59E-03	5.62E-04	0.98	2.33	1.40
	361.2	245.2	3.42E-03	-2.93E-04	0.99	0.15	0.09	1.99E-03	3.87E-04	0.98	3.11	1.87

	361.3	143.2	5.51E-03	-4.85E-04	0.98	0.17	0.10	4.36E-03	5.25E-05	0.98	2.22	1.33
	361.2	263.1	2.92E-03	-1.39E-04	0.98	0.35	0.21	2.89E-03	1.70E-03	0.98	1.46	0.88
**PD1$_{n-3\ DPA}$**	361.2	263.1	1.99E-03	-2.56E-04	0.98	0.90	0.54	1.99E-03	6.23E-05	0.98	3.50	2.10
	361.2	183.1	1.43E-03	-1.78E-04	0.98	0.54	0.32	1.13E-03	-2.65E-03	0.99	11.65	6.99
	361.2	137	1.59E-03	-1.06E-04	0.98	0.54	0.33	1.26E-03	-1.23E-03	0.98	10.29	6.17
	361.2	155.2	5.64E-04	-3.45E-05	0.98	0.76	0.46	4.32E-04	-8.20E-04	0.98	19.38	11.63

(C)

**EPA-derived mediators**			**Solvent**					**Matrix**				
	**Q1**	**Q3**	**slope**	**intercept**	**r2**	**LLOQ**	**LOD**	**slope**	**intercept**	**r2**	**LLOQ**	**LOD**
**RvE1**	349.3	195.1	3.73E-03	4.03E-05	1.00	0.15	0.09	9.95E-04	6.83E-04	0.98	1.17	0.70
	349.3	161.2	2.61E-03	-1.27E-04	1.00	0.14	0.08	8.63E-04	5.50E-04	0.98	0.89	0.54
	349.3	205.2	1.98E-03	-7.09E-05	1.00	0.04	0.03	6.21E-04	3.84E-04	0.98	0.67	0.40
**RvE2**	333.4	115.1	1.51E-03	-1.35E-04	0.99	0.10	0.06	1.33E-03	3.71E-04	0.98	0.35	0.21
	333.4	253.25	8.18E-04	-9.43E-05	0.99	0.11	0.07	8.24E-04	1.79E-04	0.98	0.43	0.26
	333.4	159.2	8.80E-04	-6.47E-05	0.99	0.17	0.10	7.54E-04	3.66E-05	0.98	0.79	0.47
**RvE4**	333.4	115.1	4.48E-03	4.44E-04	0.99	0.10	0.06	1.65E-03	4.60E-04	0.97	0.34	0.20
	333.4	253.25	2.07E-03	2.18E-04	0.99	0.04	0.02	8.93E-04	8.66E-05	0.98	0.43	0.26
	333.4	235.3	8.60E-04	9.14E-05	0.99	0.08	0.05	4.78E-04	2.19E-04	0.97	5.99	3.59

(D)

**AA-derived mediators**			**Solvent**					**Matrix**				
	**Q1**	**Q3**	**slope**	**intercept**	**r2**	**LLOQ**	**LOD**	**slope**	**intercept**	**r2**	**LLOQ**	**LOD**
**LXA$_4$**	351.1	217.2	8.16E-03	-4.80E-04	0.99	0.08	0.05	4.09E-03	-2.52E-03	0.99	2.89	1.73
	351.1	115.1	2.19E-02	-1.88E-04	0.99	0.22	0.13	1.01E-02	-1.48E-03	0.98	0.56	0.34
	351.1	235.3	5.38E-03	-5.40E-04	0.99	0.32	0.19	3.39E-03	-2.67E-03	0.98	3.8	2.28
**LXB$_4$**	351.2	221.2	5.78E-03	-1.02E-03	0.99	0.42	0.25	2.92E-03	-3.28E-03	0.99	8.51	5.1
	351.2	163.1	2.71E-03	-3.08E-04	0.99	0.51	0.31	1.37E-03	-2.00E-03	0.98	11.72	7.03
	351.2	115.1	4.47E+01	-2.77E-04	0.98	0	0	7.22E-04	-5.73E-04	0.98	18.32	10.99

**15-epi-LXA$_4$**	351.1	217.2	7.56E-03	-7.44E-04	0.99	0.03	0.02	3.95E-03	4.00E-03	0.97	5.67	3.4
	351.1	115.1	2.09E-02	-1.39E-03	0.99	0.09	0.05	9.58E-03	6.14E-03	0.98	2.86	1.72
**LTB$_4$**	335.3	195.3	2.61E-03	-1.55E-04	0.99	0.11	0.06	2.52E-03	-3.57E-04	0.99	1.19	0.71
	335.3	151.2	4.35E-04	-2.64E-05	0.99	0.04	0.02	4.22E-04	-3.83E-04	0.99	9.56	5.73
	335.3	129.4	2.78E-04	-2.47E-05	0.99	0.72	0.43	2.99E-04	2.87E-05	0.97	8.18	4.91
**20-OH-LTB$_4$**	351.2	195.2	1.45E-03	2.22E-04	0.99	0.08	0.05	2.81E-03	-4.78E-05	0.98	0.48	0.29
	351.2	129.1	1.43E-03	-3.64E-04	0.99	3.2	1.92	9.16E-04	-1.32E-03	0.98	5.83	3.5
	351.2	151.3	4.78E-05	-4.85E-05	0.98	8.66	5.2	5.24E-04	1.33E-04	0.98	1.7	1.02
**LTC$_4$**	626.1	301	7.60E-03	-2.51E-04	0.99	0.01	0.01	6.33E-03	-8.22E-04	0.99	0.62	0.37
	626.1	319	2.58E-03	-1.75E-04	0.99	0.1	0.06	2.29E-03	-1.32E-03	0.98	1.89	1.1
	626.1	189	7.99E-03	-3.70E-04	0.99	0.05	0.03	9.81E-03	-1.39E-03	0.98	0.68	0.41
**LTD$_4$**	497.2	301	7.41E-04	-3.51E-05	0.98	0.55	0.33	8.01E-04	-3.09E-04	0.98	2.84	1.71
	497.2	189	6.85E-04	-1.29E-04	0.98	0.71	0.43	7.60E-04	-2.75E-04	0.98	2.41	1.45
**LTE$_4$**	440.1	189	2.90E-03	-1.21E-04	0.98	0.05	0.03	3.19E-03	-4.93E-04	0.98	0.9	0.54
	440.1	301	2.94E-03	-1.44E-04	0.98	0.12	0.07	3.20E-03	-7.66E-04	0.98	1.51	0.91
**PGE$_2$**	351.2	271.1	5.38E-04	1.98E-05	0.98	0.03	0.02	3.73E-04	8.57E-05	0.98	0.29	0.18
	351.2	189.1	2.06E-04	4.13E-06	0.98	0.06	0.04	1.33E-04	2.89E-05	0.97	0.5	0.3
	351.2	175.2	5.25E-05	-8.50E-07	0.98	0.49	0.3	5.89E-05	-4.50E-05	0.98	6.78	4.07
**PGD$_2$**	351.2	271.1	3.26E-04	-2.35E-05	0.98	0.11	0.06	2.48E-04	6.39E-05	0.98	0.49	0.29
	351.2	189.1	2.94E-04	-2.10E-05	0.98	0.04	0.03	2.02E-04	4.60E-05	0.98	0.77	0.46
	351.2	233.3	2.68E-04	4.19E-08	0.98	0.28	0.17	2.72E-04	5.96E-04	0.94	13.42	8.05
**PGF$_{2a}$**	353.4	247.3	1.05E-04	-1.16E-05	0.98	0.2	0.12	6.54E-05	6.17E-06	0.98	1.92	1.15
	353.4	171.3	6.55E-05	-6.28E-06	0.98	0.46	0.28	1.26E-04	2.89E-05	0.98	0.7	0.42
	353.4	193.1	2.88E-04	-1.86E-05	0.98	0.13	0.08	1.75E-04	-2.30E-05	0.98	1.12	0.67
**TXB$_2$**	369.4	169.2	7.45E-04	-3.77E-05	0.98	0.09	0.05	5.43E-04	3.20E-04	0.98	1.89	1.13
	369.4	195	5.67E-04	-2.32E-05	0.98	0.15	0.09	4.23E-04	1.54E-04	0.98	4.54	2.72

LLOQ and LOD values are in pg/mL. Standard curves in solvent and in surrogate matrix were prepared in triplicate

Table S7: Comparison of signal intensity for each of the deuterium labelled mediators following plasma extraction using different extraction methodologies and matrices.

	n	Columns	PP	Loading buffer	Condtionning	Wash 1	Wash 2	Elution 1	Elution 2
**A**	24	Biotage Isolute C18	MeOH	H_2O/HCl (pH 3.5)	MeOH, Water	H_2O	Hexane	MF	MeOH
**B**	8	Biotage Isolute C18	ACN	H_2O/HCl (pH 3.5)	MeOH, Water	H_2O	Hexane	MF	MeOH
**C**	3	Biotage Isolute C18	MeOH/ACN	H_2O/HCl (pH 3.5)	MeOH, Water	H_2O	Hexane	MF	MeOH
**D**	3	Biotage Isolute C18	MeOH/Acetic acid	H_2O/HCl (pH 3.5)	MeOH, Water	H_2O	Hexane	MF	MeOH
**E**	3	Biotage Isolute C18	MeOH	H_2O/HCl (pH 3.5)	MeOH, Water	H_2O/MeOH (80/20)	Hexane	MF	MeOH
**F**	3	Biotage Isolute C18	MeOH	H_2O/HCl (pH 3.5)	MeOH, Water	H_2O /ACN (80/20)	Hexane	MF	MeOH
**G**	9	Biotage Evolute ABN	MeOH	H_2O/acetic acid (98/2)	-	H_2O/ MeOH (90/10)	-	MF	MeOH
**H**	8	Biotage Evolute ABN	MeOH	H_2O/acetic acid (98/2)	-	H_2O/MeOH (85/15)	-	MF	MeOH
**I**	18	Biotage Evolute ABN	MeOH	H_2O/acetic acid (98/2)	-	H_2O / MeOH (80/20)	-	MF	MeOH
**J**	3	Biotage Evolute ABN	MeOH	H_2O/acetic acid (98/2)	-	H_2O / MeOH (80/20)	-	MF	MeOH
**K**	6	Biotage Evolute ABN	MeOH	H_2O/acetic acid (98/2)	-	H_2O / MeOH (75/25)	-	MF	MeOH
**L**	3	Biotage Evolute ABN	MeOH	H_2O/acetic acid (98/2)	-	H_2O /ACN (85/15)	-	MF	MeOH
**M**	12	Biotage Evolute ABN	MeOH	PBS/acetic acid (pH 5.6)	MeOH, Water	H_2O	-	MF	MeOH
**N**	3	Biotage Evolute ABN	MeOH	PBS/acetic acid (pH 5.6)	MeOH, Water	H_2O	-	MF	MeOH
**O**	3	Biotage Evolute ABN	MeOH	PBS/acetic acid (pH 5.6)	MeOH, Water	H_2O	-	NH_4OH MF (98/2)	NH_4OH/MeOH (98/2)
**P**	3	Biotage Evolute AX	MeOH	H_2O/HCl (98/2)	-	Ammonium acetate/MeOH (95/5)	MeOH	MF/HCl (98/2)	Methanol/HCl (98/2)
**Q**	3	Biotage Evolute AX	MeOH	H_2O/acetic acid (98/2)	-	Ammonium acetate/MeOH (95/5)	MeOH	MF/acetic acid (98/2)	MeOH /acetic acid (98/2)
**R**	3	Biotage Evolute AX	MeOH	PBS/acetic acid (pH 6)	MeOH/ PBS (95/5), pH6	H_2O	MeOH/ H_2O (50/50)	Ethyl acetate/hexane (75/25) 1%acetic acid	MeOH/acetic acid (99/1)
**S**	3	Phenomenex Armstrong	MeOH	H_2O/MeOH (90/10)	MeOH, Water	10% MeOH	-	MF	MeOH

PP = protein precipitation; MeOH = methanol; MF = methyl formate; ACN = acetonitrile.

Table S8: Intraday and interday precision for standards in solvent

DHA-derived mediators	MRM transitions Q1	Q3	Intraday Standard Concentration (pg) 1.6	13.9	41.7	Interday Standard Concentration (pg) 1.6	13.9	41.7
RvD1	375	233	22.5%	2.9%	4.1%	19.0%	5.6%	4.3%
	375	141	12.0%	11.9%	5.4%	15.7%	7.1%	3.8%
	375	215	55.4%	5.8%	4.3%	49.7%	6.6%	3.6%
	375	121	12.6%	5.0%	2.8%	13.8%	6.3%	3.4%
17R-RvD1	375	233	1.5%	2.0%	2.9%	7.5%	2.5%	4.1%
	375	141	6.0%	4.2%	5.7%	2.0%	1.1%	5.0%
	375	215	3.8%	2.9%	3.0%	9.1%	4.0%	4.7%
	375	121	1.5%	2.0%	2.9%	5.6%	3.1%	3.9%
RvD2	375	175	5.1%	6.5%	1.7%	14.4%	6.5%	4.0%
	375	215	9.8%	6.6%	4.2%	20.4%	6.9%	6.3%
	375	121	13.1%	18.3%	12.9%	10.8%	15.1%	11.9%
RvD3	375	147	0.7%	4.6%	7.1%	6.0%	4.8%	5.1%
	375	137	25.0%	9.6%	2.7%	19.1%	10.9%	5.5%
	375	115	16.8%	6.2%	6.0%	21.7%	5.9%	5.1%
RvD4	375	131	9.3%	3.0%	2.2%	9.2%	3.7%	3.8%
	375	101	5.1%	1.9%	4.2%	6.9%	1.7%	3.2%
	375	225	9.7%	2.2%	3.4%	9.5%	2.9%	3.9%
RvD5	359	199	5.3%	4.7%	2.6%	10.8%	5.2%	2.2%
	359	225	78.6%	5.2%	6.2%	50.7%	8.2%	4.3%
	359	141	5.0%	5.0%	0.9%	10.5%	5.5%	2.0%
PD1	359	153	10.4%	5.4%	4.7%	9.4%	4.3%	3.9%
	359	123	2.8%	4.0%	4.0%	5.1%	6.9%	8.2%
	359	137	18.1%	5.7%	7.3%	22.4%	8.3%	6.5%
PDX	359	153	3.9%	5.7%	6.4%	5.3%	4.0%	4.3%

	359	137	14.3%	4.3%	4.5%	13.2%	3.5%	4.3%
**17R-PD1**	359	153	2.8%	7.1%	3.1%	4.6%	5.3%	3.4%
	359	137	4.0%	4.0%	6.0%	8.6%	4.9%	4.5%
**22-OH-PD1**	359	204	6.0%	4.5%	7.4%	9.7%	6.4%	4.7%
	359	153	23.9%	12.0%	6.1%	16.9%	7.3%	4.9%
**PCTR1**	650	213	7.0%	13.0%	8.8%	8.0%	7.7%	5.7%
	650	231	12.4%	11.0%	10.1%	12.2%	7.1%	22.9%
	650	308	4.2%	22.8%	6.5%	5.7%	11.8%	5.6%
**PCTR2**	521	213	5.3%	6.8%	9.4%	3.9%	5.0%	6.3%
	521	325	5.0%	17.2%	1.5%	4.4%	4.5%	2.3%
	521	231	7.3%	9.1%	2.4%	5.2%	5.1%	3.3%
	521	237	3.7%	7.5%	5.8%	3.8%	5.6%	4.7%
**PCTR3**	464	231	6.1%	4.5%	8.0%	6.1%	5.5%	4.6%
	464	237	3.7%	1.4%	11.0%	5.4%	4.7%	6.1%
	464	245	33.8%	8.3%	2.1%	38.5%	9.2%	4.5%
	464	213	12.4%	2.1%	16.7%	10.4%	4.8%	8.4%
**Maresin 1**	359	221	5.8%	4.7%	3.0%	9.3%	4.5%	3.7%
	359	177	16.8%	5.8%	2.1%	15.5%	5.3%	3.4%
	359	250	15.5%	5.0%	3.7%	11.2%	5.6%	3.4%
**Maresin 2**	359	221	9.1%	6.3%	1.8%	9.0%	3.6%	1.7%
	359	177	10.2%	9.1%	2.8%	20.8%	5.9%	3.0%
	359	191	12.5%	5.9%	3.8%	12.6%	5.7%	2.8%
**MCTR1**	650	191	12.3%	8.7%	15.2%	11.1%	5.2%	8.9%
	650	205	18.1%	14.0%	11.7%	24.4%	9.4%	9.4%
	650	173	24.7%	16.5%	33.0%	18.8%	12.6%	18.0%
**MCTR2**	521	325	4.9%	17.2%	1.5%	4.2%	8.0%	3.1%
	521	191	5.3%	8.1%	2.6%	5.2%	4.3%	2.9%
	521	205	4.4%	1.5%	5.6%	6.9%	2.5%	3.0%

	521	173	7.6%	6.6%	4.6%	5.8%	3.6%	4.2%
**MCTR3**	464	191	9.7%	22.8%	1.1%	5.4%	10.6%	2.7%
	464	173	11.4%	3.9%	8.3%	8.2%	4.7%	4.9%
	464	205	6.2%	4.9%	5.7%	6.1%	5.6%	4.9%
**n-3 DPA-derived mediators**								
**RvT1**	377	211	3.9%	4.6%	4.0%	7.9%	4.9%	3.3%
	377	143	14.7%	6.8%	4.0%	9.8%	6.0%	2.8%
**RvT2**	377	227	8.5%	2.2%	4.3%	10.4%	2.6%	3.6%
	377	143	11.0%	8.4%	4.5%	11.0%	11.8%	3.7%
**RvT4**	361	143	10.0%	3.4%	4.6%	11.7%	5.6%	8.0%
	361	211	6.4%	4.6%	3.0%	5.8%	3.0%	2.0%
	361	193	6.6%	8.4%	6.8%	11.4%	5.3%	4.3%
**RvD1 $_{n-3DPA}$**	377	143	8.0%	2.1%	2.0%	9.8%	3.5%	3.5%
	377	215	17.1%	3.6%	7.3%	16.9%	6.2%	7.0%
	377	233	27.0%	7.8%	8.3%	30.0%	11.2%	7.1%
**RvD5 $_{n-3DPA}$**	361	199	22.9%	6.0%	3.5%	22.3%	5.5%	3.3%
	361	245	21.4%	6.2%	1.9%	20.2%	7.8%	1.8%
	361	143	15.9%	3.9%	3.9%	15.1%	4.4%	6.1%
**PD1 $_{n-3DPA}$**	361	263	9.6%	8.4%	15.3%	5.6%	1.3%	3.4%
	361	183	9.3%	8.9%	1.0%	16.3%	7.4%	3.0%
	361	137	8.2%	3.1%	4.6%	7.1%	3.6%	3.8%
	361	155	5.8%	5.2%	5.2%	91.3%	10.5%	5.5%
**EPA-derived mediators**								
**RvE1**	349	195	7.8%	2.3%	3.5%	7.0%	2.4%	4.0%
	349	161	3.2%	2.4%	4.3%	5.2%	4.1%	4.0%
**RvE2**	333	115	2.7%	2.6%	2.5%	5.8%	3.0%	2.1%

	333	253	5.6%	3.1%	3.8%	4.1%	2.4%	3.8%
	333	159	9.4%	3.5%	3.1%	6.7%	3.6%	3.3%
**RvE4**	333	115	5.0%	1.1%	2.1%	3.8%	2.0%	2.9%
	333	253	3.5%	3.1%	1.5%	5.0%	3.3%	2.0%
**AA-derived mediators**								
**LXA_4**	351	217	9.2%	1.3%	4.3%	10.4%	3.5%	4.4%
	351	115	7.1%	5.1%	9.3%	49.2%	25.1%	23.3%
	351	235	10.4%	2.9%	6.9%	8.6%	4.0%	4.1%
**15-epi-LXA_4**	351	217	9.4%	2.5%	4.5%	16.9%	4.1%	3.0%
	351	115	8.2%	3.5%	7.1%	5.8%	4.2%	4.9%
**LXB_4**	351	221	17.9%	5.3%	6.5%	16.6%	5.5%	7.5%
	351	163	26.2%	7.0%	8.3%	22.7%	7.5%	7.4%
**LTB_4**	335	195	5.8%	5.7%	2.0%	6.8%	3.1%	2.2%
	335	151	13.2%	4.8%	3.1%	25.0%	3.8%	3.9%
	335	129	11.2%	1.9%	5.4%	26.6%	4.3%	5.3%
**LTC_4**	626	301	7.1%	11.6%	13.3%	5.1%	5.7%	6.5%
	626	319	6.8%	9.5%	8.1%	6.5%	5.3%	7.0%
	626	189	3.4%	11.1%	9.6%	4.4%	7.0%	6.3%
**LTD_4**	497	301	7.6%	5.5%	17.6%	6.8%	4.8%	8.7%
	497	189	4.3%	6.7%	2.5%	7.6%	4.6%	3.5%
	497	241	-	17.0%	10.0%	-	20.0%	11.0%
**LTE_4**	440	189	5.3%	6.8%	3.5%	6.2%	5.7%	4.6%
	440	301	2.7%	7.6%	3.2%	3.9%	6.6%	4.4%
	440	209	12.5%	8.4%	6.7%	10.7%	6.7%	6.4%
	440	199	9.7%	5.0%	2.7%	6.0%	5.2%	4.3%
**PGD_2**	351	271	9.6%	3.4%	2.9%	9.1%	4.6%	3.3%
	351	189	6.6%	7.0%	4.1%	10.2%	3.8%	3.8%

	351	233	15.7%	3.0%	4.7%	12.0%	3.7%	4.0%
**PGE_2**	351	271	9.9%	3.4%	2.9%	6.9%	2.7%	3.8%
	351	189	5.4%	1.8%	4.2%	10.4%	4.0%	3.8%
	351	175	4.3%	6.3%	3.1%	5.2%	6.3%	5.7%
**$PGF_{2\alpha}$**	353	247	12.3%	5.4%	3.9%	13.8%	4.9%	2.8%
	353	171	14.0%	2.9%	6.6%	30.6%	4.3%	5.1%
**TXB_2**	369	169	16.4%	2.5%	2.5%	13.4%	2.9%	2.9%
	369	195	20.4%	2.0%	3.3%	18.6%	2.7%	3.1%

- = No peak detected (intraday) or no peak detected in at least two of three replicate runs (interday).

Table S9: Intraday and interday accuracy for standards in solvent

			Intraday			Interday		
	MRM transitions		Standard Concentration (pg)			Standard Concentration (pg)		
DHA-derived mediators	Q1	Q3	***1.6***	***13.9***	***41.7***	***1.6***	***13.9***	***41.7***
RvD1	375	233	68%	78%	84%	75%	78%	83%
	375	141	77%	79%	88%	63%	73%	78%
	375	215	90%	89%	92%	87%	88%	92%
	375	121	80%	83%	84%	78%	80%	86%
17R-RvD1	375	233	82%	81%	84%	84%	78%	83%
	375	141	83%	84%	88%	78%	84%	87%
	375	215	82%	82%	86%	76%	80%	84%
	375	121	81%	81%	86%	78%	81%	87%
RvD2	375	175	81%	87%	91%	91%	89%	95%
	375	215	85%	97%	89%	104%	92%	97%
	375	121	93%	75%	80%	86%	84%	86%
RvD3	375	147	86%	82%	85%	87%	84%	88%
	375	137	80%	86%	85%	88%	88%	90%
	375	115	82%	83%	87%	101%	90%	92%
RvD4	375	131	116%	115%	122%	94%	94%	99%
	375	101	106%	111%	117%	92%	91%	96%
	375	225	113%	115%	116%	94%	91%	94%
RvD5	359	199	78%	80%	91%	75%	77%	84%
	359	225	82%	83%	95%	90%	83%	90%
	359	141	88%	85%	93%	89%	80%	87%
PD1	359	153	80%	82%	91%	81%	81%	87%
	359	123	84%	75%	78%	87%	74%	75%
	359	137	83%	66%	90%	110%	118%	134%
PDX	359	153	82%	86%	88%	110%	111%	118%

	359	137	76%	94%	100%	110%	122%	133%
17R-PD1	359	153	85%	82%	87%	107%	107%	116%
	359	137	89%	85%	90%	113%	109%	118%
22-OH-PD1	359	204	81%	89%	94%	95%	101%	107%
	359	153	90%	89%	91%	90%	84%	85%
PCTR1	650	213	139%	115%	121%	131%	107%	117%
	650	231	154%	141%	147%	145%	126%	135%
	650	308	160%	116%	118%	138%	105%	111%
PCTR2	521	213	130%	78%	79%	130%	78%	79%
	521	325	110%	87%	92%	107%	89%	95%
	521	231	104%	76%	74%	102%	75%	77%
	521	237	102%	68%	74%	100%	73%	77%
PCTR3	464	231	99%	74%	66%	99%	73%	70%
	464	237	84%	66%	66%	78%	70%	71%
	464	245	178%	74%	59%	182%	75%	67%
	464	213	119%	100%	79%	116%	93%	87%
MaR1	359	221	76%	78%	86%	82%	77%	85%
	359	177	78%	81%	90%	81%	79%	87%
	359	250	76%	78%	85%	77%	74%	81%
MaR2	359	221	95%	94%	97%	92%	93%	96%
	359	177	89%	92%	95%	95%	96%	96%
	359	191	98%	93%	96%	96%	92%	96%
MCTR1	650	191	129%	118%	115%	129%	108%	113%
	650	205	170%	141%	103%	184%	115%	105%
	650	173	158%	103%	88%	156%	93%	91%
MCTR2	521	325	110%	87%	92%	107%	89%	95%
	521	191	107%	76%	84%	104%	81%	87%
	521	205	139%	89%	88%	137%	89%	92%

	521	173	99%	82%	81%	96%	80%	84%
**MCTR3**	464	191	90%	61%	67%	87%	68%	70%
	464	173	83%	67%	71%	85%	71%	72%
	464	205	139%	79%	74%	144%	81%	77%
**n-3 DPA-derived mediators**								
**RvT1**	377	211	72%	66%	73%	76%	70%	75%
	377	143	97%	73%	76%	96%	76%	80%
**RvT2**	377	227	83%	80%	88%	84%	81%	87%
	377	143	80%	82%	85%	80%	81%	85%
**RvT4**	361	143	85%	90%	92%	83%	85%	89%
	361	211	72%	76%	83%	75%	74%	80%
	361	193	83%	75%	85%	82%	74%	81%
**RvD1 $_{n-3DPA}$**	377	143	81%	81%	88%	124%	93%	80%
	377	215	83%	81%	72%	83%	77%	78%
	377	233	91%	80%	76%	100%	76%	74%
**RvD5 $_{n-3DPA}$**	361	199	83%	81%	88%	80%	75%	80%
	361	245	74%	78%	83%	79%	74%	80%
	361	143	74%	71%	76%	76%	69%	72%
**PD1 $_{n-3DPA}$**	361	263	79%	84%	87%	83%	85%	91%
	361	183	77%	73%	79%	56%	54%	57%
	361	137	73%	65%	78%	75%	49%	56%
	361	155	77%	74%	83%	82%	59%	54%
**EPA-derived mediators**								
**RvE1**	349	195	92%	95%	97%	89%	92%	95%
	349	161	91%	92%	93%	88%	90%	92%
**RvE2**	333	115	86%	80%	86%	86%	81%	85%
	333	253	87%	82%	89%	89%	83%	87%

	333	159	84%	83%	85%	81%	81%	83%
**RvE4**	333	115	85%	82%	86%	85%	82%	86%
	333	253	82%	84%	87%	84%	83%	86%
**AA-derived mediators**								
**LXA_4**	351	217	84%	84%	93%	80%	79%	88%
	351	115	89%	84%	95%	83%	81%	87%
	351	235	93%	86%	95%	91%	83%	90%
**15-epi-LXA_4**	351	217	96%	93%	104%	88%	84%	91%
	351	115	98%	92%	103%	87%	83%	90%
**LXB_4**	351	221	92%	81%	91%	94%	79%	87%
	351	163	93%	79%	92%	88%	79%	86%
**LTB_4**	335	195	83%	81%	84%	86%	83%	87%
	335	151	80%	78%	82%	87%	80%	83%
	335	129	76%	78%	85%	90%	78%	84%
**LTC_4**	626	301	69%	68%	69%	69%	64%	68%
	626	319	69%	75%	75%	69%	67%	69%
	626	189	98%	94%	97%	92%	87%	90%
**LTD_4**	497	301	91%	72%	82%	94%	72%	78%
	497	189	103%	76%	78%	103%	76%	80%
	497	241	-	573%	88%	-	251%	83%
**LTE_4**	440	189	99%	82%	88%	94%	82%	87%
	440	301	85%	71%	71%	79%	70%	71%
	440	209	239%	97%	90%	223%	93%	85%
	440	199	159%	91%	85%	156%	86%	83%
**PGD_2**	351	271	79%	78%	82%	82%	79%	82%
	351	189	72%	78%	81%	75%	79%	84%
	351	233	80%	80%	83%	85%	79%	84%

**PGE_2**	351	271	70%	76%	79%	72%	74%	80%
	351	189	69%	75%	79%	75%	76%	82%
	351	175	77%	79%	80%	79%	76%	80%
**$PGF_{2\alpha}$**	353	247	82%	84%	87%	90%	87%	91%
	353	171	102%	88%	90%	103%	88%	94%
**TXB_2**	369	169	82%	83%	86%	90%	87%	92%
	369	195	85%	86%	89%	90%	88%	93%

- = No peak detected;

Table S10: Intra- and interday precision of the method using standards spiked in matrix.

			Intraday			Interday		
	MRM transitions		Standard Concentration (pg)			Standard Concentration (pg)		
**DHA-derived mediators**	**Q1**	**Q3**	**1.6**	**13.9**	**41.7**	**1.6**	**13.9**	**41.7**
**RvD1**	375	233	22.3%	5.0%	3.0%	13.0%	9.7%	4.1%
	375	141	23.7%	3.7%	2.9%	21.6%	6.0%	13.0%
	375	215	20.0%	7.9%	1.5%	49.5%	10.9%	7.4%
	375	121	13.0%	6.0%	5.4%	31.3%	8.6%	4.0%
**17R-RvD1**	375	233	5.4%	9.9%	1.3%	13.1%	7.9%	10.0%
	375	141	14.5%	8.8%	5.0%	0.1%	8.1%	7.4%
	375	215	8.7%	5.1%	2.5%	16.1%	6.9%	4.7%
	375	121	5.4%	9.9%	1.0%	22.8%	1.7%	3.0%
**RvD2**	375	175	18.3%	5.8%	6.4%	20.2%	11.0%	5.1%
	375	215	14.8%	8.8%	5.3%	21.6%	9.1%	4.1%
	375	121	-	0.6%	17.2%	-	33.6%	29.4%
**RvD3**	375	147	16.1%	3.5%	2.5%	13.3%	3.8%	3.5%
	375	137	15.9%	8.3%	6.5%	16.5%	8.6%	6.7%
	375	115	15.8%	12.6%	9.4%	13.2%	9.0%	8.2%
**RvD4**	375	131	8.1%	6.3%	4.1%	27.4%	7.0%	3.0%
	375	101	11.0%	6.2%	3.1%	10.8%	5.3%	4.2%
	375	225	18.0%	11.0%	4.2%	20.6%	14.1%	9.2%
**RvD5**	359	199	15.3%	6.2%	3.4%	13.5%	5.1%	5.6%
	359	225	52.7%	10.9%	4.6%	-	14.6%	12.2%
	359	141	14.2%	3.6%	4.8%	11.0%	8.0%	5.7%
**PD1**	359	153	33.0%	8.7%	4.9%	13.3%	8.9%	4.5%
	359	123	-	6.0%	3.0%	-	7.2%	6.4%
	359	137	-	19.2%	8.8%	27.6%	15.6%	10.0%
**PDX**	359	153	12.2%	6.5%	3.7%	11.4%	6.2%	3.5%
	359	137	14.1%	13.8%	4.8%	25.5%	9.4%	4.2%
**17R-PD1**	359	153	14.0%	10.7%	3.6%	17.6%	8.4%	3.1%
	359	137	16.0%	12.0%	8.0%	25.2%	10.3%	6.0%
**22-OH-PD1**	359	204	3.8%	15.1%	3.8%	12.7%	10.0%	4.6%
	359	153	-	21.8%	11.1%	-	12.9%	6.5%
**PCTR1**	650	213	6.4%	43.8%	10.3%	6.4%	45.2%	13.8%
	650	231	72.0%	13.6%	38.0%	64.0%	8.9%	80.7%
	650	308	15.0%	34.5%	16.5%	37.0%	28.3%	72.4%
**PCTR2**	521	213	43.0%	18.3%	18.5%	21.8%	49.4%	10.9%
	521	325	7.7%	16.6%	8.2%	25.6%	23.9%	4.8%
	521	231	4.8%	31.0%	12.7%	37.0%	35.4%	9.5%
	521	237	22.0%	24.4%	12.8%	3.0%	58.8%	11.6%
**PCTR3**	464	231	34.4%	16.5%	12.0%	26.6%	16.6%	18.8%

	464	237	8.6%	41.6%	7.2%	24.4%	40.2%	16.3%
	464	245	-	29.1%	20.5%	44.0%	52.5%	17.0%
	464	213	-	22.5%	8.9%	27.8%	15.9%	16.0%
**Maresin 1**	359	221	-	12.1%	5.7%	-	11.5%	4.4%
	359	177	20.3%	5.4%	3.2%	19.2%	6.1%	4.1%
	359	250	12.4%	7.8%	7.2%	8.0%	8.3%	4.9%
**Maresin 2**	359	221	7.2%	13.2%	5.0%	-	9.6%	40.5%
	359	177	12.1%	9.5%	4.5%	-	8.3%	39.1%
	359	191	20.2%	8.5%	5.3%	26.4%	13.6%	38.5%
**MCTR1**	650	191	35.5%	16.6%	24.5%	44.9%	26.8%	70.9%
	650	205	-	35.0%	51.5%	32.0%	36.0%	10.9%
	650	173	-	-	24.0%	-	-	-
**MCTR2**	521	325	7.6%	16.6%	8.2%	13.9%	27.8%	6.1%
	521	191	7.4%	22.3%	8.6%	12.7%	34.8%	7.1%
	521	205	53.0%	20.7%	8.7%	69.0%	11.1%	7.4%
	521	173	13.6%	24.4%	5.2%	19.6%	36.3%	6.8%
**MCTR3**	464	191	19.2%	13.3%	11.6%	31.2%	34.5%	25.9%
	464	173	34.5%	4.2%	9.2%	42.0%	18.1%	25.2%
	464	205	30.0%	8.0%	12.2%	93.0%	14.8%	29.4%
**n-3 DPA-derived mediators**								
**RvT1**	377	211	11.0%	6.7%	8.5%	13.9%	6.6%	6.7%
	377	143	21.5%	12.0%	7.4%	12.0%	8.4%	4.9%
**RvT2**	377	227	5.7%	12.4%	3.9%	11.3%	10.0%	18.4%
	377	143	30.4%	20.6%	15.0%	27.0%	13.5%	10.6%
**RvT4**	361	143	13.5%	7.4%	2.8%	17.9%	9.4%	5.5%
	361	211	17.2%	10.2%	4.9%	16.1%	7.2%	4.2%
	361	193	12.0%	10.2%	3.1%	14.5%	7.8%	3.8%
**$RvD1_{n-3\ DPA}$**	377	143	8.6%	6.1%	4.6%	22.7%	7.3%	4.8%
	377	215	6.2%	11.8%	2.6%	17.5%	10.5%	14.4%
	377	233	-	14.3%	6.8%	-	11.9%	9.9%
**$RvD5_{n-3\ DPA}$**	361	199	12.8%	12.6%	5.2%	11.8%	7.7%	4.9%
	361	245	15.9%	13.6%	5.0%	22.1%	8.8%	6.6%
	361	143	16.9%	10.7%	2.0%	20.4%	9.6%	3.6%
**$PD1_{n-3\ DPA}$**	361	263	-	14.1%	7.2%	6.4%	6.2%	4.9%
	361	183	29.9%	18.1%	4.9%	21.0%	12.2%	4.1%
	361	137	6.9%	16.2%	8.7%	6.9%	13.9%	21.2%
	361	155	-	10.5%	3.7%	-	19.3%	7.3%
**EPA-derived mediators**								
**RvE1**	349	195	12.6%	2.6%	4.3%	11.0%	3.7%	3.4%
	349	161	10.9%	6.0%	4.8%	15.0%	4.4%	2.7%
**RvE2**	333	115	5.7%	11.6%	5.5%	10.2%	7.5%	6.4%

	333	253	10.2%	8.7%	7.4%	21.5%	8.6%	5.0%
	333	159	3.1%	13.1%	5.3%	10.6%	9.9%	5.6%
**RvE4**	333	115	9.8%	9.2%	3.9%	9.8%	5.6%	6.6%
	333	253	6.9%	16.3%	4.8%	11.0%	8.6%	16.1%
**AA-derived mediators**								
**LXA_4**	351	217	7.9%	3.4%	6.3%	14.8%	6.3%	16.3%
	351	115	17.3%	6.2%	4.2%	16.1%	27.2%	19.1%
	351	235	11.3%	11.8%	8.0%	%	10.4%	8.1%
**15-epi-LXA_4**	351	217	9.5%	8.8%	9.8%	12.2%	7.2%	12.4%
	351	115	13.0%	5.8%	10.2%	10.0%	7.7%	8.3%
**LXB_4**	351	221	-	13.6%	4.5%	29.9%	21.4%	12.9%
	351	163	-	21.8%	9.0%	25.8%	20.5%	26.0%
**LTB_4**	335	195	9.2%	8.5%	4.5%	18.8%	7.2%	3.9%
	335	151	21.5%	16.0%	6.3%	29.6%	16.0%	7.8%
	335	129	12.0%	13.0%	5.0%	30.0%	14.9%	5.1%
**LTC_4**	626	301	34.4%	5.4%	11.3%	19.5%	9.0%	9.1%
	626	319	29.0%	12.5%	4.1%	18.6%	16.0%	5.7%
	626	189	10.7%	5.1%	6.2%	16.0%	8.8%	5.2%
**LTD_4**	497	301	21.3%	11.5%	7.9%	16.1%	13.5%	5.3%
	497	189	31.9%	15.5%	10.0%	26.2%	17.7%	15.6%
	497	241	-	-	-	-	-	-
**LTE_4**	440	189	13.6%	7.5%	4.2%	18.3%	5.3%	4.9%
	440	301	12.2%	8.2%	5.8%	9.2%	11.8%	7.5%
	440	209	-	-	-	-	-	-
	440	199	-	6.5%	9.2%	-	6.5%	7.4%
**PGD_2**	351	271	11.4%	4.4%	6.2%	23.3%	5.4%	7.4%
	351	189	15.2%	8.1%	7.6%	14.8%	4.9%	6.0%
	351	233	8.8%	4.2%	7.8%	14.3%	5.0%	5.3%
**PGE_2**	351	271	11.4%	4.4%	6.2%	9.4%	4.6%	5.7%
	351	189	11.5%	8.7%	4.4%	17.5%	7.4%	4.9%
	351	175	7.3%	8.6%	9.2%	-	10.3%	8.2%
**$PGF_{2\alpha}$**	353	247	10.2%	8.5%	6.5%	8.9%	7.3%	11.0%
	353	171	19.1%	2.1%	12.1%	15.3%	6.6%	8.7%
**TXB_2**	369	169	26.5%	3.4%	11.6%	12.5%	6.8%	60.9%
	369	195	17.8%	6.5%	7.6%	15.8%	5.7%	60.0%

- = No peak detected (intraday) or no peak detected in at least two of three replicate runs (interday).

Table S11: Intra- and interday accuracy of the method using standard curve prepared in solvent

			Intraday			Interday		
	MRM transitions		Standard Concentration (pg)			Standard Concentration (pg)		
DHA-derived mediators	Q1	Q3	*1.6*	*13.9*	*41.7*	*1.6*	*13.9*	*41.7*
RvD1	375	233	132%	96%	93%	130%	89%	90%
	375	141	85%	97%	100%	79%	74%	81%
	375	215	91%	97%	99%	102%	92%	99%
	375	121	103%	86%	94%	151%	81%	91%
17R-RvD1	375	233	92%	80%	80%	89%	80%	82%
	375	141	95%	90%	90%	98%	87%	88%
	375	215	107%	84%	83%	93%	80%	83%
	375	121	225%	96%	89%	163%	89%	92%
RvD2	375	175	56%	77%	80%	107%	90%	91%
	375	215	122%	89%	92%	134%	99%	108%
	375	121	-	455%	136%	-	238%	127%
RvD3	375	147	84%	74%	83%	90%	80%	87%
	375	137	117%	77%	84%	182%	91%	89%
	375	115	149%	74%	78%	163%	87%	91%
RvD4	375	131	113%	103%	94%	99%	84%	86%
	375	101	111%	91%	90%	94%	78%	82%
	375	225	142%	89%	91%	470%	78%	80%
RvD5	359	199	106%	90%	86%	102%	82%	85%
	359	225	127%	109%	85%	132%	102%	86%
	359	141	104%	87%	94%	103%	84%	92%
PD1	359	153	85%	73%	82%	124%	78%	82%
	359	123	-	62%	81%	-	67%	90%
	359	137	-	74%	86%	146%	127%	130%
PDX	359	153	79%	79%	84%	132%	107%	120%
	359	137	119%	101%	100%	159%	128%	140%
17R-PD1	359	153	90%	80%	82%	138%	113%	117%
	359	137	95%	88%	89%	132%	109%	127%
22-OH-PD1	359	204	82%	86%	85%	105%	111%	107%

	359	153	-	80%	80%	-	91%	82%
**PCTR1**	650	213	371%	119%	108%	291%	173%	109%
	650	231	335%	108%	128%	254%	130%	126%
	650	308	76%	112%	131%	91%	96%	114%
**PCTR2**	521	213	243%	89%	69%	174%	86%	63%
	521	325	147%	88%	78%	110%	85%	76%
	521	231	122%	75%	62%	105%	72%	59%
	521	237	212%	78%	66%	152%	69%	65%
**PCTR3**	464	231	189%	56%	64%	153%	59%	67%
	464	237	160%	56%	58%	141%	54%	61%
	464	245	-	82%	65%	251%	91%	66%
	464	213	-	76%	82%	109%	74%	78%
**MaR1**	359	221	-	77%	72%	-	75%	76%
	359	177	97%	78%	83%	114%	77%	82%
	359	250	92%	74%	78%	86%	70%	77%
**MaR2**	359	221	281%	147%	104%	-	113%	101%
	359	177	140%	90%	95%	-	101%	127%
	359	191	138%	84%	93%	126%	85%	91%
**MCTR1**	650	191	155%	137%	117%	138%	115%	119%
	650	205	-	141%	101%	355%	117%	101%
	650	173	-	-	115%	-	-	-
**MCTR2**	521	325	147%	88%	78%	108%	85%	76%
	521	191	115%	81%	71%	87%	74%	67%
	521	205	247%	93%	79%	173%	98%	74%
	521	173	162%	81%	68%	120%	76%	65%
**MCTR3**	464	191	131%	69%	59%	97%	67%	62%
	464	173	150%	70%	60%	111%	69%	65%
	464	205	170%	82%	63%	188%	76%	71%
**n-3 DPA-derived mediators**								
**RvT1**	377	211	79%	81%	94%	99%	92%	94%
	377	143	145%	93%	94%	135%	100%	100%
**RvT2**	377	227	118%	102%	102%	115%	98%	100%
	377	143	118%	102%	101%	124%	98%	100%

**RvT4**	361	143	99%	97%	102%	100%	85%	92%
	361	211	86%	81%	82%	80%	75%	79%
	361	193	119%	85%	83%	100%	78%	80%
**RvD1 $_{n\text{-}3DPA}$**	377	143	112%	110%	105%	163%	114%	103%
	377	215	128%	110%	107%	132%	100%	99%
	377	233	-	105%	89%	-	100%	93%
**RvD5 $_{n\text{-}3DPA}$**	361	199	87%	90%	81%	129%	82%	81%
	361	245	119%	77%	78%	134%	80%	79%
	361	143	72%	73%	64%	104%	69%	66%
**PD1 $_{n\text{-}3DPA}$**	361	263	-	84%	95%	279%	85%	97%
	361	183	130%	91%	87%	87%	53%	60%
	361	137	98%	91%	83%	119%	56%	53%
	361	155	-	84%	91%	-	68%	81%
**EPA-derived mediators**								
**RvE1**	349	195	104%	88%	88%	96%	86%	87%
	349	161	97%	85%	89%	95%	84%	87%
**RvE2**	333	115	47%	45%	44%	98%	77%	76%
	333	253	98%	52%	48%	113%	84%	83%
	333	159	59%	45%	44%	102%	74%	77%
**RvE4**	333	115	75%	60%	58%	108%	88%	90%
	333	253	70%	61%	61%	112%	91%	93%
**AA-derived mediators**								
**LXA$_4$**	351	217	86%	75%	84%	179%	78%	85%
	351	115	86%	80%	86%	74%	71%	81%
	351	235	107%	84%	90%	91%	81%	92%
**15-epi-LXA$_4$**	351	217	99%	81%	95%	283%	87%	90%
	351	115	82%	88%	94%	159%	83%	92%
**LXB$_4$**	351	221	-	102%	105%	189%	88%	92%
	351	163	-	89%	111%	601%	103%	101%
**LTB$_4$**	335	195	74%	42%	46%	104%	71%	70%
	335	151	95%	44%	46%	134%	76%	72%
	335	129	122%	48%	43%	187%	80%	71%

**LTC_4**	626	301	95%	66%	66%	83%	67%	67%
	626	319	122%	68%	73%	93%	69%	71%
	626	189	127%	91%	88%	105%	87%	90%
**LTD_4**	497	301	205%	81%	71%	150%	79%	77%
	497	189	149%	89%	72%	148%	82%	79%
	497	241	-	-	-	-	-	-
**LTE_4**	440	189	142%	97%	92%	134%	93%	87%
	440	301	96%	71%	65%	82%	65%	67%
	440	209	-	-	-	-	-	-
	440	199	-	70%	79%	-	69%	77%
**PGD_2**	351	271	112%	66%	77%	111%	71%	77%
	351	189	87%	65%	74%	86%	63%	69%
	351	233	107%	70%	76%	175%	69%	68%
**PGE_2**	351	271	73%	70%	79%	75%	68%	76%
	351	189	74%	65%	75%	73%	67%	74%
	351	175	96%	63%	77%	-	62%	69%
**$PGF_{2\alpha}$**	353	247	125%	87%	91%	133%	92%	90%
	353	171	115%	94%	90%	148%	94%	92%
**TXB_2**	369	169	132%	79%	88%	150%	88%	88%
	369	195	155%	83%	91%	163%	93%	90%

- = No peak detected (intraday) or no peak detected in at least two of three replicate runs (interday).

Table S12: Intra- and interday accuracy of the method using standard curve prepared in surrogate matrix

			Intraday			Interday		
	MRM transitions		Standard Concentration (pg)			Standard Concentration (pg)		
DHA-derived mediators	Q1	Q3	*1.6*	*13.9*	*41.7*	*1.6*	*13.9*	*41.7*
RvD1	375	233	112%	96%	94%	110%	89%	91%
	375	141	166%	218%	227%	151%	165%	183%
	375	215	18%	88%	95%	29%	83%	95%
	375	121	93%	100%	112%	151%	94%	108%
17R-RvD1	375	233	90%	89%	91%	87%	90%	93%
	375	141	146%	144%	144%	151%	139%	140%
	375	215	119%	99%	97%	102%	93%	97%
	375	121	313%	138%	128%	224%	127%	132%
RvD2	375	175	32%	66%	70%	77%	77%	79%
	375	215	100%	73%	75%	109%	81%	88%
	375	121	-	384%	114%	-	201%	107%
RvD3	375	147	79%	79%	90%	86%	85%	94%
	375	137	111%	85%	94%	185%	101%	100%
	375	115	79%	79%	90%	86%	85%	94%
RvD4	375	131	111%	85%	94%	185%	101%	100%
	375	101	57%	44%	43%	49%	38%	39%
	375	225	76%	37%	36%	205%	33%	32%
RvD5	359	199	127%	102%	97%	122%	93%	96%
	359	225	76%	37%	36%	-	33%	32%
	359	141	135%	101%	107%	134%	98%	105%
PD1	359	153	61%	81%	94%	106%	86%	93%
	359	123	-	7%	11%	-	8%	12%
	359	137	-	28%	35%	34%	50%	53%
PDX	359	153	90%	91%	98%	152%	124%	140%
	359	137	100%	94%	93%	138%	119%	131%
17R-PD1	359	153	108%	97%	99%	167%	137%	142%
	359	137	100%	82%	82%	134%	101%	117%
22-OH-PD1	359	204	73%	76%	75%	93%	99%	95%

	359	153	-	53%	51%	-	59%	52%
**PCTR1**	650	213	243%	101%	108%	249%	148%	93%
	650	231	176%	86%	93%	244%	116%	111%
	650	308	-	63%	100%	122%	101%	116%
**PCTR2**	521	213	135%	174%	131%	162%	69%	49%
	521	325	66%	96%	106%	92%	64%	57%
	521	231	70%	98%	92%	100%	56%	45%
	521	237	-	119%	117%	184%	59%	51%
**PCTR3**	464	231	72%	66%	94%	143%	44%	47%
	464	237	119%	109%	91%	168%	45%	45%
	464	245	-	118%	112%	-	40%	41%
	464	213	-	87%	106%	-	3%	6%
**MaR1**	359	221	-	96%	89%	-	94%	93%
	359	177	92%	91%	99%	112%	90%	97%
	359	250	98%	84%	89%	91%	80%	88%
**MaR2**	359	221	316%	174%	124%	181%	132%	120%
	359	177	136%	93%	98%	168%	104%	132%
	359	191	145%	94%	105%	132%	95%	102%
**MCTR1**	650	191	80%	123%	103%	170%	109%	109%
	650	205	-	99%	86%	473%	122%	96%
	650	173	-	-	102%	-	-	-
**MCTR2**	521	325	-	-	86%	87%	65%	58%
	521	191	85%	99%	96%	88%	62%	55%
	521	205	187%	104%	102%	168%	82%	60%
	521	173	80%	98%	95%	171%	69%	54%
**MCTR3**	464	191	142%	117%	105%	-26%	40%	44%
	464	173	99%	95%	98%	7%	44%	47%
	464	205	84%	108%	100%	10%	43%	49%
**n-3 DPA-derived mediators**								
**RvT1**	377	211	112%	114%	132%	140%	128%	132%
	377	143	130%	106%	111%	117%	115%	118%
**RvT2**	377	227	125%	110%	110%	122%	106%	109%
	377	143	124%	111%	110%	130%	107%	110%

**RvT4**	361	143	106%	104%	114%	128%	114%	118%
	361	211	106%	101%	102%	99%	93%	98%
	361	193	130%	96%	95%	108%	89%	91%
**RvD1 $_{n\text{-}3DPA}$**	377	143	123%	112%	107%	175%	117%	105%
	377	215	128%	120%	118%	133%	109%	109%
	377	233	-	128%	112%	-	120%	112%
**RvD5 $_{n\text{-}3DPA}$**	361	199	46%	95%	88%	93%	87%	89%
	361	245	135%	114%	112%	146%	90%	89%
	361	143	58%	100%	91%	104%	95%	93%
**PD1 $_{n\text{-}3DPA}$**	361	263	-	88%	95%	317%	88%	97%
	361	183	173%	129%	123%	113%	75%	85%
	361	137	271%	132%	110%	298%	88%	72%
	361	155	-	113%	131%	-	90%	116%
**EPA-derived mediators**								
**RvE1**	349	195	443%	411%	417%	408%	402%	412%
	349	161	343%	352%	372%	333%	349%	366%
**RvE2**	333	115	26%	44%	45%	78%	77%	77%
	333	253	68%	43%	41%	81%	71%	71%
	333	159	61%	46%	44%	105%	76%	78%
**RvE4**	333	115	342%	279%	266%	496%	407%	414%
	333	253	270%	222%	218%	420%	328%	335%
**AA-derived mediators**								
**LXA_4**	351	217	145%	126%	141%	301%	130%	143%
	351	115	169%	154%	165%	145%	137%	155%
	351	235	176%	117%	122%	153%	112%	124%
**15-epi-LXA_4**	351	217	149%	123%	143%	427%	132%	137%
	351	115	134%	153%	165%	270%	146%	161%
**LXB_4**	351	221	-	178%	181%	-	163%	162%
	351	163	-	158%	198%	-	192%	174%
**LTB_4**	335	195	60%	36%	40%	87%	62%	61%
	335	151	79%	38%	40%	114%	66%	63%
	335	129	78%	34%	31%	126%	58%	52%

**LTC_4**	626	301	114%	80%	80%	110%	81%	81%
	626	319	138%	93%	99%	93%	69%	70%
	626	189	84%	111%	101%	115%	87%	88%
**LTD_4**	497	301	80%	92%	103%	199%	87%	82%
	497	189	66%	107%	99%	187%	84%	77%
	497	241	-	-	-	-	-	-
**LTE_4**	440	189	-	-	104%	147%	91%	84%
	440	301	95%	93%	89%	106%	66%	66%
	440	209	-	-	-	-	-	-
	440	199	-	62%	119%	-	97%	82%
**PGD_2**	351	271	97%	60%	70%	95%	64%	70%
	351	189	80%	64%	73%	79%	61%	68%
	351	233	74%	58%	65%	133%	57%	58%
**PGE_2**	351	271	74%	75%	85%	76%	73%	82%
	351	189	80%	75%	87%	79%	77%	85%
	351	175	181%	77%	87%	-	77%	79%
**$PGF_{2\alpha}$**	353	247	104%	79%	84%	112%	83%	82%
	353	171	48%	79%	79%	77%	78%	81%
**TXB_2**	369	169	67%	63%	74%	83%	71%	74%
	369	195	77%	66%	78%	84%	75%	77%

- = No peak detected (intraday) or no peak detected in at least two of three replicate runs (interday)

Table S13: Summary table displaying the two transitions that gave overall best precision and accuracy values for each mediator

			Precision						Accuracy using std curve in Matrix						Accuracy using std curve in Phase					
			Intraday			Interday			Intraday			Interday			Intraday			Interday		
	MRM transitions		Standard Concentration (pg)			Standard Concentration (pg)			Standard Concentration (pg)			Standard Concentration (pg)			Standard Concentration (pg)			Standard Concentration (pg)		
**DHA-derived mediators**	**Q1**	**Q3**	**1.6**	**13.9**	**41.7**	**1.6**	**13.9**	**41.7**	***1.6***	***13.9***	***41.7***	***1.6***	***13.9***	***41.7***	***1.6***	***13.9***	***41.7***	***1.6***	***13.9***	***41.7***
**RvD1**	375	141	12%	12%	5%	16%	7%	4%	166%	218%	227%	151%	165%	183%	85%	97%	100%	79%	74%	81%
	375	121	13%	5%	3%	14%	6%	3%	93%	100%	112%	151%	94%	108%	103%	86%	94%	151%	81%	91%
**17R-RvD1**	375	233	2%	2%	3%	8%	3%	4%	90%	89%	91%	87%	90%	93%	92%	80%	80%	89%	80%	82%
	375	121	2%	2%	3%	6%	3%	4%	313%	138%	128%	224%	127%	132%	225%	96%	89%	163%	89%	92%
**RvD2**	375	175	5%	7%	2%	14%	7%	4%	32%	66%	70%	77%	77%	79%	56%	77%	80%	107%	90%	91%
	375	215	10%	7%	4%	20%	7%	6%	100%	73%	75%	109%	81%	88%	122%	89%	92%	134%	99%	108%
**RvD3**	375	147	1%	5%	7%	6%	5%	5%	79%	79%	90%	86%	85%	94%	84%	74%	83%	90%	80%	87%
	375	115	17%	6%	6%	22%	6%	5%	79%	79%	90%	86%	85%	94%	149%	74%	78%	163%	87%	91%
**RvD4**	375	131	9%	3%	2%	9%	4%	4%	111%	85%	94%	185%	101%	100%	113%	103%	94%	99%	84%	86%
	375	101	5%	2%	4%	7%	2%	3%	57%	44%	43%	49%	38%	39%	111%	91%	90%	94%	78%	82%
**RvD5**	359	199	5%	5%	3%	11%	5%	2%	127%	102%	97%	122%	93%	96%	106%	90%	86%	102%	82%	85%
	359	141	5%	5%	1%	11%	6%	2%	135%	101%	107%	134%	98%	105%	104%	87%	94%	103%	84%	92%
**PD1**	359	153	10%	5%	5%	9%	4%	4%	61%	81%	94%	106%	86%	93%	85%	73%	82%	124%	78%	82%
	359	123	3%	4%	4%	5%	7%	8%	-	7%	11%	-	8%	12%	-	62%	81%	-	67%	90%
**PDX**	359	153	4%	6%	6%	5%	4%	4%	90%	91%	98%	152%	124%	140%	79%	79%	84%	132%	107%	120%
	359	137	14%	4%	5%	13%	4%	4%	100%	94%	93%	138%	119%	131%	119%	101%	100%	159%	128%	140%
**17R-PD1**	359	153	3%	7%	3%	5%	5%	3%	108%	97%	99%	167%	137%	142%	90%	80%	82%	138%	113%	117%
	359	137	4%	4%	6%	9%	5%	5%	100%	82%	82%	134%	101%	117%	95%	88%	89%	132%	109%	127%
**22-OH-PD1**	359	204	6%	5%	7%	10%	6%	5%	73%	76%	75%	93%	99%	95%	82%	86%	85%	105%	111%	107%

	359	153	24%	12%	6%	17%	7%	5%	-	53%	51%	-	59%	52%	-	80%	80%	-	91%	82%
**PCTR1**	650	213	7%	13%	9%	8%	8%	6%	243%	101%	108%	249%	148%	93%	371%	119%	108%	291%	173%	109%
	650	308	4%	23%	7%	6%	12%	6%	-	63%	100%	122%	101%	116%	76%	112%	131%	91%	96%	114%
**PCTR2**	521	231	7%	9%	2%	5%	5%	3%	70%	98%	92%	100%	56%	45%	122%	75%	62%	105%	72%	59%
	521	237	4%	8%	6%	4%	6%	5%	-	119%	117%	184%	59%	51%	212%	78%	66%	152%	69%	65%
**PCTR3**	464	231	6%	5%	8%	6%	6%	5%	72%	66%	94%	143%	44%	47%	189%	56%	64%	153%	59%	67%
	464	237	4%	1%	11%	5%	5%	6%	119%	109%	91%	168%	45%	45%	160%	56%	58%	141%	54%	61%
**Maresin 1**	359	221	6%	5%	3%	9%	5%	4%	-	96%	89%	-	94%	93%	-	77%	72%	-	75%	76%
	359	250	16%	5%	4%	11%	6%	3%	98%	84%	89%	91%	80%	88%	92%	74%	78%	86%	70%	77%
**Maresin 2**	359	221	9%	6%	2%	9%	4%	2%	316%	174%	124%	181%	132%	120%	281%	147%	104%	-	113%	101%
	359	191	13%	6%	4%	13%	6%	3%	145%	94%	105%	132%	95%	102%	138%	84%	93%	126%	85%	91%
**MCTR1**	650	191	12%	9%	15%	11%	5%	9%	80%	123%	103%	170%	109%	109%	155%	137%	117%	138%	115%	119%
	650	205	18%	14%	12%	24%	9%	9%	-	99%	86%	473%	122%	96%	-	141%	101%	355%	117%	101%
**MCTR2**	521	191	5%	8%	3%	5%	4%	3%	85%	99%	96%	88%	62%	55%	115%	81%	71%	87%	74%	67%
	521	205	4%	2%	6%	7%	3%	3%	187%	104%	102%	168%	82%	60%	247%	93%	79%	173%	98%	74%
**MCTR3**	464	173	11%	4%	8%	8%	5%	5%	99%	95%	98%	7%	44%	47%	150%	70%	60%	111%	69%	65%
	464	205	6%	5%	6%	6%	6%	5%	84%	108%	100%	10%	43%	49%	170%	82%	63%	188%	76%	71%
**n-3 DPA-derived mediators**																				
**RvT1**	377	211	4%	5%	4%	8%	5%	3%	112%	114%	132%	140%	128%	132%	79%	81%	94%	99%	92%	94%
	377	143	15%	7%	4%	10%	6%	3%	130%	106%	111%	117%	115%	118%	145%	93%	94%	135%	100%	100%
**RvT2**	377	227	9%	2%	4%	10%	3%	4%	125%	110%	110%	122%	106%	109%	118%	102%	102%	115%	98%	100%
	377	143	11%	8%	5%	11%	12%	4%	124%	111%	110%	130%	107%	110%	118%	102%	101%	124%	98%	100%
**RvT4**	361	211	6%	5%	3%	6%	3%	2%	106%	101%	102%	99%	93%	98%	86%	81%	82%	80%	75%	79%
	361	193	7%	8%	7%	11%	5%	4%	130%	96%	95%	108%	89%	91%	119%	85%	83%	100%	78%	80%
**RvD1 $_{n-3DPA}$**	377	143	8%	2%	2%	10%	4%	4%	123%	112%	107%	175%	117%	105%	112%	110%	105%	163%	114%	103%

	377	215	17%	4%	7%	17%	6%	7%	128%	120%	118%	133%	109%	109%	128%	110%	107%	132%	100%	99%
**RvD5 n-3DPA**	361	245	21%	6%	2%	20%	8%	2%	135%	114%	112%	146%	90%	89%	119%	77%	78%	134%	80%	79%
	361	143	16%	4%	4%	15%	4%	6%	58%	100%	91%	104%	95%	93%	72%	73%	64%	104%	69%	66%
**PD1 $_{n\text{-}3DPA}$**	361	263	10%	8%	15%	6%	1%	3%	-	88%	95%	317%	88%	97%	-	84%	95%	279%	85%	97%
	361	137	8%	3%	5%	7%	4%	4%	271%	132%	110%	298%	88%	72%	98%	91%	83%	119%	56%	53%
**EPA-derived mediators**																				
**RvE1**	349	195	8%	2%	4%	7%	2%	4%	443%	411%	417%	408%	402%	412%	104%	88%	88%	96%	86%	87%
	349	161	3%	2%	4%	5%	4%	4%	343%	352%	372%	333%	349%	366%	97%	85%	89%	95%	84%	87%
**RvE2**	333	115	3%	3%	3%	6%	3%	2%	26%	44%	45%	78%	77%	77%	47%	45%	44%	98%	77%	76%
	333	253	6%	3%	4%	4%	2%	4%	68%	43%	41%	81%	71%	71%	98%	52%	48%	113%	84%	83%
**RvE4**	333	115	5%	1%	2%	4%	2%	3%	342%	279%	266%	496%	407%	414%	75%	60%	58%	108%	88%	90%
	333	253	4%	3%	2%	5%	3%	2%	270%	222%	218%	420%	328%	335%	70%	61%	61%	112%	91%	93%
**AA-derived mediators**																				
**LXA_4**	351	217	9%	1%	4%	10%	4%	4%	145%	126%	141%	301%	130%	143%	86%	75%	84%	179%	78%	85%
	351	235	10%	3%	7%	9%	4%	4%	176%	117%	122%	153%	112%	124%	107%	84%	90%	91%	81%	92%
**15-epi-LXA_4**	351	217	9%	3%	5%	17%	4%	3%	149%	123%	143%	427%	132%	137%	99%	81%	95%	283%	87%	90%
	351	115	8%	4%	7%	6%	4%	5%	134%	153%	165%	270%	146%	161%	82%	88%	94%	159%	83%	92%
**LXB_4**	351	221	18%	5%	7%	17%	6%	8%	-	178%	181%	-	163%	162%	-	102%	105%	189%	88%	92%
	351	163	26%	7%	8%	23%	8%	7%	-	158%	198%	-	192%	174%	-	89%	111%	601%	103%	101%
**LTB_4**	335	195	6%	6%	2%	7%	3%	2%	60%	36%	40%	87%	62%	61%	74%	42%	46%	104%	71%	70%
	335	151	13%	5%	3%	25%	4%	4%	79%	38%	40%	114%	66%	63%	95%	44%	46%	134%	76%	72%
**LTC4**	626	319	7%	10%	8%	7%	5%	7%	138%	93%	99%	93%	69%	70%	122%	68%	73%	93%	69%	71%
	626	189	3%	11%	10%	4%	7%	6%	84%	111%	101%	115%	87%	88%	127%	91%	88%	105%	87%	90%
**LTD_4**	497	301	8%	6%	18%	7%	5%	9%	80%	92%	103%	199%	87%	82%	205%	81%	71%	150%	79%	77%
	497	189	4%	7%	3%	8%	5%	4%	66%	107%	99%	187%	84%	77%	149%	89%	72%	148%	82%	79%
**LTE_4**	440	189	5%	7%	4%	6%	6%	5%	-	-	104%	147%	91%	84%	142%	97%	92%	134%	93%	87%

	440	301	3%	8%	3%	4%	7%	4%	95%	93%	89%	106%	66%	66%	96%	71%	65%	82%	65%	67%
**PGD_2**	351	271	10%	3%	3%	9%	5%	3%	97%	60%	70%	95%	64%	70%	112%	66%	77%	111%	71%	77%
	351	189	7%	7%	4%	10%	4%	4%	80%	64%	73%	79%	61%	68%	87%	65%	74%	86%	63%	69%
**PGE_2**	351	271	10%	3%	3%	7%	3%	4%	74%	75%	85%	76%	73%	82%	73%	70%	79%	75%	68%	76%
	351	189	5%	2%	4%	10%	4%	4%	80%	75%	87%	79%	77%	85%	74%	65%	75%	73%	67%	74%
**$PGF_{2\alpha}$**	353	247	12%	5%	4%	14%	5%	3%	104%	79%	84%	112%	83%	82%	125%	87%	91%	133%	92%	90%
	353	171	14%	3%	7%	31%	4%	5%	48%	79%	79%	77%	78%	81%	115%	94%	90%	148%	94%	92%
**TXB_2**	369	169	16%	3%	3%	13%	3%	3%	67%	63%	74%	83%	71%	74%	132%	79%	88%	150%	88%	88%
	369	195	20%	2%	3%	19%	3%	3%	77%	66%	78%	84%	75%	77%	155%	83%	91%	163%	93%	90%

Table S14: Evaluation of quantitation accuracy following sample dilution

			Area ratio at dilution				± % undiluted		
Analyte	Q1	Q3	0	1:1	1:2	1:3	1:1	1:2	1:3
**DHA-derived mediators**									
RvD1	375	141	6.11	5.32	4.64	3.85	-12.90%	-24.00%	-37.00%
17R-RvD1	375	233	5	4.78	4.78	4.81	-4.40%	-4.40%	-3.90%
RvD2	375	175	3.44	4.11	3.23	3.29	19.60%	-6.10%	-4.10%
RvD3	375	147	6.14	5.36	5.08	4.77	-12.70%	-17.30%	-22.30%
RvD4	375	101	14.6	17.1	15.7	16.14	17.40%	7.60%	10.70%
RvD5	359	199	84.6	80.7	73.5	75.1	-4.70%	-13.20%	-11.30%
PD1	359	153	*	*	*	*	*	*	*
PDX	359	153	125.9	116.4	113.1	118.6	4.70%	2.10%	2.50%
17R-PD1	359	137	2.45	7.06	6.75	8.18	188.00%	175.50%	233.90%
MaR1	359	221	71.2	71.5	61	73.5	0.40%	-14.30%	3.30%
MaR2	359	221	51.5	49	49.7	46.4	-4.80%	-3.60%	-10.00%
**n-3 DPA-derived mediators**									
RvT1	377	211	13.7	8.92	10	17	-34.80%	-27.20%	24.30%
RvT2	377	227	0.14	0.17	0.1	0.14	21.90%	-30.20%	0.02%
RvT4	361	193	14.8	18.2	18.1	21	23.10%	22.20%	41.90%
$RvD1_{n\text{-}3DPA}$	377	143	7.7	8.01	7.68	8.09	4.00%	-0.20%	5.00%
$RvD5_{n\text{-}3DPA}$	361	199	47	52.8	50.2	61.6	12.30%	6.80%	31.10%
$PD1_{n\text{-}3DPA}$	361	263	58.5	69	66.4	76.9	18.00%	13.60%	31.50%
**EPA-derived mediators**									
RvE1	349	195	2.71	2.86	2.95	2.98	5.30%	8.80%	9.70%
RvE2	333	115	10.17	10.65	10.38	10.43	4.70%	2.10%	2.50%
RvE4	333	253	5.03	5.16	5.45	5.1	2.50%	8.20%	1.30%
**AA-derived mediators**									
LXA_4	351	235	3.7	4	3.9	4.1	9.30%	7.10%	11.60%
15-epi-LXA_4	351	115	60	82.3	80	77.2	37.30%	33.40%	28.70%
LXB_4	351	221	6	4.88	4.15	5.69	-18.70%	-30.90%	-5.20%
LTB_4	335	195	367	339	318	306	-7.60%	-13.20%	-16.50%
PGD_2	351	189	0.2	0.2	0.15	0.05	2.60%	-26.70%	-74.70%
PGE_2	351	189	0.06	0.07	0.04	0.04	3.50%	-37.70%	-40.20%
$PGF_{2\alpha}$	353	171	0.1	0.11	0.09	0.03	6.40%	-8.60%	-65.40%
TXB_2	369	169	0.42	0.95	0.88	0.35	128.30%	111.00%	-15.90%

* = denoted below LLOQ

Table S15: Lipid mediator stability in extracted matrix at 4ºC.

Stability	Q1	Q3		± % 0d			
Analyte		Primary	Secondary	1d	3d	14d	21d
**DHA-derived mediators**							
RvD1	375	141	215	-12%	-22%	-20%	-15%
17R-RvD1	375	233	121	14%	1%	24%	1%
RvD2	375	175	215	2%	7%	25%	12%
RvD3	375	147	137	-2%	-5%	5%	4%
RvD4	375	101	131	-22%	-35%	-32%	-33%
RvD5	359	199	141	5%	8%	10%	4%
PD1	359	153	137	12%	20%	23%	16%
PDX	359	153	137	-1%	28%	5%	0.3%
17R-PD1	359	137	153	10%	15%	-17%	-4%
MaR1	359	221	177	17%	6%	22%	3%
MaR2	359	221	177	-5%	14%	23%	16%
**n-3 DPA-derived mediators**							
RvT1	377	211	193	2%	-3%	-72%	-72%
RvT2	377	227	143	6%	-6%	-15%	-14%
RvT4	361	193	211	0.5%	7%	15%	3%
$RvD1_{n-3DPA}$	377	143	215	26%	6%	11%	24%
$RvD5_{n-3DPA}$	361	199	245	-6%	2%	21%	7%
$PD1_{n-3DPA}$	361	263	137	12%	-2%	*	*
**EPA-derived mediators**							
RvE1	349	195	161	-2%	7%	8%	-6%
RvE2	333	115	159	-19%	-16%	-16%	-12%
RvE4	333	253	115	-9%	5%	-29%	-22%
**AA-derived mediators**							
LXA_4	351	235	115	14%	23%	27%	1%
LXB_4	351	221	163	2%	3%	-80%	-73%
15-epi-LXA_4	351	115	217	-7%	9%	-11%	-16%
LTB_4	335	195	151	10%	14%	-1%	-24%
PGD_2	351	189	233	24%	28%	40%	38%
PGE_2	351	189	271	7%	26%	1%	2%
$PGF_{2\alpha}$	353	171	193	-18%	-10%	264%	189%
TXB_2	369	169	195	2%	-5%	44%	20%

Points marked in blue denote value above 25%, and in orange denote values higher than the Acceptable Change Limit (ACL) as specified in ISO 5725-6:1994. The ACL is an interpretation of measured difference based on a defined analytical imprecision (CVa), with the formula ACL=2.77 CVa. Here we employed a CVa value of 25%, which was used to qualify transitions with acceptable precision. The factor is determined by the bi-directional 95% confidence interval, as described here[1]

Table S16: Comparison of signal to noise ratios obtained using different algorithms and an external blank.

	MRM transitions		USP*	RN&	PtP&	SD&
DHA-derived mediators	Q1	Q3				
RvD1	375	233	6	8	2	9
	375	141	16	15	4	10
	375	215	9	8	5	12
	375	121	5	5	4	10
17R-RvD1	375	233	14	11	5	17
	375	141	22	21	6	19
	375	215	14	16	2	7
	375	121	7	13	4	12
RvD2	375	175	9	11	5	14
	375	215	5	7	4	9
	375	121	14	8	4	9
RvD3	375	147	24	38	8	27
	375	137	6	11	4	12
	375	115	-	-	-	-
RvD4	375	131	9	16	7	23
	375	101	23	27	7	22
	375	225	-	-	-	-
RvD5	359	199	22	19	8	18
	359	225	-	-	-	-
	359	141	18	12	5	15
PD1	359	153	21	11	10	29
	359	123	-	-	-	-
	359	137	-	-	-	-
PDx	359	153	46	16	9	29
	359	137	6	9	4	9
17R-PD1	359	153	17	12	7	19
	359	137	5	11	4	10
22-OH-PD1	359	204	12	18	8	25
	359	153	-	-	-	-
PCTR1	650	213	3	6	2	4
	650	231	3	10	4	7
	650	308	25	21	6	16
PCTR2	521	213	2	9	4	7
	521	325	47	51	16	53
	521	231	3	10	5	7
	521	237	4	8	4	7
PCTR3	464	231	3	7	4	6
	464	237	8	9	5	15
	464	245	2	4	2	6
	464	213	-	-	-	-
MaR1	359	221	-	-	-	-
	359	177	15	15	6	14
	359	250	18	32	14	37
MaR2	359	221	-	-	-	-
	359	177	-	-	-	-
	359	191	9	11	4	12
MCTR1	650	191	5	12	3	7
	650	205	4	9	3	7
	650	173	-	-	-	-
MCTR2	521	325	42	43	16	34
	521	191	8	16	9	13
	521	205	6	11	6	8
	521	173	6	8	6	7

**MCTR3**	464	191	6	6	5	12
	464	173	6	6	2	4
	464	205	5	8	4	7
**n-3 DPA-derived mediators**						
**RvT1**	377	211	20	14	7	21
	377	143	9	10	3	10
**RvT2**	377	227	20	25	7	25
	377	143	9	13	5	13
**RvT3**	361	215	-	-	-	-
	361	173	-	-	-	-
	361	155	-	-	-	-
	361	143	-	-	-	-
**RvT4**	361	143	13	12	6	16
	361	211	21	22	8	22
	361	193	10	9	3	9
**RvD1$_{n\text{-}3\ DPA}$**	377	143	13	10	4	12
	377	215	-	-	-	-
	377	233	-	-	-	-
**RvD5$_{n\text{-}3\ DPA}$**	361	199	6	17	6	22
	361	245	14	12	5	13
	361	143	8	13	5	18
**PD1$_{n\text{-}3\ DPA}$**	361	263	13	6	4	10
	361	183	5	7	4	12
	361	137	5	12	7	22
	361	155	-	-	-	-
**EPA-derived mediators**						
**RvE1**	349	195	14	29	5	19
	349	161	16	28	6	19
**RvE2**	333	115	26	11	6	19
	333	253	20	11	3	11
	333	159	23	19	6	23
**RvE4**	333	115	23	50	12	39
	333	253	41	36	8	31
**AA-derived mediators**						
**LXA_4**	351	217	5	4	2	7
	351	115	14	9	4	12
	351	235	5	6	2	6
**15-epi-LXA_4**	351	217	6	5	5	17
	351	115	10	9	6	18
**LXB_4**	351	221	-	-	-	-
	351	163	-	-	-	-
**LTB_4**	335	195	7	14	6	15
	335	151	5	8	3	8
	335	129	5	7	4	9
**LTC_4**	626	301	15	21	6	20
	626	319	9	17	6	17
	626	189	52	44	13	41
**LTD_4**	497	301	10	10	4	10
	497	189	6	10	4	9
	497	241	-	-	-	-
**LTE_4**	440	189	28	34	8	19
	440	301	13	23	7	21
	440	209	-	-	-	-
	440	199	-	-	-	-
**PGD_2**	351	271	8	6	6	18
	351	189	18	22	10	29
	351	233	16	21	11	41
**PGE_2**	351	271	33	10	7	24
	351	189	10	10	9	24

	351	175	-	-	-	-
**PGF_{2a}**	353	247	7	11	3	9
	353	171	11	12	3	12
**TXB_2**	369	169	8	21	3	14
	369	195	7	19	3	12

* = the noise region was identified as the region corresponding to the retention time of the molecule of interest within an external matrix blank.
& = the noise region was identified as follows – for the relative noise algorithm this is automatically computed by the algorithm from the MRM trace; for the PtT and SD algorithms this was selected as a region immediately adjacent and of equal width to the peak of interest.
- = No peak detected; RN = Relative noise algorithm; PtP = peak to peak algorithm; SD = standard deviation algorithm.

Table S17: Different integration algorithms have little influence on the calculation of signal to noise ratios.

	MRM transitions		Autopeak			MQ4		
**DHA-derived mediators**	**Q1**	**Q3**	**RN**	**PtP**	**SD**	**RN**	**PtP**	**SD**
**RvD1**	375	233	8	2	9	9	4	9
	375	141	15	4	10	15	3	10
	375	215	8	5	12	8	5	12
	375	121	5	4	10	5	3	10
**17R-RvD1**	375	233	11	5	17	11	6	19
	375	141	21	6	19	23	6	17
	375	215	16	2	7	17	2	6
	375	121	13	4	12	12	4	10
**RvD2**	375	175	11	5	14	12	5	15
	375	215	7	4	9	7	2	8
	375	121	8	4	9	9	5	11
**RvD3**	375	147	38	8	27	41	11	33
	375	137	11	4	12	13	6	17
	375	115	-	-	-	-	-	-
**RvD4**	375	131	16	7	23	19	6	21
	375	101	27	7	22	30	6	26
	375	225	-	-	-	-	-	-
**RvD5**	359	199	19	8	18	19	8	21
	359	225	-	-	-	-	-	-
	359	141	12	5	15	13	6	17
**PD1**	359	153	11	10	29	11	8	29
	359	123	-	-	-	-	-	-
	359	137	-	-	-	-	-	-
**PDx**	359	153	16	9	29	15	11	30
	359	137	9	4	9	10	4	9
**17R-PD1**	359	153	12	7	19	17	10	27
	359	137	11	4	10	13	4	13
**22-OH-PD1**	359	204	18	8	25	17	8	23
	359	153	-	-	-	-	-	-
**PCTR1**	650	213	6	2	4	5	3	5
	650	231	10	4	7	8	_	_
	650	308	21	6	16	63	17	56
**PCTR2**	521	213	9	4	7	10	4	11
	521	325	51	16	53	57	14	63
	521	231	10	5	7	10	5	16
	521	237	8	4	7	9	2	7
**PCTR3**	464	231	7	4	6	7	3	8
	464	237	9	5	15	11	5	22
	464	245	4	2	6	4	2	7
	464	213	-	-	-	-	-	-
**MaR1**	359	221	-	-	-	-	-	-
	359	177	15	6	14	17	7	16
	359	250	32	14	37	20	10	32
**MaR2**	359	221	-	-	-	-	-	-
	359	177	-	-	-	-	-	-
	359	191	11	4	12	12	4	13
**MCTR1**	650	191	12	3	7	13	3	11
	650	205	9	3	7	10	4	13
	650	173	-	-	-	-	-	-
**MCTR2**	521	325	43	16	34	57	19	57
	521	191	16	9	13	18	10	29
	521	205	11	6	8	11	4	10
	521	173	8	6	7	11	4	10
**MCTR3**	464	191	6	5	12	7	5	11
	464	173	6	2	4	8	2	7

	464	205	8	4	7	8	4	8
**n-3 DPA-derived mediators**								
**RvT1**	377	211	14	7	21	17	7	23
	377	143	10	3	10	10	3	9
**RvT2**	377	227	25	7	25	26	8	25
	377	143	13	5	13	14	4	14
**RvT3**	361	215	-	-	-	-	-	-
	361	173	-	-	-	-	-	-
	361	155	-	-	-	-	-	-
	361	143	-	-	-	-	-	-
**RvT4**	361	143	12	6	16	14	7	19
	361	211	22	8	22	22	8	27
	361	193	9	3	9	12	4	14
**$RvD1_{n\text{-}3\ DPA}$**	377	143	10	4	12	12	5	16
	377	215	6	5	15	7	2	9
	377	233	-	-	-	-	-	-
**$RvD5_{n\text{-}3\ DPA}$**	361	199	17	6	22	21	7	19
	361	245	12	5	13	12	5	11
	361	143	13	5	18	12	6	19
**$PD1_{n\text{-}3\ DPA}$**	361	263	6	4	10	7	3	6
	361	183	7	4	12	8	4	10
	361	137	12	7	22	13	7	15
	361	155	12	7	22	13	7	15
**EPA-derived mediators**								
**RvE1**	349	195	29	5	19	32	6	18
	349	161	28	6	19	31	6	18
**RvE2**	333	115	11	6	19	12	7	19
	333	253	11	3	11	13	5	17
	333	159	19	6	23	21	6	14
**RvE4**	333	115	50	12	39	50	16	47
	333	253	36	8	31	42	9	30
**AA-derived mediators**								
**LXA_4**	351	217	4	2	7	4	2	6
	351	115	9	4	12	9	4	16
	351	235	6	2	6	7	3	8
**15-epi-LXA_4**	351	217	5	5	17	4	7	20
	351	115	9	6	18	9	8	22
**LXB_4**	351	221	-	-	-	-	-	-
	351	163	-	-	-	-	-	-
**LTB_4**	335	195	14	6	15	15	9	15
	335	151	8	3	8	9	3	9
	335	129	7	4	9	6	3	9
**LTC_4**	626	301	21	6	20	24	8	23
	626	319	17	6	17	22	5	16
	626	189	44	13	41	49	15	46
**LTD_4**	497	301	10	4	10	12	5	12
	497	189	10	4	9	11	7	12
	497	241	-	-	-	-	-	-
**LTE_4**	440	189	34	8	19	40	8	18
	440	301	23	7	21	26	10	26
	440	209	-	-	-	-	-	-
	440	199	-	-	-	-	-	-
**PGD_2**	351	271	6	6	18	8	7	21
	351	189	22	10	29	27	9	32
	351	233	21	11	41	20	11	33
**PGE_2**	351	271	10	7	24	11	9	22
	351	189	10	9	24	11	9	23
	351	175	-	-	-	-	-	-
**PGF_{2a}**	353	247	11	3	9	13	4	10

	353	171	12	3	12	14	5	11
**TXB_2**	369	169	21	3	14	25	4	16
	369	195	19	3	12	22	3	13

- = No peak detected; RN = Relative noise algorithm; PtP = peak to peak algorithm; SD = standard deviation algorithm.

Table S18: Low smoothing has limited effect on signal to noise calculations especially for low intensity peaks.

	MRM transitions		Low Smoothing		No Smoothing	
**DHA-derived mediators**	**Q1**	**Q3**	**AutoPeak**	**MQ4**	**AutoPeak**	**MQ4**
**RvD1**	375	233	8	9	7	7
	375	141	15	15	12	12
	375	215	8	8	7	9
	375	121	5	5	5	5
**17R-RvD1**	375	233	11	11	11	11
	375	141	21	23	18	19
	375	215	16	17	13	13
	375	121	13	12	10	10
**RvD2**	375	175	11	12	8	8
	375	215	7	7	7	7
	375	121	8	9	5	6
**RvD3**	375	147	38	41	24	24
	375	137	11	13	8	8
	375	115	-	-	-	-
**RvD4**	375	131	16	19	11	11
	375	101	27	30	14	14
	375	225	-	-	-	-
**RvD5**	359	199	19	19	12	12
	359	225	-	-	-	-
	359	199	12	13	9	9
**PD1**	359	153	11	11	8	8
	359	123	-	-	-	-
	359	137	-	-	-	-
**PDx**	359	153	16	15	16	15
	359	137	9	10	6	6
**17R-PD1**	359	153	12	17	12	12
	359	137	11	13	7	7
**22-OH-PD1**	359	204	18	17	14	14
	359	153	-	-	-	-
**PCTR1**	650	213	6	5	5	5
	650	231	10	8	5	5
	650	308	21	63	19	19
**PCTR2**	521	213	9	10	5	5
	521	325	51	57	33	30
	521	231	10	10	7	7
	521	237	8	9	5	5
**PCTR3**	464	231	7	7	5	5
	464	237	9	11	6	6
	464	245	4	4	4	4
	464	213	-	-	-	-
**MaR1**	359	221	-	-	-	-
	359	177	15	17	9	8
	359	250	32	20	21	21
**MaR2**	359	221	-	-	-	-
	359	177	-	-	-	-
	359	191	11	12	7	7
**MCTR1**	650	191	12	13	5	5
	650	205	9	10	7	6
	650	173	-	-	-	-
**MCTR2**	521	325	43	57	34	30
	521	191	16	18	10	10
	521	205	11	11	6	6
	521	173	8	11	6	6
**MCTR3**	464	191	6	7	6	6
	464	173	6	8	5	5

	464	205	8	8	6	6
**n-3 DPA-derived mediators**						
**RvT1**	377	211	14	17	12	12
	377	143	10	10	7	7
**RvT2**	377	227	25	26	16	16
	377	143	13	14	8	8
**RvT3**	361	215	-	-	-	-
	361	173	-	-	-	-
	361	155	-	-	-	-
	361	143	-	-	-	-
**RvT4**	361	143	12	14	11	11
	361	211	22	22	14	15
	361	193	9	12	9	9
**RvD1$_{n\text{-}3\ DPA}$**	377	143	10	12	9	9
	377	215	-	-	-	-
	377	233	-	-	-	-
**RvD5$_{n\text{-}3\ DPA}$**	361	199	17	21	16	16
	361	245	12	12	9	9
	361	143	13	12	8	8
**PD1$_{n\text{-}3\ DPA}$**	361	263	5	6	5	5
	361	183	7	7	7	7
	361	137	12	13	6	6
	361	155	-	-	-	-
**EPA-derived mediators**						
**RvE1**	349	195	29	32	14	14
	349	161	28	31	15	15
**RvE2**	333	115	11	12	9	9
	333	253	11	13	9	9
	333	159	19	21	9	10
**RvE4**	333	115	50	50	34	34
	333	253	36	42	23	23
**AA-derived mediators**						
**LXA_4**	351	217	6	6	5	5
	351	115	9	9	8	8
	351	235	6	7	4	4
**15-epi-LXA_4**	351	217	4	4	5	4
	351	115	9	9	9	9
**LXB_4**	351	221	-	-	-	-
	351	163	-	-	-	-
**LTB_4**	335	195	14	15	10	10
	335	151	8	9	5	5
	335	129	7	6	10	10
**LTC_4**	626	301	21	24	13	14
	626	319	17	22	13	13
	626	189	44	49	30	30
**LTD_4**	497	301	10	12	7	8
	497	189	10	11	7	7
	497	241	-	-	-	-
**LTE_4**	440	189	34	40	20	22
	440	301	23	26	14	16
	440	209	-	-	-	-
	440	199	-	-	-	-
**PGD_2**	351	271	6	8	6	6
	351	189	22	27	14	15
	351	233	21	20	17	17
**PGE_2**	351	271	10	11	8	9
	351	189	10	11	8	8
	351	175	-	-	-	-
**PGF_{2a}**	353	247	11	13	6	6

	353	171	12	12	6	6
**TXB_2**	369	169	21	25	12	12
	369	195	19	22	12	12

Table S19: Lipid mediator levels in human plasma blood.

Healthy Donor Plasma	Q1	Q3		Concentration (pg/mL)					
**Analyte**		Primary	Secondary	D1	D2	D3	D4	D5	D6
**DHA-derived mediators**									
RvD1	375	121	-	*	*	*	*	*	*
17R-RvD1	375	121	-	*	*	*	*	*	*
RvD2	375	175	-	*	*	*	*	*	*
RvD3	375	147	-	*	*	*	*	*	*
RvD4	375	101	-	*	*	*	*	*	*
RvD5	359	141	-	*	*	*	*	*	*
PD1	359	123	-	*	*	*	*	*	*
PDX	359	153	137	12.0	8.2	13.3	33.7	29.6	28.1
17R-PD1	359	153	-	*	*	*	*	*	*
22-OH-PD1	359	204	-	*	*	*	*	*	*
PCTR1	650	308	-	*	*	*	*	*	*
PCTR2	521	237	-	*	*	*	*	*	*
PCTR3	464	231	213	2.2	1.9	*	*	*	1.9
MaR1	359	177	250	*	*	*	*	*	*
MaR2	359	221	-	*	*	*	*	*	*
MCTR1	650	191	-	*	*	*	*	*	*
MCTR2	521	191	-	*	*	*	*	*	*
MCTR3	464	173	191	13.1	*	*	*	*	4.2
**n-3 DPA-derived mediators**									
RvT1	377	211	-	*	*	*	*	*	*
RvT2	377	227	209	*	*	*	*	*	*
RvT3	361	173	-	*	*	*	*	*	*
RvT4	361	211	193	*	5.8	*	*	*	*
$RvD1_{n\text{-}3DPA}$	377	143	-	*	*	*	*	*	*
$RvD5_{n\text{-}3DPA}$	361	199	263	*	3.3	*	*	2.0	*
$PD1_{n\text{-}3DPA}$	361	137	-	*	*	*	*	*	*
**EPA-derived mediators**									
RvE1	349	161	195	1.9	*	1.6	2.2	*	2.0
RvE2	333	115	-	*	*	*	*	*	*
RvE4	333	253	235	*	2.7	*	*	12.9	*
**AA-derived mediators**									
LXA_4	351	217	-	*	*	*	*	*	*
LXB_4	351	221	-	*	*	*	*	*	*
15-epi-LXA_4	351	217	-	*	*	*	*	*	*
LTB_4	335	195	151	1.7	*	*	*	*	*
LTC_4	626	301	319	*	*	*	*	*	*
LTD_4	497	189	-	*	*	*	*	*	*
LTE_4	440	189	301	1.0	0.8	1.2	0.7	1.4	0.5
PGD_2	351	233	189	3.0	6.6	*	1.9	2.0	7.4
PGE_2	351	189	157/271	*	1.2	*	*	*	5.9
$PGF_{2\alpha}$	353	171	193	24.3	*	15.5	41.7	23.1	29.4
TXB_2	369	169	195	16.5	30.7	17.6	26.6	47.2	462.2

Blood was collected from healthy volunteers (D1 – 36 years M, D2 = 38 years F, D3 = 32 years F, D4 = 26 years M, D5 = 43 years M, D6 = 26 years F) in sodium citrate, plasma was obtained and lipid mediators were identified and quantified.

References

1. Oddoze, C., Lombard, E. & Portugal, H. Stability study of 81 analytes in human whole blood, in serum and in plasma. *Clin Biochem* **45**, 464-469 (2012).
